# Optimal power flow of hybrid wind/solar/thermal energy integrated power systems considering renewable energy uncertainty via an enhanced weighted mean of vectors algorithm

**DOI:** 10.1371/journal.pone.0336157

**Published:** 2026-02-10

**Authors:** Ahmed H. A. Adam, Salah Kamel, Mohamed H. Hassan, Ghazally I. Y. Mustafa

**Affiliations:** 1 School of Mechanical and Electrical Engineering, Quanzhou University of Information Engineering, Quanzhou, China; 2 Department of Electrical Engineering, Faculty of Engineering, Aswan University, Aswan, Egypt; 3 Ministry of Electricity and Renewable Energy, Cairo, Egypt; SR University, INDIA

## Abstract

The rising global energy demand, along with the growth of electric power transmission and distribution systems, has intensified the need to incorporate renewable energy sources to foster sustainable development. However, achieving optimal operation within such systems poses significant challenges due to the stochastic nature of renewable energy generation. As a result, the optimal power flow (OPF) problem becomes increasingly complex when addressing the inherent uncertainty of renewable inputs. This study presents a new approach to addressing the OPF problem through the implementation of a hybrid Weighted Mean of Vectors Optimization Algorithm (INFO) based on artificial rabbits optimization (ARO) called ARINFO technique. The proposed ARINFO algorithm aims to reach an exploration-exploitation balance to improve search efficiency. To effectively manage the uncertainty associated with renewable energy output, modifications are implemented on standard test systems: in the IEEE 30-bus system (consisting of 30 buses, 6 thermal generators, and 41 branches), three thermal units are substituted with two wind turbines and one solar photovoltaic (PV) generator; a similar modification is made to the IEEE 57-bus system (which includes 57 buses, 7 thermal generators, and 80 branches) and large scale test system (IEEE 118-bus system). The stochastic characteristics of wind and solar power are modeled using Weibull and lognormal distributions, respectively. Their impact on the OPF problem is examined by incorporating reserve and penalty costs for overestimating and underestimating power output. Load demand variability is also assessed through standard probability density functions (PDF) to capture its uncertainty. Furthermore, operational constraints of thermal generators, such as ramp rate limits, are considered. The performance of the ARINFO algorithm is rigorously evaluated through 23 benchmark functions and the CEC-2022 test suite, with its effectiveness compared against nine established optimization methods. The results demonstrate that ARINFO achieved 1st rank overall on both the CEC-2017 and CEC-2022 test suites. When applied to the modified IEEE 30-bus system, ARINFO achieved a minimum generation cost of 781.1538 $/h, reduced emissions to 0.0922140 t/h, and minimized power losses to 1.734974 MW. For the larger IEEE 57-bus system, it attained a total cost of 20193.270 $/h, confirming its scalability and superior performance in managing the OPF problem under uncertainty in both generation and demand scenarios.

## 1. Introduction

### 1.1. Motivation

The concept of optimal power flow (OPF) was first introduced by Carpentier in 1962. Since that time, a multitude of methodologies have been developed to tackle the OPF problem, which is essential for minimizing power losses, enhancing voltage stability, reducing generation costs, and curbing greenhouse gas emissions. The OPF problem is typically subject to a range of physical and operational constraints, including generator capability limits, voltage bounds at buses, transmission line capacities, and permissible power flow through cables. These constraints significantly complicate the optimization process, especially in large-scale power systems, necessitating careful strategies to ensure that system parameters remain within feasible limits.

Traditionally, OPF formulations have concentrated on conventional generation sources reliant on fossil fuels, resulting in a highly complex optimization landscape characterized by mixed-integer, nonlinear, and non-convex properties. As the integration of renewable energy sources into modern power grids continues to grow, it is essential to incorporate the inherent uncertainty associated with renewables into OPF analysis, particularly during both planning and operational phases.

To address these challenges, various classical optimization techniques have been employed, including quadratic programming, nonlinear programming, mixed-integer linear programming, and interior-point methods. These approaches are appreciated for their rapid convergence rates and ability to generate optimal solutions, and many have been successfully implemented in real-world applications. However, a common limitation of these methods is their dependence on the linearization of the objective function, which may diminish their effectiveness in managing complex, nonlinear systems.

As an alternative, heuristic optimization algorithms have been proposed to overcome the limitations of traditional methods. These approaches do not require gradient information or function linearization and have demonstrated promise in solving intricate OPF problems characterized by high-dimensional and non-convex search spaces.

### 1.2. Literature review

A diverse array of meta-heuristic algorithms has been investigated in the literature to address various formulations of the optimal power flow (OPF) problem. One of the earlier approaches employed a sequential genetic algorithm (GA) combined with a simple genetic algorithm (SGA) aimed at effectively optimizing control variables while adhering to operational constraints within the system [1]. Another significant study introduced a robust Tabu Search-based method, which was validated on the IEEE 30-bus system, showcasing both reliability and computational efficiency [2]. Differential evolution (DE) has also garnered considerable attention due to its rapid convergence capabilities, making it particularly well-suited for OPF scenarios characterized by complex decision variables. However, a consistent challenge associated with DE is its tendency to converge on local optima rather than exploring the global solution space [3].

Despite the widespread application of heuristic techniques to solve OPF problems, many of these methods encounter limitations that hinder their practical effectiveness. For instance, particle swarm optimization (PSO) is valued for its ease of implementation and quick convergence; however, it is susceptible to premature convergence and may perform poorly in high-dimensional scenarios or under stringent constraints [4]. Simulated annealing (SA), while capable of escaping local optima, is highly reliant on the design of the cooling schedule and can display slow convergence rates [5]. Although ant colony optimization (ACO) proves effective for discrete problem domains, it generally converges slowly and may stagnate without adequate diversification strategies [6]. While differential evolution (DE) possesses a strong exploratory mechanism, its success heavily depends on carefully tuning mutation and scaling parameters [7]. The artificial bee colony (ABC) algorithm tends to lack sufficient exploitation strength, particularly during the final stages of optimization [8]. Additionally, newer bio-inspired methodologies such as the firefly algorithm (FA) and whale optimization algorithm (WOA) show promise, yet they often struggle with slow convergence and stagnation issues when faced with complex, multimodal search landscapes [9–11].

Numerous studies have proposed enhancements to metaheuristic optimization techniques to address challenges such as early convergence and solution quality in solving the OPF problem. A notable instance is a modified Runge-Kutta optimization algorithm introduced in [12], designed to enhance the integration of renewable energy sources (RES) into power systems. This approach accounts for the inherent intermittency and stochastic behavior of RES, particularly in the context of flexible AC transmission systems (FACTS). The study focused on minimizing three objective functions: total expected power losses (TEPL), total expected voltage deviations (TEVD), and total expected voltage stability (TEVS), with performance evaluated on the IEEE 57-bus system. In [13], a modified turbulent flow of water optimization (MTFWO) algorithm was employed to address the OPF problem within systems that include wind turbines (WT) and photovoltaic (PV) units. This approach considers the output power from renewable energy sources as a dependent variable while managing voltage levels to ensure operational balance. Simulations were conducted on the IEEE 30-bus system. The research presented in [14] introduced the white shark optimizer (WSO) as a solution for the OPF problem, aimed at minimizing fuel costs while adhering to system constraints. The integration of solar and wind energy alongside conventional thermal units was modeled using lognormal and Weibull probability distribution functions (PDFs), respectively. The algorithm’s effectiveness was illustrated through its application to the IEEE 30-bus benchmark.

A hybrid decomposition-based multi-objective evolutionary algorithm was introduced in [15] to address the OPF problem while considering WT and PV output uncertainties. The optimization aims to minimize total generation cost (TC), active power losses (APL), emissions (TE), and voltage deviation. The approach utilized a novel constraint-handling strategy along with Monte Carlo simulation for effective uncertainty modeling. The methodology was validated on the IEEE 30-bus, 57-bus, and 118-bus systems. In [16], the chaos game optimization (CGO) algorithm was utilized to tackle the OPF problem through the integration of RES and flexible AC transmission system (FACTS) devices. This method employs power generated by RES as state variables, while bus voltages are treated as control variables, with output predictions modeled using Weibull and lognormal PDFs. Validation of this approach was conducted using the IEEE 30-bus test system. Additionally, the enhanced hunter-prey optimization (EHPO) algorithm, proposed in [17], enhances exploration and exploitation capabilities for OPF challenges involving WT and FACTS integration. This algorithm incorporates random mutation and adaptive strategies to balance the global and local search phases effectively.

Moreover, in [18], a hybrid optimization framework that combines artificial ecosystem-based optimization (AEO) and chaos game optimization (CGO), known as ACGO, was developed to tackle the OPF problem with integrated RES. This method considers the uncertainties associated with wind and solar generation, employing Weibull and lognormal probability distributions. The proposed approach was validated using both the IEEE 30-bus and 57-bus systems, showcasing its effectiveness in managing OPF under uncertain conditions.

Recent advancements in addressing the stochastic optimal power flow problem have highlighted the integration of RESs, such as wind and photovoltaic (PV) systems, with modern optimization techniques. For instance, a modified runge-kutta optimizer was introduced in [12] to effectively manage uncertainties in power systems that incorporate wind, PV, and FACTS like TCSC. This study showcased significant improvements in minimizing power losses and voltage deviations using the IEEE 57-bus system under stochastic conditions. Additionally, an enhanced version of the gorilla troops optimizer, as described in [19], utilized chaotic-quasi-oppositional learning and multi-population strategies to improve the balance between exploration and exploitation in optimal power flow problems. This method exhibited robust convergence and solution quality across various objectives, including fuel cost optimization and voltage stability.

Collectively, these studies underscore a trend towards biologically inspired, hybrid metaheuristic strategies that effectively tackle the nonlinear and non-convex challenges inherent in SOPF problems involving integrated renewable energy and uncertainty modeling. These insights not only enhance the current methodological landscape but also reinforce the relevance of the proposed ARINFO algorithm.

The increasing integration of renewable energy sources into power grids poses a significant challenge in maintaining the stability and efficiency of modern electrical systems. Sources like wind and solar introduce substantial variability and uncertainty into power flow, complicating the optimal operation of power systems. Consequently, traditional methods for solving the OPF problem must be adapted to account for these uncertainties. Despite advancements in metaheuristic applications for OPF issues, effectively managing the inherent uncertainties of RES, such as wind and solar, is a considerable challenge. The stochastic nature of RES generation leads to complexities associated with both overestimation and underestimation of power, making it essential to include reserve and penalty costs in the optimization model. Additionally, traditional algorithms often struggle to maintain a balanced equilibrium between exploration and exploitation, resulting in premature convergence or suboptimal solutions in high-dimensional, non-convex search spaces.

This study addresses the OPF problem within the framework of hybrid energy systems that combine renewable sources with conventional thermal units. The objective is to optimize overall power generation while minimizing both costs and emissions. By incorporating uncertainty modeling for renewable generation, this research proposes a novel optimization algorithm to manage hybrid power systems’ inherent complexities. In particular, the study aims to deliver a robust and efficient technique for solving the OPF problem while explicitly considering uncertainties in both renewable generation and load demand.

### 1.3. Importance of investigation

As highlighted by previous studies, various optimization techniques have been effectively employed to tackle engineering challenges, particularly in OPF problems. These studies underscore the success of such methods and the ongoing necessity of developing more robust and efficient optimization strategies tailored to specific OPF scenarios. Moreover, the No-Free-Lunch (NFL) theorem [20] illustrates that no single metaheuristic algorithm can universally solve all optimization problems. This theoretical constraint reinforces the importance of designing novel algorithms.

To achieve the global optimum while avoiding the pitfalls of local optima, various strategies can be employed. Effective algorithmic planning that promotes intelligent scheduling of transitions between exploration and exploitation phases, along with establishing an appropriate equilibrium between these phases, is among the viable approaches. Adequate scheduling and balancing can mitigate the accuracy issue to a degree; nevertheless, the most potent method involves integrating the most appropriate variety throughout the solution process. Therefore, hybridization is regarded as a robust strategy that raises the necessary diversity in the quest for the global optimum, thereby minimizing the likelihood of becoming entrenched at a local optimum point [21]. As a result, one of the objectives of this study is to introduce a hybrid algorithm designed to enhance solution accuracy while maintaining high solution diversity throughout the entire solution process.

Based on these insights, this study introduces a novel optimization method, ARINFO, which enhances the weighted mean of vectors (INFO) algorithm. Utilizing INFO’s robust global search capabilities in conjunction with ARO’s effective local refinement, the hybrid approach adeptly balances exploration and exploitation. This combined framework harnesses the advantages of both algorithms, enhancing both extensive exploration and rapid local refinement abilities. During the global exploration phase, INFO plays a crucial role, while ARO is activated during the local refinement phase. At the beginning of each iteration, an update to the population is executed using INFO. Subsequently, in the middle of the iteration, local refinement occurs in the vicinity of the optimal solution with ARO, and finally, at the conclusion of the iteration, the optimal solution is revised. Consequently, the search remains expansive, while refinement is concentrated around the optimal solution. This methodology ensures that INFO’s global exploration capability significantly boosts the likelihood of discovering superior solutions across a vast solution space. Furthermore, ARO’s local search capability facilitates a more rapid enhancement of the solution. The integration of these two algorithms results in enhanced solution quality and reduced optimization duration. This adaptive framework enables the execution of both global and local searches, allowing for adjustments to evolving optimization conditions. The proposed approach aims to reduce power losses, improve voltage stability, lower generation costs, and mitigate gas emissions. The choice of the underlying algorithm is informed by its proven effectiveness in addressing complex mathematical and engineering design challenges across various domains.

### 1.4. Main contributions

While the original INFO optimizer demonstrates strong convergence speed and promising performance through its mean-based update rule, vector combination, and local search strategies, it also has several limitations that an enhanced version can address [22]. Notably, INFO’s dependence on the weighted mean and fixed procedures may result in premature convergence in complex or multimodal problems due to inadequate mechanisms for maintaining population diversity over time. Furthermore, although INFO includes a local search step, its structure is relatively simplistic and deterministic, which may hinder its effectiveness in fine-tuning solutions or escaping difficult local optima. Additionally, INFO does not incorporate adaptive control or feedback mechanisms to dynamically balance exploration and exploitation throughout the optimization process, which can diminish its robustness across varied problem landscapes [23]. These shortcomings lay the groundwork for enhancing INFO by introducing mechanisms that promote adaptability, diversity maintenance, and improved local refinement, thereby representing the core contributions of the proposed work.

This study makes several significant contributions to the field of OPF optimization:

- A novel and enhanced variant of the ARINFO algorithm is introduced. This improved algorithm tackles OPF challenges by minimizing power losses, enhancing voltage stability, reducing generation costs, and limiting greenhouse gas emissions.
- The effectiveness of the ARINFO algorithm is rigorously validated through a comprehensive evaluation using 23 standard benchmark functions and 12 functions from the CEC-2022 test suite.
- A thorough performance assessment of ARINFO is conducted, employing statistical measures, analyzing convergence behavior, performing boxplot comparisons, and executing qualitative evaluations.
- The results generated by ARINFO across all test cases are compared against nine well-established metaheuristic algorithms, including the original INFO algorithm [23], artificial hummingbird algorithm (AHA) [24], artificial rabbits optimization (ARO) [25], whale optimization algorithm (WOA) [26], spider wasp optimizer (SWO) [27], particle swarm optimization (PSO) [28], moth–flame optimization (MFO) [29], seagull optimization algorithm (SOA) [30], and sine cosine algorithm (SCA) [31].
- Furthermore, the IEEE 30-bus system is modified to include renewable energy sources, specifically wind and solar units. ARINFO, in conjunction with AHA, ARO, PSO, SOA, MFO, and the original INFO algorithm, is employed to address the OPF problem under four distinct objective functions.
- Practical case studies are undertaken that consider generation and demand uncertainty, as well as ramp rate constraints for thermal power units. The outcomes from ARINFO are compared with those from alternative methods within these scenarios to assess its robustness and applicability in real-world situations.
- A modified IEEE 57-bus test system and an IEEE 118-bus test system are utilized to evaluate the proposed method’s scalability and generalizability.

The findings provide strong evidence that the proposed ARINFO algorithm exhibits superior performance compared to the conventional INFO algorithm in addressing the OPF problem, particularly in terms of solution quality and convergence behavior.

### 1.5. Structure of the paper

The remainder of this paper is organized as follows: Section 2 introduces the formulation of the OPF problem. Section 3 examines the constraints associated with the OPF model. Section 4 delves into various objective functions pertinent to OPF optimization. Section 5 details the development of the proposed ARINFO optimization framework. Section 6 presents simulation results along with a critical discussion. Section 7 illustrates the practical implementation of the ARINFO framework within the context of OPF. The final section provides concluding remarks and offers suggestions for future research directions.

## 2. Problem formulation of optimal power flow

The OPF problem is a mathematical optimization framework aimed at identifying the most efficient operating conditions for power system components while adhering to all relevant constraints. Generally, the OPF seeks to optimize a specific objective function, such as minimizing generation costs or power losses, subject to equality and inequality constraints that reflect physical and operational limits. In essence, the OPF problem can be formulated in the following mathematical structure [14]:


MinimizeF(x,u) (1)

Subject to


$$\begin{array}{c} g_{i}\left(x,\;u\right)=0\;\;\;\;i=1,2,3,......,m\\ h_{j}\left(x,\;u\right)\leq 0\;\;\;\;j=1,2,3,......,n \end{array}$$ (2)

In this context, *F* represents the objective function, *x* signifies the vector of state variables, and *u* corresponds to the vector of control variables. The functions *g*_*i*_ and *h*_*j*_ denote the equality and inequality constraints, respectively, while *m* and *n* represent the total numbers of equality and inequality constraints in the system [14].

The state variables, also referred to as dependent variables, can be represented in vector form as follows:


$$x=\left[P_{G1},\;V_{L1}...\;\;V_{LNPQ},\;Q_{G1}...\;\;Q_{GNPV},\;S_{TL1}...\;\;\;S_{TLNTL}\right]$$ (3)

where *P*_*G*1_ represents the active power output of the slack bus, while *V*_*L*_ denotes the voltage magnitude at the load buses. *NPQ* is the total number of load buses, and *Q*_*G*_ refers to the reactive power outputs of the generators, with *NPV* indicating the number of generator buses. *S*_*TL*_ signifies the apparent power flow through the transmission lines, and *NTL* represents the total number of transmission lines.

The control variables, also referred to as independent variables, can be organized in a vector format as follows:


$$u=\left[P_{G2}...\;\;P_{GNG},\;V_{G1}...\;\;V_{GNG},\;Q_{C1}...\;\;Q_{CNC},\;T_{1}...\;\;\;T_{NT}\right]$$ (4)

Here, *P*_*G*_ represents the active power output from the generators, while *NG* indicates the total count of generators. *V*_*G*_ refers to the voltage magnitude at the generator buses. *Q*_*C*_ signifies the reactive power contributed by shunt compensators, with *NC* denoting the total number of these compensators. *T* represents the tap settings of transformers, and *NT* corresponds to the overall number of transformers within the system.

This section presents a comprehensive analysis of the production costs associated with each power source in the IEEE 30-bus system. As the production cost methodology for the IEEE 57-bus system is the same, it will not be detailed here.

### 2.1. Thermal power generation cost

The costs associated with thermal power generation (TPG) are assessed using equation (5), which accounts for the valve-point effect of thermal units to improve cost estimation accuracy [32].


$$C_{T}(P_{TG})=\sum_{i=1}^{N_{TG}}a_{i}+b_{i}P_{TGi}+c_{i}P_{TGi}^{2}+\left|d_{i}*\sin\left(e_{i}*\left(P_{TGi}^{\min}-P_{TGi}\right)\right)\right|$$ (5)

where *P*_*TGi*_ denotes the output power of the *i*^*th*^ TPG. The coefficients *a*_*i*_, *b*_*i*_, and *c*_*i*_ are associated cost coefficients. Additionally, *d*_*i*_ and *e*_*i*_ represent the valve-point effect coefficients linked to each *TPG*. *N*_*TG*_ indicates the total number of thermal generators in the system, while *P*_*TGi*_^*min*^ marks the minimum generation limit for the *i*^*th*^ TPG. In Appendix A, Table A1 in S1 File provides the cost coefficients related to TPGs [14].

### 2.2. Cost components of wind power generation

Unlike TPG, wind power generation (WPG) is characterized by considerable variability and uncertainty. As a result, the costs associated with wind energy production are calculated using a distinct methodology, as detailed below.

a. *Direct Cost Component*

The direct cost of power generated by WPG, in relation to its scheded output, is determined using the following expression:


$$C_{dwj}=h_{wj}P_{swj}$$ (6)

where *P*_*swj*_ refers to the scheduled power output of the *j*^*th*^ wind power plant, while *h*_*wj*_ indicates its corresponding direct cost coefficient.

b. *Uncertain Cost Component*

Due to the inherent variability of WPG, actual output may often fall short of the scheduled values, leading to an overestimation of power availability. In these situations, the ISO is required to compensate for the shortfall by utilizing adequate spinning reserves [33]. The costs associated with maintaining these reserves are quantified as follows:


$$C_{re.wj}\left(P_{swj}-P_{ava.wj}\right)=K_{reswj}\left(P_{swj}-P_{wind.j}\right)=K_{rw.j}\int {\mathrm{\backslash nolimits}}_{0}^{P_{swj}}\left(P_{swj}-P_{wind.j}\right)\times f_{wind.j}\left(P_{wind.j}\right)dP_{wind.j}$$ (7)

In this context, *K*_*reswj*_ refers to the reserve cost coefficient associated with the *j*^*th*^ wind power plant, while *P*_*ava.wj*_ represents the actual power output produced by that unit. The probability density function (PDF) for the wind power output of the *j*^*th*^ wind plant is denoted by *f*_*wind.j*_.

On the other hand, the actual power output from a WPG may surpass its scheduled value, a situation known as underestimation. In these instances, the ISO faces a penalty cost for the excess generation, calculated as follows:


$$C_{pen.wj}\left(P_{ava.wj}-P_{swj}\right)=K_{penwj}\left(P_{ava.wj}-P_{swj}\right)=K_{penwj}\int {\mathrm{\backslash nolimits}}_{P_{swj}}^{P_{rated.wj}}\left(P_{wind.j}-P_{swj}\right)\times f_{wind.j}\left(P_{wind.j}\right)dP_{wind.j}$$ (8)

In this context, *K*_*penwj*_ denotes the penalty cost coefficient, while *P*_*rated.wj*_ represents the rated power of the *j*^*th*^ wind power plant.

### 2.3. Probabilistic characterization of wind power generation

This section discusses the probabilistic modeling of power output from wind plants, represented by the term *f*_*wind.j*_(*P*_*wind.j*_) in (7) and (8). The Weibull PDF is commonly used to characterize wind speed distributions [32]. Consequently, the probability of wind speed is evaluated using the Weibull distribution, as defined by the following expression:


$$f_{Wd_{v}}\left(Wd_{v}\right)=\left(\frac{k}{c}\right)+{\left(\frac{Wd_{v}}{c}\right)}^{\left(k-1\right)}e^{-{\left(Wd_{v}/c\right)}^{k}}\;\;\;\;\;\;\;\;\;\;\;\;\;\;\;\;\;\;\;\;for0<Wd_{v}<\infty$$ (9)

where *Wd*_*v*_ represents the wind speed measured in (m/s), while *k* and *c* signify the shape and scale parameters of the Weibull distribution, respectively. The mean wind speed corresponding to the Weibull probability density function is calculated using the following expression:


$$M_{weibull}=c\times \Gamma \left(1+k^{-1}\right)$$ (10)

In this expression, Γ denotes the gamma function, which is defined mathematically as follows:


$$\Gamma \left(x\right)=\int {\mathrm{\backslash nolimits}}_{0}^{\infty }e^{-t}t^{x-1}dt$$ (11)

This study involves modifications to the IEEE 30-bus test system, which include replacing two thermal generators situated at buses 5 and 11 with wind turbine generators. Additionally, the thermal generator at bus 13 has been substituted with a solar photovoltaic (PV) unit. In Appendix A, the parameters for the Weibull distribution, specifically the scale (*c*) and shape (*k*) values used for modeling wind speed, are presented in Table A2 in S1 File, while the wind frequency distribution, obtained through an 8000-iteration Monte Carlo simulation, is depicted in Fig A1 in S1 File, showcasing the precision of the Weibull fit [32].

### 2.4. Power models for wind turbine generators

As previously mentioned, the modified IEEE 30-bus power system includes two WPGs. WPG1, situated at bus 5, has a rated active power output of 75 MW, while WPG2, connected at bus 11, has a capacity of 60 MW. The relationship between wind speed and the resulting power output of these generators is described by the following equation [14]:


$$P_{wind}\left(Wd_{v}\right)\{\begin{array}{c} 0\;\;\;\;\;\;\;\;\;\;\;\;\;\;\;\;\;\;\;\;\;\;for\;\;Wd_{v}\leq Wd_{vin}\;and\;\;Wd_{v}>Wd_{vout}\\ P_{rated.w}*\left(\frac{\left(Wd_{v}-Wd_{vin}\right)}{\left(Wd_{vr}-Wd_{vin}\right)}\right)\;for\;\;Wd_{vin}\leq Wd_{v}\leq Wd_{vr}\\ P_{rated.w}\;\;\;\;\;\;\;\;\;\;\;\;\;\;\;\;\;\;\;\;\;\;\;\;\;\;\;\;\;\;for\;\;Wd_{vr}\leq Wd_{v}\leq Wd_{vout} \end{array}$$ (12)

where *Wd*_*vin*_ represents the cut-in wind speed, *Wd*_*vout*_ denotes the cut-out wind speed, and *Wd*_*vr*_ indicates the rated wind speed. *P*_*rated.w*_ corresponds to the rated power output of the wind turbine. The probability of the wind power output falling within a specific discrete range can be calculated using the following expression:


$$f_{wind}\left(P_{wind}\right)\left\{P_{wind}=0\right\}=1-exp\left[-{\left(\frac{Wd_{vin}}{c}\right)}^{k}\right]+exp\left[-{\left(\frac{Wd_{vout}}{c}\right)}^{k}\right]$$ (13)

$$f_{wind}\left(P_{wind}\right)\left\{P_{wind}=P_{rated.w}\right\}=exp\left[-{\left(\frac{Wd_{vin}}{c}\right)}^{k}\right]-exp\left[-{\left(\frac{Wd_{vout}}{c}\right)}^{k}\right]$$ (14)

For the continuous operating range, the probability of the wind farm’s power output can be assessed with the following equation:


$$f_{wind}\left(P_{wind}\right)=\frac{k\left(Wd_{vr}-Wd_{vin}\right)}{c^{k}\times P_{rated.w}}{\left[Wd_{vin}+\frac{P_{wind}}{P_{rated.w}}\left(Wd_{vr}-Wd_{vin}\right)\right]}^{k-1}\times exp\left[-{\left(\frac{Wd_{vin}+\frac{P_{wind}}{P_{rated.w}}\left(Wd_{vr}-Wd_{vin}\right)}{c}\right)}^{k}\right]$$ (15)

### 2.5. Cost components of solar power generation

The total cost of electricity generated by a solar photovoltaic (PV) system consists of two primary components: the direct generation cost and the costs related to output uncertainty. This cost structure is similar to that employed for wind energy systems [23].

a. *Direct Cost Component*

The direct cost of solar energy production is calculated using the following equation:


$$C_{d.sk}=g_{sk}P_{ssk}$$ (16)

where *P*_*ssk*_ represents the scheduled power output of the *k*^*th*^ solar power plant, while *g*_*sk*_ denotes the direct cost coefficient linked to that unit.

b. *Uncertain Cost Component*

Similar to wind power systems, the cost analysis of solar power generation considers both scenarios of overestimation and underestimation [34]. A reserve cost is incurred when the predicted solar output surpasses the actual generation. This reserve cost linked to overestimation is determined using the following expression:


$$C_{res.sk}\left(P_{ssk}-P_{ava.sk}\right)=K_{ressk}\left(P_{ssk}-P_{ava.sk}\right)=K_{ressk}\times f_{solar.k}\left(P_{ava.sk}<P_{ssk}\right)\times \left[\left(P_{ssk}-Ex\left(P_{ava.sk}<P_{ssk}\right)\right)\right]$$ (17)

In this formulation, *K*_*ressk*_ denotes the reserve cost coefficient for the kth solar power plant, while *P*_*ava.sk*_ represents its available power output. The term *f*_*solar.k*_(*P*_*ava.sk* _< *P*_*ssk*_) captures the probability that the plant’s actual output falls short of the scheduled value *P*_*ssk*_. Meanwhile, *Ex*(*P*_*ava.sk* _< *P*_*ssk*_) refers to the expected magnitude of this shortfall [35]. The penalty incurred for underestimating solar power production is quantified by the following equation:


$$C_{pen.sk}\left(P_{ava.sk}-P_{ssk}\right)=K_{pensk}\left(P_{ava.sk}-P_{ssk}\right)=K_{pensk}\times f_{solar.k}\left(P_{avask}>P_{ssk}\right)\times \left[\left(Ex\left(P_{ava.sk}>P_{ssk}\right)-P_{ssk}\right)\right]$$ (18)

where *K*_*pensk*_ represents the penalty cost coefficient associated with the *k*^*th*^ solar plant. The term *f*_*solar.k*_(*P*_*ava.sk* _> *P*_*ssk*_) indicates the probability that the actual solar power output surpasses the scheduled generation. Additionally, *Ex*(*P*_*ava.sk* _< *P*_*ssk*_) denotes the expected surplus output from the *k*^*th*^ solar plant [36,37].

### 2.6. Probabilistic characterization of solar power plants

The power output of a solar photovoltaic generator is mainly determined by solar irradiance (*I*), which is often modeled using a lognormal PDF. Consequently, the statistical behavior of solar irradiance can be represented by the following lognormal distribution :


$$f_{I}\left(I\;\right)=\frac{\text{1}}{I\sigma \sqrt{2\pi }}\;exp\left\{\frac{-{\left(\ln x-\mu \right)}^{2}}{2\sigma^{2}}\right\}\;\;\;\;for\;\;I>0$$ (19)

Let *f*_*I*_(*I*) denote the probability density function of solar irradiance. This irradiance is modeled using a lognormal distribution, which is defined by its mean *μ* and standard deviation *σ*. The mean value of the lognormal distribution, represented as *M*_*lgn*_, is calculated using the following expression:


$$M_{lgn}=exp\left(\mu +\frac{\sigma^{2}}{\text{2}}\right)$$ (20)

In Appendix A, Fig A2 in S1 File illustrates the frequency distribution alongside the lognormal probability curve for solar irradiance based on an 8000-iteration Monte Carlo simulation. The specific parameter values utilized in the lognormal probability density function for this analysis are detailed in Table A2 in Appendix A in S1 File.

### 2.7. Power models for solar photovoltaic systems

The electrical power produced by solar photovoltaic systems is directly affected by the intensity of solar irradiance (*I*). Consequently, the relationship between solar radiation and the output power of a photovoltaic system can be mathematically expressed as follows [32]:


$$P_{solar}\left(v\right)=\{\begin{array}{c} {}*20lP_{solarr}\left(\frac{I^{2}}{I_{std}T_{c}}\right)\;\;\;\;\;\;for\;\;0<I\;<T_{c}\\ P_{solarr}\;\left(\frac{I}{I_{std}}\right)\;\;\;\;\;\;\;\;for\;I\geq T_{c} \end{array}$$ (21)

In this expression, *I*_*std*_ signifies the standard solar irradiance level, which is typically established at 800 W/m², while *T*_*c*_ represents a specific threshold irradiance value set at 120 W/m². The term *P*_*solarr*_ refers to the rated capacity of the solar photovoltaic system.

The reserve cost linked to solar power, as detailed in equation (17), can be reformulated by integrating the probability distribution of solar power availability as follows:


$$C_{res.sk}\left(P_{ssk}-P_{ava.sk}\right)=K_{ressk}\left(P_{ssk}-P_{ava.sk}\right)=K_{ressk}\sum_{n=1}^{N^{-}}\left[P_{ssk}-P_{sn-}\right]*fsn-$$ (22)

where *P*_*sn−*_ represents the shortfall in solar power, defined as the amount of actual output that falls below the scheduled generation level. This is depicted on the left-hand side of the distribution curve for scheduled solar power output (*P*_*ssk*_) in Fig A3 (Appendix A) in S1 File. The notation *f*_*sn−*_ refers to the relative frequency associated with instances of this shortfall, while *N*^*−*^ denotes the number of discrete intervals (bins) within this underperformance region [38].

Furthermore, the penalty cost expression previously introduced in equation (18) can be reformulated as follows:


$$C_{pen.sk}\left(P_{ava.sk}-P_{ssk}\right)=K_{pensk}\left(P_{ava.sk}-P_{ssk}\right)=K_{pensk}\sum_{n=1}^{N^{+}}\left[P_{sn+}-P_{ssk}\right]*fsn+$$ (23)

The variable *P*_*sn+*_ denotes the surplus solar power, which refers to the amount of generated power that exceeds the scheduled output. This condition is illustrated in the right-hand section of the distribution curve for expected solar power output (*P*_*ssk*_) shown in Fig A3(Appendix A) in S1 File. The variable *f*_*sn+*_ represents the relative frequency of these surplus occurrences, while *N*+ indicates the number of discrete intervals (bins) within this region of excess generation.

### 2.8. Emission

Electricity generation from conventional fossil fuel sources significantly contributes to greenhouse gas emissions. Among the most harmful byproducts are sulfur oxides (SOₓ) and nitrogen oxides (NOₓ) [12]. The levels of these pollutants typically correlate with the amount of electrical power produced by thermal power plants. This relationship, which quantifies emissions in (t/h) as a function of generated output in (p.u. MW), is mathematically represented in equation (24):


$$E=\sum_{i=1}^{N_{TG}}\left[\left(\alpha_{i}+\beta_{i}P_{TG.i}+\gamma_{i}P_{TGi}^{2}\right)\times 0.01+\omega_{i}e^{\left(\mu_{i}P_{TGi}\right)}\right]$$ (24)

In this formulation, the coefficients *α*_*i*_*, β*_*i*_*, γ*_*i*_*, ω*_*i*_*,* and *μ*_*i*_ represent the emission parameters associated with TPGs. The specific values assigned to these coefficients are detailed in Table 1A (Appendix A in S1 File) and align with the data referenced in [24]. The total emissions, measured in metric tonnes, are then converted into an economic cost, referred to as *E*_*C*_ (in $/h), as defined in equation (25). In this context, *C*_*tax*_ denotes the carbon tax rate, expressed in *$/tonne*.


$$E_{C}=E\times C_{tax}$$ (25)

## 3. Problem constraints

The optimal power flow problem is governed by a set of operational constraints that are essential for ensuring the system operates effectively. These constraints are classified into two primary categories: equality and inequality.

### 3.1. Equality constraints

Equality constraints are primarily designed to maintain a power balance within the system. They stipulate that the total generated active and reactive power must equal the sum of consumed power (including both active and reactive power) as well as the losses within the transmission system [39].


$$P_{Gi}=P_{Di}+V_{i}\sum_{i=1}^{NB}V_{j}[G_{ij}\cos\left(\delta_{ij}\right)+B_{ij}\sin\left(\delta_{ij}\right);\;\;\;\forall i\in NB$$ (26)

$$Q_{Gi}=Q_{Di}+V_{i}\sum_{i=1}^{NB}V_{j}[G_{ij}\sin\left(\delta_{ij}\right)+B_{ij}\cos\left(\delta_{ij}\right);\;\;\;\;\;\forall i\in NB$$ (27)

In this context, *NB* signifies the total number of buses within the power system. The variables *P*_*D*_ and *Q*_*D*_ correspond to the active and reactive components of the load demand, respectively, while *P*_*G*_ and *Q*_*G*_ represent the active and reactive power generated. The voltage angle difference between buses *i* and *j* is denoted by *δ*_*ij*_, with *B*_*ij*_ and *G*_*ij*_ representing the susceptance and conductance of the transmission line that connects the two buses.

### 3.2. Inequality constraints

Inequality constraints are essential for enforcing the operational limits of power system components and ensuring the overall security of the network. These constraints define the permissible operating ranges for generation units, transmission lines, and load buses. They can be systematically categorized as follows [14]:

a. *Generator Restrictions*

Each generator’s operation within the power system is restricted by established lower and upper limits. These constraints pertain to active power output, reactive power output, and generator voltage levels, as outlined in equations (28), (29), and (30), respectively. In this context, *N*_*TG*_ represents the total number of generators in the network [12].


$$P_{TGi}^{\min}\leq P_{TGi}\leq P_{TGi}^{\max}\;\;\;\;\;\;\;\;\;\;\;\;\forall i\in N_{TG}$$ (28)

$$Q_{TGi}^{\min}\leq Q_{TGi}\leq Q_{TGi}^{\max}\;\;\;\;\;\;\;\;\;\;\;\forall i\in N_{TG}$$ (29)

$$V_{TGi}^{\min}\leq V_{TGi}\leq V_{TGi}^{\max}\;\;\;\;\;\;\;\;\;\;\;\;\;\forall i\in N_{TG}$$ (30)

In addition, the generation limits for renewable energy sources, specifically wind turbines and solar photovoltaic (PV) units, are defined by equations (31) through (34). These equations outline the permissible ranges for their active and reactive power outputs.


$$P_{Ws.j}^{\min}\leq P_{Ws.j}\leq P_{Ws.j}^{\max}$$ (31)

$$P_{Ss.k}^{\min}\leq P_{Ss.k}\leq P_{Ss.k}^{\max}$$ (32)

$$Q_{Ws.j}^{\min}\leq Q_{Ws.j}\leq Q_{Ws.j}^{\max}$$ (33)

$$Q_{Ss.k}^{\min}\leq Q_{Ss.k}\leq Q_{Ss.k}^{\max}$$ (34)

b. *Transformer Restrictions*

$$T_{t}^{\min}\leq T_{t}\leq T_{t}^{\max},\;\;\;\;t=1............,N_{T}$$ (35)

c. *Shunt Compensator Restrictions*

$$Q_{Cc}^{\min}\leq Q_{Cc}\leq Q_{Cc}^{\max},\;\;\;c=1,..........,N_{C}$$ (36)

where *N*_*TG*_, *N*_*T*_, and *N*_*C*_ represent the total number of power generators, power transformers, and shunt compensators, respectively.

d. *Ramp Rate Restrictions for TGs*

Within the framework of optimal power flow analysis, ramp-rate constraints for thermal generators are imposed to restrict the permissible rate of change in power output between successive time intervals, expressed formally as follows:


$$P_{TGi}-P_{TGi}^{0}\leq U_{ri},\;\;\;\;\;\;\;if\;power\;generation\;rises$$ (37)

$$P_{TGi}^{0}-P_{TGi}\leq D_{ri},\;\;\;\;\;\;\;\;\;if\;power\;generation\;reduces$$ (38)

where *P*^*0*^_*TGi*_ refers to the output power of the *i*^*th*^ thermal generator during the previous time interval. The parameters *U*_*ri*_ and *D*_*ri*_ represent the upper and lower ramp-rate limits, respectively, which dictate the permissible changes in output for that particular generator [40,41].

e. *Transmission Line Capacity Constraints*

Power flow through transmission lines must remain within established thermal and operational limits to ensure system stability and safety. This requirement is mathematically expressed in equation (39), where *N*_*L*_ indicates the total number of transmission lines in the electrical network.


$$S_{Lq}\leq S_{L_{q}}^{\max},\;\;q=1,..........,N_{L}$$ (39)

f. *Load Bus Voltage Restrictions*

The voltage levels at load buses are subject to predefined lower and upper bounds to ensure stable and secure system operation. These limits can be expressed as:


$$V_{i}^{\min}\leq V_{i}\leq V_{i}^{\max}\;\;\;\;\;\;\;\forall \;i\in N_{LB}$$ (40)

where *N*_*LB*_ represents the total number of load buses in the network.

An additional critical metric associated with load buses is the voltage deviation, which quantifies the deviation of each load bus voltage from the nominal value (typically 1.0 p.u.). It is calculated using the following expression:


$$V_{d}=\sum_{i=1}^{N_{LB}}\left|V_{i}-1\right|$$ (41)

This parameter serves as an indicator of voltage quality across the system, with lower values signifying better adherence to the nominal voltage level.

## 4. OPF objective functions

The OPF framework presented in this study aims to optimize various performance metrics, focusing on the minimization of total generation costs (both with and without the application of carbon emission taxes), the reduction of greenhouse gas emissions, and the minimization of active power losses. These objectives are approached through distinct mathematical formulations.

### 4.1. Case 1: Minimization of Total Generation Cost Without Carbon Tax (*F*_1_)

The objective function *F*_1_ reflects the total production cost without carbon taxation. This is formulated by aggregating the cost components outlined in the “OPF problem” section. Consequently, the expression for *F*_1_ is defined as follows:


$$\begin{array}{c} F_{1}=Minimize(C_{T}(P_{TG})+\sum_{j=1}^{N_{WG}}(h_{wj}P_{swj}+K_{rwj}(P_{swj}-P_{wind.j})+K_{penwj}(P_{ava.wj}-P_{swj}))\\ +\sum_{j=1}^{N_{SG}}\left(g_{sk}P_{ssk}+K_{ressk}\left(P_{ssk}-P_{ava.sk}\right)+K_{pensk}\left(P_{ava.sk}-P_{ssk}\right)\right)) \end{array}$$ (42)

### 4.2. Case 2: Minimization of Total Generation Cost with Carbon Tax (*F*_2_)

The objective function *F*_2_ enhances the cost formulation presented in *F*_1_ by integrating the costs associated with carbon emissions, as defined in equation (25). Therefore, the total cost under carbon taxation is represented by the following expression:


$$\begin{array}{c} F_{2}=Minimize(C_{T}(P_{TG})+\sum_{j=1}^{N_{WG}}(h_{wj}P_{swj}+K_{rwj}(P_{swj}-P_{wind.j})+K_{penwj}(P_{ava.wj}-P_{swj}))\\ +\sum_{j=1}^{N_{SG}}\left(g_{sk}P_{ssk}+K_{ressk}\left(P_{ssk}-P_{ava.sk}\right)+K_{pensk}\left(P_{ava.sk}-P_{schsk}\right)\right)+\left[E\times C_{tax}\right]) \end{array}$$ (43)

### 4.3. Case 3: Minimization of Carbon Emissions (F_3_)

The objective function *F*_3_ concentrates on decreasing the total carbon emissions produced by thermal power units. It is formulated according to the emission model detailed in equation (24) and can be mathematically expressed as follows:


$$F_{3}=Minimize\left(\sum_{i=1}^{N_{TG}}\left[\left(\alpha_{i}+\beta_{i}P_{TG.i}+\gamma_{i}P_{TGi}^{2}\right)\times 0.01+\omega_{i}e^{\left(\mu_{i}P_{TGi}\right)}\right]\right)$$ (44)

### 4.4. Case 4: Minimization of Active Power Losses

The objective function in this scenario, denoted as *F*_4_, aims to minimize active power losses within the power system. These losses are quantified using equation (45), which models power dissipation across transmission lines.


$$P_{loss}=\sum_{i=1}^{NL}\sum_{j\neq 1}^{NL}\left(G_{ij}\times \left[V_{i}^{2}+V_{j}^{2}-2V_{i}V_{j}\cos(\delta_{ij})\right]\right)$$ (45)

In this formulation, *δ*_*ij*_ represents the voltage angle difference between buses *i* and *j*, *N*_*L*_ is the total number of transmission lines, and *G*_*ij*_ denotes the conductance of the line connecting the two buses. Based on this, the objective function *F*_4_ is defined as:


$$F_{4}=Minimize\left(\sum_{i=1}^{NL}\sum_{j\neq 1}^{NL}\left(G_{ij}\times \left[V_{i}^{2}+V_{j}^{2}-2V_{i}V_{j}\cos\left(\delta_{ij}\right)\right]\right)\right)$$ (46)

## 5. Development of the Proposed ARINFO optimization framework

### 5.1. Overview of the Weighted Mean of Vectors Algorithm (INFO)

The INFO algorithm, introduced in [42], offers a novel optimization framework based on a modified version of the weighted mean of vectors method. As a recently developed metaheuristic, INFO utilizes a vector-based update mechanism along with a local search strategy to navigate the solution space effectively [22]. The algorithm proceeds through the following sequential phases:

- Updating rule stage
- Vector combination
- Local search execution
***Initialization phase***

The initial population for the INFO algorithm is generated randomly and is represented as follows:


$$X_{l,j}=\left\{X_{l,1},X_{l,2},.......,X_{l,D}\right\},l=1,2,.....,Np$$ (47)

Here, *Np* denotes the population size, while *D* represents the dimensionality of the optimization problem. Two key parameters play a crucial role in the vector updating mechanism within INFO: the scaling factor (*σ*), which adjusts the magnitude of the weighted mean vector, and the weighted mean factor (*δ*), which enhances the influence of the generated vector during the update process.

ii. ***Updating rule stage***

During this phase, weighted mean vectors are computed using randomly selected differential vectors. The MeanRule, a mean-based updating mechanism, is applied to adjust the positions of solution vectors. This rule incorporates information from the worst, a randomly chosen better (selected from the top five performing solutions), and the best solutions within the population. The MeanRule formulation is expressed as follows [22]:


$$MeanRule=r\times WM_{l}^{g}+\left(1-r\right)\times WM2_{l}^{g}\;\;\;\;\;\;\;\;\;\;\;\;\;\;l=1,2,.......,Np$$ (48)

In which


$$WM1_{l}^{g}=\delta \times \frac{W_{1}\left(x_{a1}-x_{a2}\right)+W_{2}\left(x_{a1}-x_{a3}\right)+W_{3}\left(x_{a2}-x_{a3}\right)}{W_{1}+W_{2}+W_{3}+\varepsilon }+\varepsilon \times rand,\;\;\;\;\;\;\;\;l=1,2,.....,Np$$ (49)

$$WM2_{l}^{g}=\delta \times \frac{\lambda_{1}\left(x_{bs}-x_{bt}\right)+\lambda_{2}\left(x_{bs}-x_{bt}\right)+\lambda_{3}\left(x_{bt}-x_{ws}\right)}{\lambda_{1}+\lambda_{2}+\lambda_{3}+\varepsilon }+\varepsilon \times rand,\;\;\;\;\;\;\;\;\;\;l=1,2,.....,Np$$ (50)

where *W*_1_, *W*_2,_ and *W*_3_ can be calculated as follows:


$$W_{1}=cos\left(\left(f\left(x_{a1}\right)-f\left(x_{a2}\right)\right)+\pi \right)\times exp\left(-\frac{f\left(x_{a1}\right)-f\left(x_{a2}\right)}{\omega }\right)$$ (51)

$$W_{2}=cos\left(\left(f\left(x_{a1}\right)-f\left(x_{a3}\right)\right)+\pi \right)\times exp\left(-\frac{f\left(x_{a1}\right)-f\left(x_{a3}\right)}{\omega }\right)$$ (52)

$$W_{3}=cos\left(\left(f\left(x_{a2}\right)-f\left(x_{a3}\right)\right)+\pi \right)\times exp\left(-\frac{f\left(x_{a2}\right)-f\left(x_{a3}\right)}{\omega }\right)$$ (53)

$$\omega =\max\left(f\left(x_{a1}\right),f\left(x_{a2}\right),f\left(x_{a3}\right)\right)$$ (54)

$$\lambda_{1}=cos\left(\left(f\left(x_{bs}\right)-f\left(x_{bt}\right)\right)+\pi \right)\times exp\left(-\frac{f\left(x_{bs}\right)-f\left(x_{bt}\right)}{\omega }\right)$$ (55)

$$\lambda_{2}=cos\left(\left(f\left(x_{bs}\right)-f\left(x_{ws}\right)\right)+\pi \right)\times exp\left(-\frac{f\left(x_{bs}\right)-f\left(x_{ws}\right)}{\omega }\right)$$ (56)

$$\lambda_{3}=cos\left(\left(f\left(x_{bt}\right)-f\left(x_{ws}\right)\right)+\pi \right)\times exp\left(-\frac{f\left(x_{bt}\right)-f\left(x_{ws}\right)}{\omega }\right)$$ (57)

$$\omega =f\left(x_{ws}\right)$$ (58)

In this context, *f(x)* represents the objective function, while *a*_1_, *a*_2_, and *a*_3_ are randomly selected integers within the range [1, *Np*]. The parameter ε denotes a small positive constant. The variables *x*_*bs*_, *x*_*bt*_, and *x*_*ws*_ refer to the best, better, and worst solutions, respectively, within the *g*^*th*^ generation.

The scale factor *δ,* as indicated in equation (59), modulates the influence of the weighted vector. Furthermore, the parameter *β* is a dynamic variable that evolves according to an exponential function, as outlined in equation (60).


δ=2β×rand−β (59)

$$\beta =2exp\left(-4\times \frac{g}{Maxg}\right)$$ (60)

*Maxg* represents the maximum number of iterations. Within the INFO algorithm, a convergence acceleration (CA) parameter is utilized to enhance global search performance by more effectively directing the population toward promising areas of the solution space. The CA parameter is mathematically defined as follows:


$$CA=randn\times \frac{\left(x_{bs}-x_{a1}\right)}{\left(f\left(x_{bs}\right)-f\left(x_{a1}\right)+\varepsilon \right)}$$ (61)

Here, *randn* denotes a random variable drawn from a standard normal distribution. The newly generated vector is defined as follows:


$$z_{l}^{g}=x_{l}^{g}+\sigma \times MeanRule+CA$$ (62)

The proposed update mechanism, which incorporates the variables *x*_*bs*_*, x*_*bt*_*, x*_*l*_^*g*^, and *x*_*a*1_^*g*^, is formulated according to the following scheme:


if rand<0.5

$$z_{\text{1}l}^{g}=x_{l}^{g}+\sigma \times MeanRule+randn\times \frac{\left(x_{bs}-x_{a\text{1}}^{g}\right)}{\left(f\left(x_{bs}\right)-f\left(x_{a\text{1}}^{g}\right)+\text{1}\right)}$$

$$z_{\text{2}l}^{g}=x_{bs}+\sigma \times MeanRule+randn\times \frac{\left(x_{a\text{1}}^{g}-x_{a\text{2}}^{g}\right)}{\left(f\left(x_{a\text{1}}^{g}\right)-f\left(x_{a\text{2}}^{g}\right)+\text{1}\right)}$$

else

$$z_{\text{1}l}^{g}=x_{a}^{g}+\sigma \times MeanRule+randn\times \frac{\left(x_{a\text{2}}^{g}-x_{a\text{3}}^{g}\right)}{\left(f\left(x_{a\text{2}}^{g}\right)-f\left(x_{a\text{3}}^{g}\right)+\text{1}\right)}$$

$$z_{\text{2}l}^{g}=x_{bt}+\sigma \times MeanRule+randn\times \frac{\left(x_{a\text{1}}^{g}-x_{a\text{2}}^{g}\right)}{\left(f\left(x_{a\text{1}}^{g}\right)-f\left(x_{a\text{2}}^{g}\right)+\text{1}\right)}$$

end (63)

In this formulation, *z*_1*l*_^*g*^ and *z*_2*l*_^*g*^ represent the newly generated vectors for the *g*^*th*^ generation, with *σ* indicating the vector scaling factor, as described in equation (64). It is worth noting that the parameter *α* within equation (64) can be dynamically adjusted in accordance with the exponential function defined in equation (65).


σ=2α×rand−α (64)

$$\alpha =cexp\left(-d\times \frac{g}{Maxg}\right)$$ (65)

The parameters *c* and *d* represent constant values, which are explicitly defined as 2 and 4 in this context.

iii. ***Vector combining stage***

To enhance population diversity, the two vectors computed in the previous section (*z*_1*l*_^*g*^ and *z*_2*l*_^*g*^) are combined with vector *x*_*l*_^*g*^ when the condition ***rand* < 0.5** is met. This process generates the new vector *u*_*l*_^*g*^ as described in equations (66–68). This operator encourages local search, resulting in the creation of a new vector.

***if*** *rand < 0.5*

***if*** *rand < 0.5*


$$u_{l}^{g}=z_{1l}^{g}+\mu .\left|z_{1l}^{g}-z_{2l}^{g}\right|$$ (66)

**else**


$$u_{l}^{g}=z_{2l}^{g}+\mu .\left|z_{1l}^{g}-z_{2l}^{g}\right|$$ (67)

**end**

**else**


$$u_{l}^{g}=x_{l}^{g}$$ (68)

**end**

The combined vector *u*_*l*_^*g*^ is generated in the *g*^*th*^ iteration through vector fusion, where the scaling factor *μ* is determined stochastically as 0.05 multiplied by a uniformly distributed random variable (*μ* = 0.05 × *randn*).

iv. ***Local search stage***

A local search operator has been introduced to enhance the local search capabilities of the INFO algorithm and to prevent convergence to suboptimal solutions. This operator utilizes the global best position (*x*^*g*^_*best*_) along with a mean-based weighting mechanism, as detailed in equations (69–72). When the condition *r* < 0.5 is satisfied (where *r* is a uniformly distributed random variable within the interval [0,1]), the operator generates a new solution vector in the vicinity of *x*^*g*^_*best*_.

***if*** *rand < 0.5*

***if*** *rand < 0.5*


$$u_{l}^{g}=x_{bs}+randn\times \left(MenRule+randn\times \left(x_{bs}^{g}+x_{a1}^{g}\right)\right)$$ (69)

**else**


$$u_{l}^{g}=x_{md}+randn\times \left(MenRule+randn\times \left(v_{1}\times x_{bs}-v_{2}\times x_{md}\right)\right)$$ (70)

**end**

**end**

in which


$$x_{md}=\phi \times x_{avg}+\left(1-\phi \right)\times \left(\phi \times x_{bt}+\left(1-\phi \right)\times x_{bs}\right)$$ (71)

$$x_{avg}=\frac{\left(x_{a}+x_{b}+x_{3}\right)}{\text{3}}$$ (72)

*ϕ* denotes a randomly generated number within the interval [0, 1]. The term *x*_*md*_ refers to a candidate solution created by a random combination of *x*_*avg*_, *x*_*bt*_, and *x*_*bs*_, which enhances the stochastic characteristics of the algorithm and encourages a more extensive exploration of the solution space. Furthermore, the random variables *v*_1_ and *v*_2_ are defined as follows:


$$\left\{x_{i}^{\text{0}},\ldots \ldots ,x_{Np}^{\text{0}}\right\}$$ (73)

**Fig 1:**
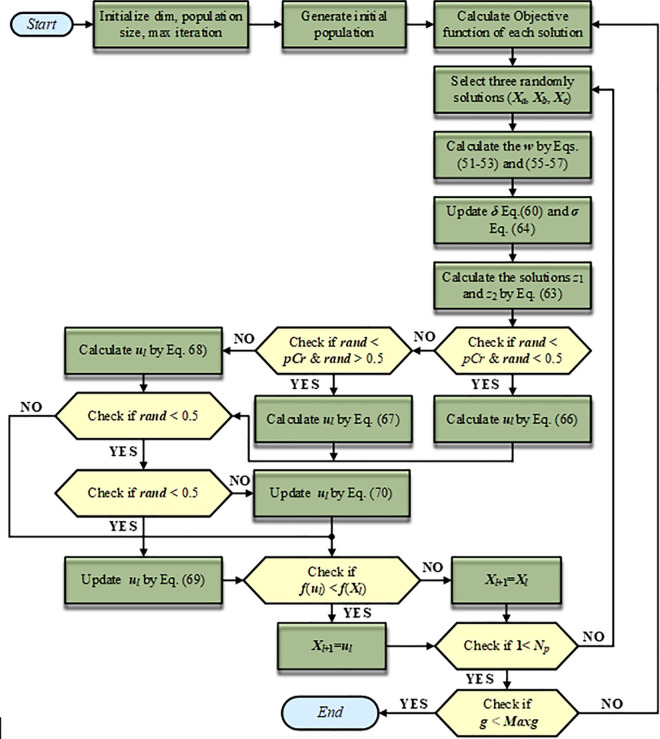
The flowchart of the INFO algorithm.

In this context, *p* represents a randomly selected value within the interval [0, 1]. The random variables *v*_1_ and *v*_2_ may enhance the impact of the best-known position within the solution vector. In summary, the comprehensive structure of the proposed INFO algorithm is outlined in **Algorithm 1**, and its procedural flow is visually illustrated in Fig 1 [2].

**Algorithm 1.** The Pseudo-code of the INFO algorithm [38]

1: **STEP 1. Initialization**

2:  Set parameters *Np* and *Maxg*

3:   Produce an initial population *P*^0^ = $\left\{x_{i}^{\text{0}},\ldots \ldots ,x_{Np}^{\text{0}}\right\}$

4:  Calculate the objective function value of each vector *f*( $x_{i}^{\text{0}}$), *i* = 1, ..., *Np*

5:  Determine the optimal vector *x*_*bs*_

6: **STEP 2. for** *g* = 1 to *Maxg* **do**

7:     **for** *i* = 1 to *Np* **do**

      Select randomly *a* ≠ *b* ≠ *c* ≠ *i* inside the range [1, *Np*]

   **► Updating rule stage**

8:      Calculate the vectors ${z\text{1}}_{i}^{g}$ and ${z\text{2}}_{i}^{g}$ by equation (63)

   **► Vector combining stage**

9:        Compute the vector $u_{i}^{g}$using equations (66-68)

   **► Local search stage**

10:      Compute the local search operator using equations (69-73)

11:        Compute the objective function value *f*($u_{i,j}^{g}$)

12:       **if** $f\left(u_{i,j}^{g}\right)<f\left(x_{i,j}^{g}\right)$ **then** $x_{i,j}^{g+1}=u_{i,j}^{g}$

13:        **Otherwise** $x_{i,j}^{g+1}=x_{i,j}^{g}$

14:       **end for**

15:       Update the optimal vector (*x*_*bs*_)

16:       **end for**

17: **STEP 3. Return** Vector $x_{best,j}^{g}$ as the final solution.

### 5.2. Overview of the Artificial Rabbits Optimization (ARO)

The original ARO mimics the foraging and hiding tactics of actual rabbits, as well as their energy shrink, leading to transiting between these tactics [25].

a) **Detour foraging (exploration)**

In detour foraging behavior of ARO, each individual in the search space tends to update its location towards the other search individual chosen randomly from the group and add a perturbation. The following equation describe the mathematical model of the detour foraging:


$$v_{i}\left(t+1\right)=x_{j}\left(t\right)+R\times \left(x_{i}\left(t\right)-x_{j}\left(t\right)\right)+round\left(0.5\times \left(0.05+r_{1}\right)\right)\times n_{1},\;\;\;i,j=1,\ldots .,j\neq i$$ (74.1)

R=L×c (74.2)

$$L=\left(e-e^{{\left(\frac{t-1}{T}\right)}^{2}}\right)\times \sin\left(2\pi r_{2}\right)$$ (74.3)

$$c\left(k\right)=\{\begin{array}{c} {}*20c1\;\;\;if\;k==g\left(l\right)\\ 0\;\;\;\;else\;\;\;\; \end{array}\;k=1,\ldots .,d\;and\;l=1,\ldots .,\left[r_{3},d\right]$$ (74.4)

$$g=randperm\left(d\right),\;n_{1}\sim N\left(0,1\right)$$ (74.5)

b) **Random hiding (exploitation)**

To escape from predators, a rabbit commonly digs some holes nearby its nest for hiding. This equation is given in this regard as:


$$b_{i,j}\left(t\right)=x_{i}\left(t\right)+H.g.x_{i}\left(t\right),\;i=1,\ldots .,n\;and\;j=1,\ldots .,d$$ (75.1)

$$H=\frac{T-t+1}{T}.r_{4},\;n_{2}\sim N\left(0,1\right)$$ (75.2)

$$g\left(k\right)=\{\begin{array}{c} {}*20c1\;\;\;if\;k==j\\ 0\;\;\;\;else\;\;\;\; \end{array}\;k=1,\ldots .,d\;$$ (75.3)

The rabbits to be survive need to find a safe residence to hide. So, they select randomly a hole from their holes for hiding to escape from getting caught. this random hiding tactic is modeled as below:


$$v_{i}\left(t+1\right)=x_{i}\left(t\right)+R\times \left(r_{4}\times b_{ir}\left(t\right)-x_{i}\left(t\right)\right)\;\;\;i=1,\ldots .,n$$ (76.1)

$$g\left(k\right)=\{\begin{array}{c} {}*20c1\;\;\;if\;k==\left[r_{5}\times d\right]\\ 0\;\;\;\;else\;\;\;\; \end{array}\;k=1,\ldots .,d\;$$ (76.2)

$$b_{i,r}\left(t\right)=x_{i}\left(t\right)+H.g.x_{i}\left(t\right)$$ (76.3)

After detour foraging or p random hiding is reached, the position update of the *i*^th^ rabbit is:


$$x_{i}\left(t+1\right)=\{\begin{array}{c} {}*20cx_{i}\left(t\right)\;\;\;\;f(x_{i}\left(t\right)\;\leq f\left(v_{i}\left(t+1\right)\;\right)\\ v_{i}\left(t+1\right)\;\;\;\;f(x_{i}\left(t\right)\;>f\left(v_{i}\left(t+1\right)\;\right)\;\;\; \end{array}\;\;$$ (77)

c) **Energy shrink (switch from exploration to exploitation)**

An energy factor is considered to model the switch from exploration to exploitation phases. The energy factor in this algorithm can be given as follows:


$$A\left(t\right)=4\left(1-\frac{t}{T}\right)\ln\frac{\text{1}}{r}$$ (78)

where, g is the index of the best solution.

### 5.2. Modified ARINFO Algorithm

**Fig 2:**
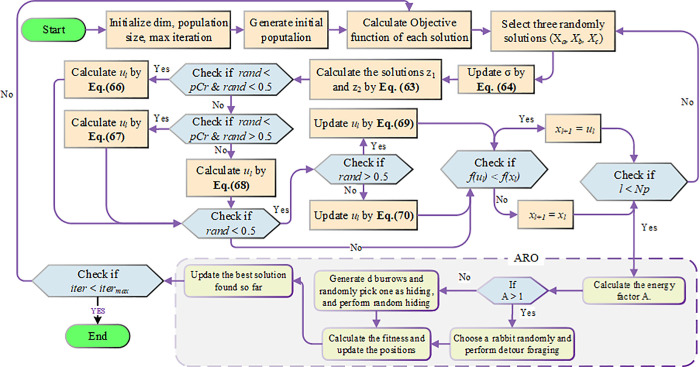
Flowchart of the ARINFO algorithm.

The flowchart of the proposed ARINFO algorithm is presented in Fig 2. Moreover, Algorithm 2 describes the ARINFO algorithm’s pseudocode. The proposed ARINFO algorithm offers several advantages in addressing complex optimization problems. Its primary strength lies in achieving an effective exploration-exploitation balance, which enhances search efficiency and allows the algorithm to navigate diverse solution spaces while converging on optimal solutions. By integrating ARO with INFO, ARINFO demonstrates improved robustness and adaptability across various problem domains. Additionally, it can efficiently handle high-dimensional datasets, making it suitable for real-world applications requiring precise optimization. However, ARINFO may face challenges due to its computational complexity, which can increase with larger datasets or intricate problem structures, potentially slowing convergence.

**Algorithm 2**: Pseudocode of the ARINFO algorithm

*Set the control parameter (Dimension of problem (d), maximum iteration, population size), and h*

 *Initialize the population randomly*

 *Evaluate the fitness of the new solution*

 *Obtain the best solution*

  *While t ≤ T*

    *%INFO*

     *for i = 1: Np*

      *Select randomly a ≠ b ≠ c ≠ i inside the range [1, Np]*

     *Updating rule stage*

      *Calculate the vectors* $z1_{i}^{g}$ *and* $z2_{i}^{g}$ *by equation (63)*

     *Vector combining stage*

      *Compute the vector* $u_{i}^{g}$ *using equations (66–68)*

     *Local search stage*

      *Compute the local search operator using equations (69–73)*

      *Compute the objective function value*

      *Update the optimal vector (xbs)*

     *end for*

     *%ARO*

     *Calculate the energy factor A*

     *If* A>0.5

       *Choose a rabbit randomly and perform detour foraging*

     *Else If* A≤0.5

       *Generate burrows and randomly pick one as hiding and perform random hiding*

     *End If*

     *Check the limits of the new locations and evaluate the fitness values*

     *Find the new solution if the fitness is better*

     *t = t + 1*

   *End while*

   *Output the best solution*

## 6. Simulation results and discussion

The OPF problem is addressed through the proposed ARINFO algorithm. To evaluate its performance and computational efficiency, ARINFO is tested on the benchmark suites CEC-2017 and CEC-2022 [43–45]. The results are then compared with several established optimization algorithms, including INFO, AHA, ARO, WOA, SWO, PSO, MFO, SOA, and SCA. To ensure consistency and fairness in the comparisons, all competing algorithms were configured using the parameter settings outlined in their original publications. ARINFO was implemented in MATLAB 2024a and executed on a system equipped with an Intel Core i7 processor (2.10 GHz) and 32 GB of RAM. Detailed numerical results are presented in the following sections.

a. **Exploration vs Exploitation**

**Fig 3:**
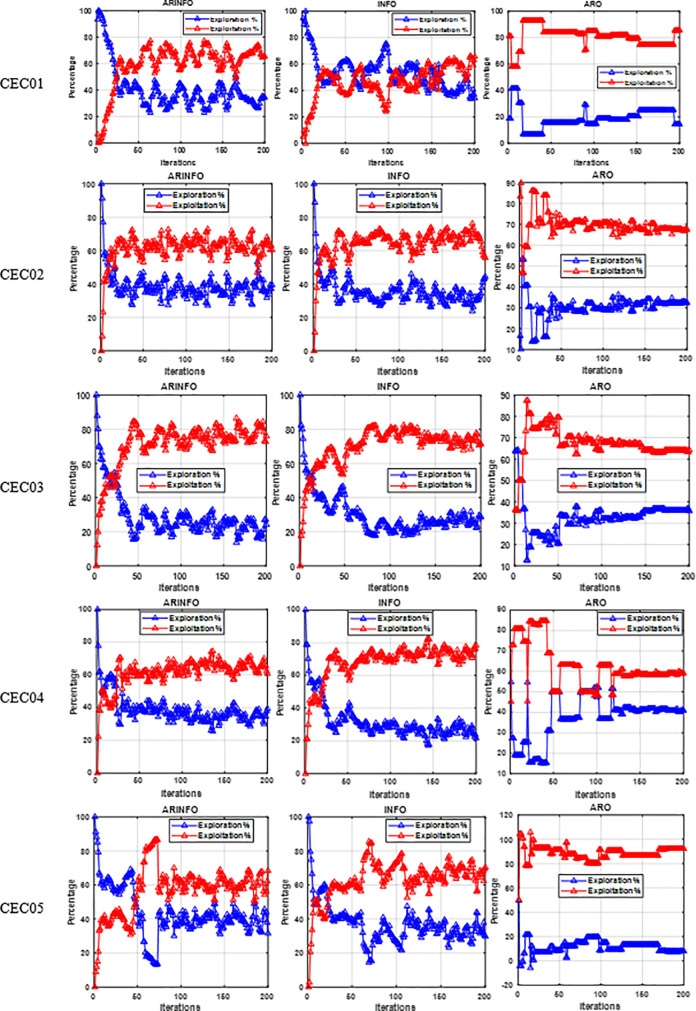
Exploration vs exploitation analysis of ARINFO, INFO, and ARO algorithms.

Examining the balance between the exploration and exploitation phases of the ARINFO can provide significant insights for tackling practical optimization issues. To facilitate a comprehensive assessment of the ARINFO, this research investigates the algorithm’s capabilities in exploration and exploitation, as informed by the five CEC-2022 benchmark functions analyzed. Fig 3 depicts the equilibrium between the exploratory and exploitative characteristics of the ARINFO, INFO, and ARO techniques. In this figure, the red curve denotes the algorithm’s exploratory inclinations, while the blue curve emphasizes its exploitative capabilities. From this figure, the proposed ARINFO demonstrates a better balance between exploration and exploitation, which typically leads to stronger global search ability and improved optimization performance.

b. **Qualitative Analysis**

Qualitative analysis serves to evaluate the robustness of the proposed ARINFO algorithm by examining the behavior of search agents and the evolution of the objective function throughout the optimization process. Fig. B1 (Appendix B) in S1 File illustrates qualitative outcomes for a representative set of test functions: three unimodal functions (F1, F4, and F7), three multimodal functions (F8, F10, and F12), and three composite functions (F14, F18, and F21) [46].

In the first column of Fi B1, a three-dimensional visualization of each fitness landscape provides insights into the shape and complexity of the objective function. The second column presents the progression of search agents across iterations, demonstrating that ARINFO effectively guides the population toward regions of lower objective values. The third and fourth columns, respectively, depict the average fitness values over time and the convergence trajectory of the algorithm. Analysis of these plots reveals significant fluctuations in the early stages of the search, indicative of extensive exploration. These fluctuations diminish later, reflecting a transition to exploitation and convergence toward optimal or near-optimal solutions. This observed behavior confirms the algorithm’s robustness and capacity to balance exploration and exploitation effectively.

c. **Statistical Analysis**

The statistical performance of the proposed ARINFO algorithm, alongside INFO [23], AHA [24], ARO [25], WOA [26], SWO [27], PSO [28], MFO [29], SOA [30], and SCA [31], is presented in Tables B1 and B3 (Appendix B) in S1 File for the CEC 2017 and CEC 2022 benchmark functions, respectively. In these tables, the best-performing results are highlighted in bold red for clarity. Based on the results shown in Table B1 (Appendix B) in S1 File, ARINFO outperforms the competing algorithms on several benchmark functions, including F1 to F4, F6, F12, and F15 to F23. Its performance is comparable to other leading algorithms on functions F9 to F11 and F14. However, ARINFO performs comparatively less on functions F5, F7, F8, and F13. The average rank and overall ranking for the CEC 2017 benchmarks are summarized in Table B2 in S1 File, which confirms the superior performance of ARINFO relative to the other optimization techniques.

Similarly, the statistical analysis in Table B3 in S1 File demonstrates ARINFO’s leading performance on most functions of the CEC 2022 suite, specifically CEC01, CEC03 to CEC07, and CEC10 to CEC11. For CEC08, ARINFO performs on par with competing algorithms, while it outperforms CEC02, CEC09, and CEC12. The corresponding average rank and overall rankings for the CEC 2022 benchmark functions are provided in Table B4, further underscoring ARINFO’s strong comparative performance.

d. **Convergence Behavior**

The convergence trends for the benchmark functions CEC-2017 and CEC-2022 are depicted in Figs B2 and B3 (Appendix B) in S1 File, respectively. As illustrated in these figures, the proposed ARINFO algorithm demonstrates superior convergence characteristics for the majority of test functions, achieving faster convergence rates compared to other algorithms such as INFO, AHA, ARO, WOA, SWO, PSO, MFO, SOA, and SCA.

However, there are specific instances, such as with functions F7, F8, and F13, where algorithms like SOA and ARO exhibit more favorable convergence dynamics. Additionally, PSO performs best for function CEC09. Despite these exceptions, ARINFO consistently converges more efficiently in most scenarios, requiring fewer iterations to attain optimal or near-optimal solutions. These findings underscore the robustness and effectiveness of the algorithm in addressing a diverse array of complex optimization problems.

e. **Boxplot Analysis**

The boxplot representations comparing the ARINFO algorithm with competing methods on the CEC-2017 and CEC-2022 benchmark functions are illustrated in Figs B4 and B5 (Appendix B). In these plots, the whiskers represent the minimum and maximum values achieved by each algorithm. A narrower box indicates greater consistency and robustness in the results. The ARINFO algorithm exhibits remarkable statistical stability, with no outliers detected across more than thirteen functions within the CEC-2017 suite, including F1, F2, F3, F4, F9, F10, F11, F14, F15, F17, F18, F22, and F23. Similarly, it shows strong performance on key functions of the CEC-2022 suite, particularly CEC01, CEC05, CEC09, and CEC10.

Overall, the boxplot distributions reveal that ARINFO consistently achieves lower objective values and demonstrates tighter performance variability compared to its competitors. This confirms its superior reliability and effectiveness in addressing complex optimization challenges.

f. **p-Value-Based Statistical Analysis**

The statistical validity of the performance differences between the proposed ARINFO algorithm and its competitors is evaluated through the Wilcoxon rank-sum test. This non-parametric test facilitates pairwise comparisons between optimization methods by analyzing their result distributions. A significant difference is indicated by a p-value below 0.05, while a p-value exceeding this threshold suggests no statistically significant distinction. Table B5 (Appendix B) in S1 File displays the p-values for comparisons between ARINFO and various algorithms (INFO, AHA, ARO, WOA, SWO, PSO, MFO, SOA, and SCA) on the CEC-2017 benchmark functions. The majority of these p-values are below 0.05, indicating substantial performance differences in favor of ARINFO. Instances where p-values exceed 0.05 are highlighted in bold, signifying that in those cases, ARINFO’s performance is statistically comparable to that of other methods.

Likewise, Table B6 (Appendix B) in S1 File presents the p-values for comparisons on the CEC-2022 suite. Once again, most values fall below the significance threshold, affirming ARINFO’s statistically superior results in the majority of benchmark scenarios. Bold entries indicate non-significant differences (p-value > 0.05). Additionally, “NaN” values reflect identical outcomes between ARINFO and another algorithm, rendering statistical comparison unfeasible.

Furthermore, the results from the benchmark functions CEC-2017 and CEC-2022, as outlined in Tables B7 and B8 (Appendix B) in S1 File, were subjected to statistical analysis using ANOVA, the Friedman test, and the Kruskal-Wallis test. The findings consistently demonstrate that the proposed ARINFO algorithm outperforms the nine alternative optimization methods, as indicated by p-values below the 0.05 significance threshold, thereby affirming its superior effectiveness.

## 7. Application of ARINFO to the Optimal Power Flow Problem

This section provides a thorough evaluation of the performance of the ARINFO algorithm in solving the OPF problem. It compares ARINFO to several established metaheuristic algorithms, including ARO, AHA, PSO, SOA, MFO, and the standard INFO. The analysis utilizes multiple realistic case studies to validate the effectiveness and robustness of ARINFO in tackling OPF challenges.

The IEEE 30-bus test system has been modified to integrate renewable energy sources. Specifically, the thermal generators at buses 5 and 11 have been replaced with wind power generators, while the thermal unit at bus 13 has been substituted with a solar photovoltaic (PV) system. A similar modification has been applied to the IEEE 57-bus system, where thermal generators at buses 2 and 6 are now wind farms, and the generator at bus 9 has been transformed into a solar PV plant. Detailed specifications of the integrated wind and solar units for both systems are outlined in Table A2 in Appendix A in S1 File and Table 1.

**Table 1:** Wind and solar plants data- IEEE −57 bus system [23].

Wind plants				
**Wind plant**	**No. of wind Turbines**	**Rated power**	**Weibull Parameters**	**Weibull mean,** $M_{weibull}$
1	25	100 MW	k = 2, c = 9	$Wd_{v}$ = 7.976 (m/s)
2	20	100 MW	k = 2, c = 10	$Wd_{v}$ = 8.862 (m/s)
Solar PV plants				
**Solar plant**	**Rated power**		**Lognormal parameters**	**Lognormal mean,** $M_{lgn}$
1	100 MW		*δ* = 0.6, *µ* = 6	I = 483 (W/m^2^)

In the IEEE 30-bus system, the control variables comprise the scheduled active power outputs of all thermal generators (excluding the slack bus at bus 1), as well as the scheduled outputs of the two wind farms and the solar PV plant. Additionally, the voltage magnitudes at all generator buses are included as control parameters. In the configuration of the IEEE 57-bus system, the set of control variables is expanded to encompass the reactive power injections from shunt compensators and the tap positions of transformer branches. Case studies 1–8 are based on the modified IEEE 30-bus system, while Cases 9 and 10 focus on the IEEE 57-bus system. All simulation experiments are conducted using MATLAB, ensuring a realistic modeling of system dynamics and optimization under practical constraints.

### 7.1. Case 1: Minimizing the Total Cost

This section addresses the optimization problem of the modified IEEE 30-bus power system, focusing on minimizing the total generation cost while adhering to system constraints outlined in equation (42). The ARINFO algorithm achieves this goal with a termination criterion of 300 maximum iterations per run across 30 independent trials. The statistical results indicate that ARINFO consistently produces outcomes close to the optimal solution. The recorded total cost values ranged from a best of 781.1538$/h to a worst of 782.0832$/h, yielding a mean value of 781.8054$/h and a standard deviation of 0.1553. These findings reflect both the high quality and robustness of the solutions. The average computational time per run was approximately 581.4 sec.

**Fig 4:**
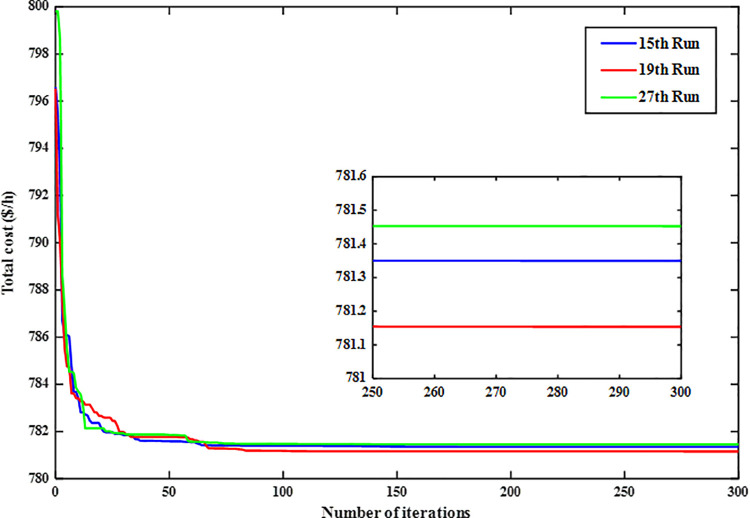
Convergence curves of the ARINFO algorithm for the three best-performing runs.

For further analysis, three representative runs, the 15th, 19th, and 27th, were selected. As illustrated in Fig 4, the corresponding generation costs for these runs were 781.3501, 781.1538, and 781.4528$/h, respectively, demonstrating the algorithm’s ability to achieve near-optimal solutions consistently.

**Fig 5:**
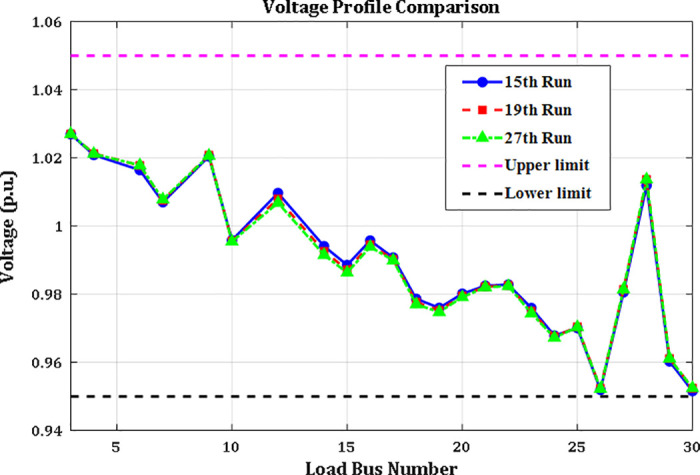
Voltage profile of load buses.

**Fig 6:**
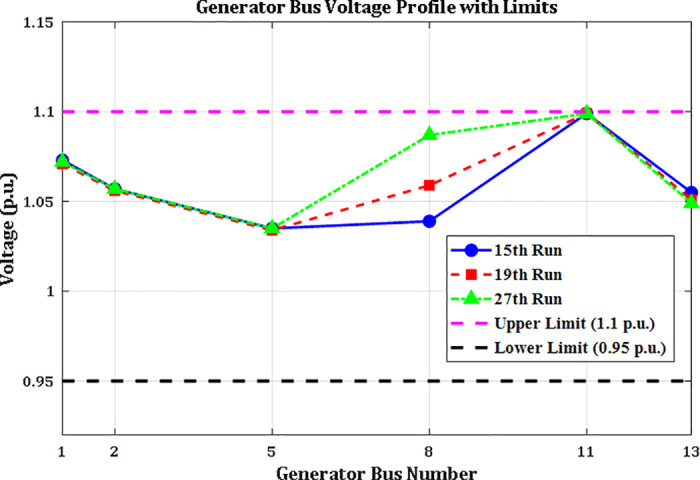
Voltage profiles of generator buses.

A crucial component of the OPF analysis is maintaining voltage levels at load buses within acceptable operational limits. In the context of the IEEE 30-bus system, these voltages must be constrained within the range of 0.95 to 1.05(p.u.). Proper regulation of load bus voltages is essential to ensure system stability and reliability. Fig 5 illustrates the voltage profiles of the load buses across the initial three iterations utilizing the proposed INFO algorithm. As shown in Table 2 and Fig 6, the voltages at all load buses remain comfortably within the established safety margins. Furthermore, the voltage levels at the generator buses are consistently maintained within their specified upper and lower thresholds.

**Table 2:** Simulation results for Case 1.

Control Variables	Min	Max	ARINFO	INFO	AHA	ARO	PSO	SOA	MFO
PTG1 (MW)	50	140	134.8572	134.6245	134.8033	134.8012	134.8443	134.8901	134.2371
PTG2 (MW)	20	80	29.0988	26.8281	27.7088	27.5282	28.3859	26.97439	29.28086
PTG3 (MW)	10	35	10	10	10	10	10	10.0275	10.00063
PwG1 (MW)	0	75	44.1472	42.8761	43.2687	43.3343	43.8938	42.8372	44.18373
PwG2 (MW)	0	60	37.2741	36.2176	36.5685	36.49172	36.46538	35.89824	37.19143
PsG1 (MW)	0	50	33.73226	38.3695	36.7347	36.9236	35.52811	38.6623	33.60172
V1 (p.u)	0.95	1.1	1.0731	1.0708	1.0714	1.0715	1.0707	1.0723	1.0719
V2 (p.u)	0.95	1.1	1.0574	1.0559	1.0562	1.0565	1.0558	1.0237	1.0568
V5 (p.u)	0.95	1.1	1.0349	1.0342	1.0341	1.0347	1.0333	1.0356	1.0342
V8 (p.u)	0.95	1.1	1.0390	1.0699	1.0884	1.0990	1.0784	1.0859	1.0658
V11 (p.u)	0.95	1.1	1.0987	1.0995	1.0999	1.0998	1.1	1.0971	1.0982
V13 (p.u)	0.95	1.1	1.0551	1.0519	1.0505	1.0486	1.0558	1.0913	1.0493
**Parameters**	**Min**	**Max**	**ARINFO**	**INFO**	**AHA**	**ARO**	**PSO**	**SOA**	**MFO**
QTG1 (MVAr)	−20	150	−0.13806	−2.90218	−2.0883	−2.40613	−3.026058	15.3182	−1.73967
QTG2 (MVAr)	−20	60	12.5474	11.14042	11.1486	11.7110	11.10630	−20	11.6577
QTG3 (MVAr)	−15	40	34.0696	40	40	40	40	40	40
QwG1 (MVAr)	−30	35	22.8410	22.6544	22.30185	22.71166	21.3460	28.6406	21.77943
QwG2 (MVAr)	−25	30	30	30	30	30	30	28.4790	30
QsG1 (MVAr)	−20	25	17.6694	16.1840	15.6859	15.0111	17.54696	25	15.30442
Total cost ($/h)			**781.1538**	781.7091	781.9313	781.8087	782.31295	782.84853	781.8317
Emissions (t/h)			1.76194	1.762501	1.76227	1.76229	1.761845	1.76246	1.761992
P_loss_ (MW)			5.76044	5.79943	5.78876	5.78571	5.77881	5.90767	5.767172
V_d_ (p.u)			0.46245	0.46125	0.457199	0.453905	0.47022	0.53065	0.45429
Computational time (sec)			581.4	685.5	735	658.78	766	698.34	629.5

Case 1 focuses on minimizing the total generation cost across all power sources within the modified IEEE 30-bus power system, as defined by equation (42). The parameters of the PDF utilized in this scenario are outlined in Table 2, while the relevant cost coefficients are detailed in Table A1 in Appendix A. The optimization results obtained using the proposed ARINFO algorithm are presented in Table 2, alongside comparisons with other optimization methods such as AHA, ARO, PSO, SOA, MFO, and the standard INFO. The control variables for this case include the voltage magnitudes at all generator buses and the scheduled active power outputs of all generators except TPG1, which acts as the slack bus. The operational limits for these control variables conform to the specifications provided in [42]. In addition to the objective function values shown in Table 2, the total voltage deviation across the load buses, calculated using equation (42), and the overall system power loss determined by equation (45) are also documented.

**Fig 7:**
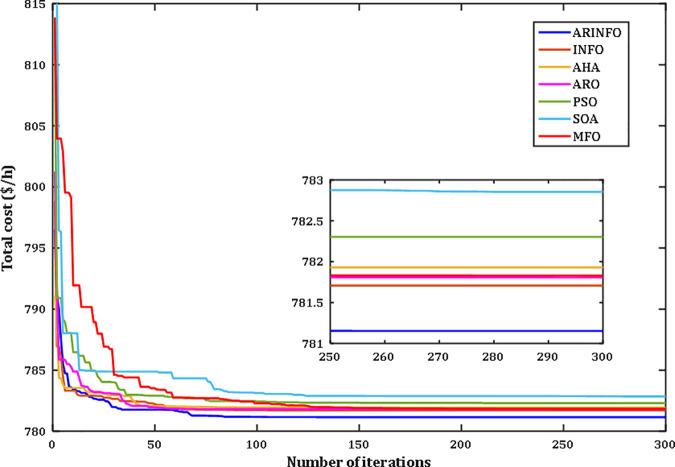
Convergence performance comparison for Case 1.

The results highlight the exceptional performance of the ARINFO algorithm, which achieved the lowest total generation cost of 781.1538$/h, demonstrated the shortest computational runtime of 581.4 sec, and exhibited the fastest convergence rate, as illustrated in Fig 7.

### 7.2 Case 2: Optimization of Total Cost with Varying Reserve Cost Coefficients

In this case, the same system parameters from Case 1 are retained, with the sole exception being the modification of the reserve cost coefficient (RCC) to evaluate its effect on optimal power generation scheduling. To explore the impact of different RCC values, three subcases are examined: Case 2a with RCC = 5, Case 2b with RCC = 6, and Case 2c with RCC = 7. The penalty cost coefficient (PCC) for all RESs remains constant at 1.5, consistent with Case 1 [14].

**Fig 8:**
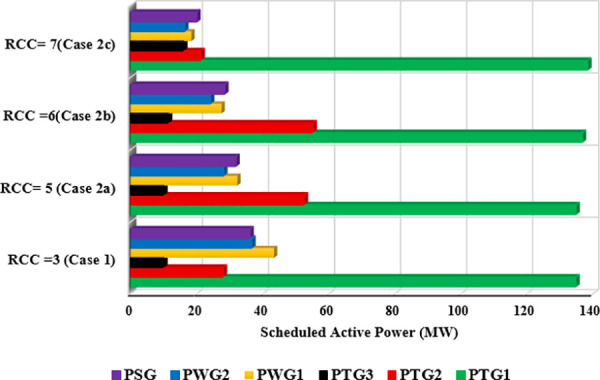
Optimal scheduled active power outputs of all generators under different reserve cost coefficients.

For each subcase, the optimal power output schedule for all generators is established and illustrated in Fig 8. As anticipated, increasing the reserve cost coefficient leads to a decrease in the scheduled output from wind and solar power sources. This outcome highlights the system’s strategy to minimize potential reserve costs linked to power overestimation by reducing dependence on variable renewable generation. Consequently, thermal power units are scheduled to generate more electricity to offset the diminished contribution from RESs.

**Fig 9:**
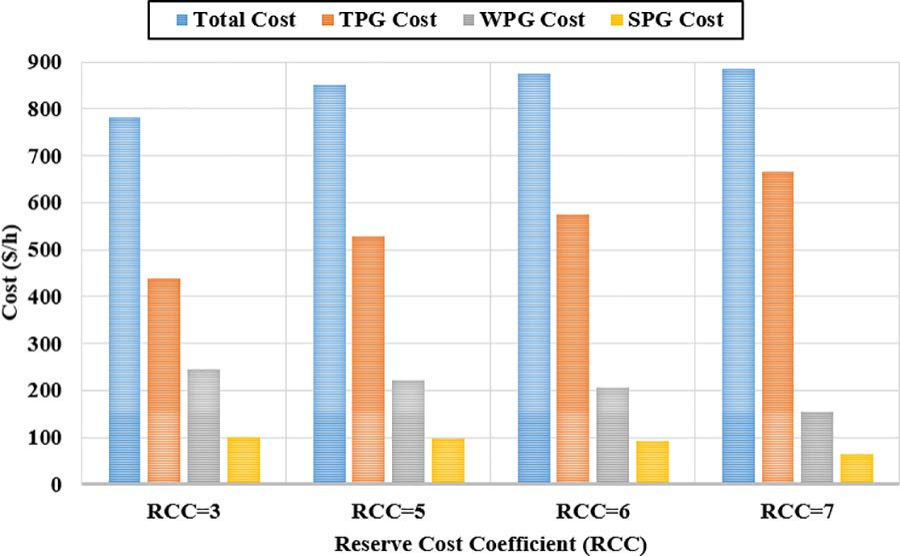
Impact of varying reserve cost coefficients on individual cost components.

While this adjustment alleviates production costs related to renewables, it results in higher costs for thermal generation. Overall, the total generation cost rises with increasing reserve cost coefficients, as depicted in Fig 9.

### 7.3. Case 3: Optimization of Total Cost with Varying Penalty Cost Coefficients

In this case study, all system parameters are consistent with those in Case 1, except the penalty cost coefficients (PCCs). To examine the effects of varying penalty costs, we conducted three subcases applying different PCC values to both wind and solar power sources: Case 3a with PCC = 3, Case 3b with PCC = 4, and Case 3c with PCC = 5. The RCC for all RESs remains constant at 3, aligning with its value in Case 1 [14].

**Fig 10:**
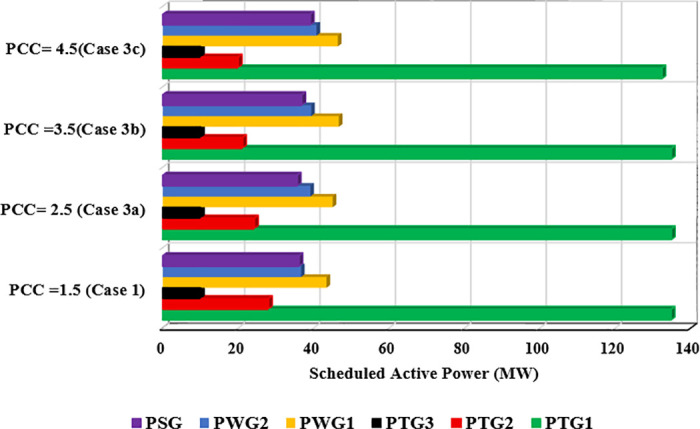
Optimal scheduled active power outputs of all generators under different penalty cost coefficients.

The optimal power schedules for all generators across these subcases are displayed in Fig 10. As anticipated, an increase in the PCC results in a corresponding rise in the scheduled power output from RESs. This adjustment aims to mitigate the risk of incurring high penalties associated with underestimating renewable generation. Consequently, the scheduled output from thermal generators is reduced to accommodate the greater share of renewable energy in the mix.

**Fig 11:**
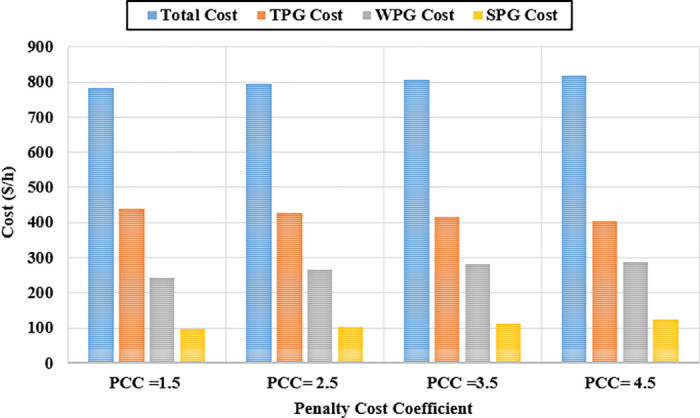
Impact of varying penalty cost coefficients on individual cost components.

These scheduling adjustments have a direct impact on the cost structure, as shown in Fig 11. While production costs for wind and solar generation increase, thermal generation costs decline. Nevertheless, the total generation cost exhibits an upward trend across the subcases due to the heightened reliance on renewables, which results in higher penalty costs.

**Fig 12:**
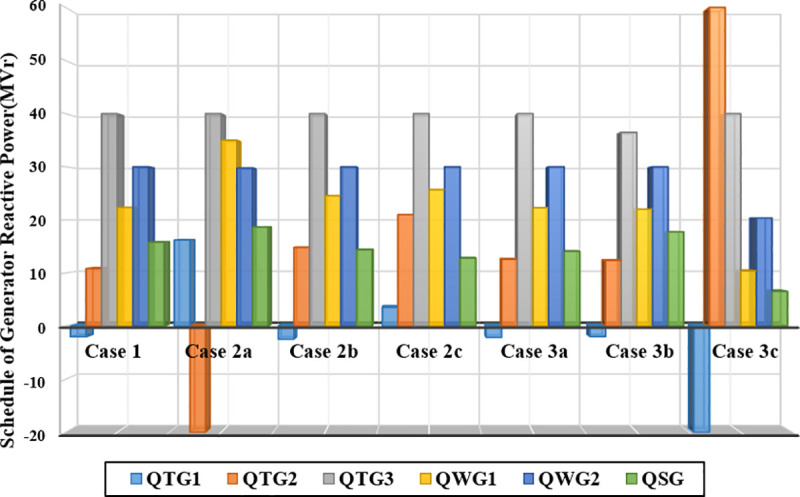
Scheduled reactive power outputs of all generators in Cases 1, 2, and 3.

The reactive power outputs for Cases 1, 2, and 3 are depicted in Fig 12. The data indicate that both TPG3 and WPG2 consistently exceeded their maximum reactive power thresholds. This finding emphasizes the critical importance of incorporating reactive power constraints within the optimization process to maintain system feasibility and ensure reliable performance.

**Fig 13:**
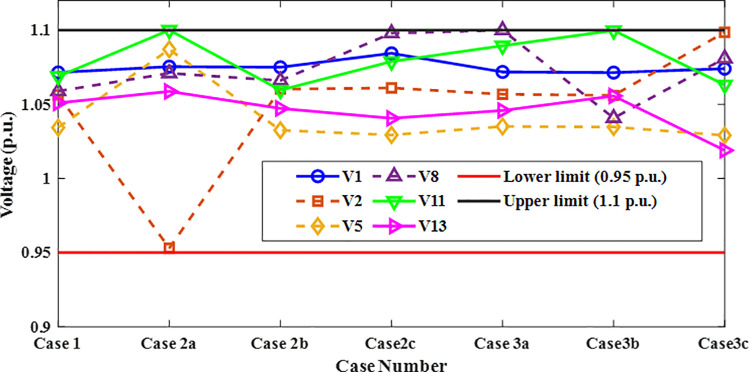
Voltage profiles of generator buses in Cases 1, 2, and 3.

Fig 13 presents the voltage magnitudes at the generator buses for the same scenarios. All observed voltage levels fall within the permissible range of 0.95 to 1.1 per unit, thereby confirming compliance with established voltage standards across all evaluated cases.

### 7.4. Case 4: Minimization of Total Generation Cost with Carbon Tax

In this scenario, the ARINFO algorithm is utilized to assess the impact of implementing a carbon tax on emissions from thermal power generation. The goal is to minimize the total generation cost under a carbon taxation policy, as articulated in equation (43). All system parameters remain consistent with those outlined in Tables 2 and 11, except for the carbon tax rate, which is $20 per tonne of CO₂ emissions.

**Table 11:** Optimal settings of control variables for Case 11 using the ARINFO algorithm.

Variables	ARINFO	INFO	ARO	Variables	ARINFO	INFO	ARO	Variables	ARINFO	INFO	ARO	Variables	ARINFO	INFO	ARO	Variables	ARINFO	INFO	ARO
P_G1_(MW)	39.34687	55.02155	32.98773	P_G65_(MW)	192.9505	245.5075	151.7344	V_G1_(p.u.)	0.974593	0.971103	1.013464	V_G65_(p.u.)	0.971461	0.968519	1.026734	T_8_(8−5)	20.89378	3.774407	9.883548
P_G4_(MW)	31.60777	31.26951	64.13632	P_G66_(MW)	298.2395	230.496	169.5496	V_G4_(p.u.)	1.015109	0.995011	1.021118	V_G66_(p.u.)	1.005875	1.004825	1.023865	T_32_(25–26)	0.23482	23.50689	9.73641
P_G6_(MW)	31.38449	34.58741	37.81265	P_G69_(MW)	363.8434	341.9478	138.596	V_G6_(p.u.)	0.999726	0.989603	1.038834	V_G69_(p.u.)	1.004061	1.040657	1.036863	T_36_(17–30)	2.668548	17.4464	22.65877
P_G8_(MW)	45.95735	30.00076	48.3428	P_G70_(MW)	30.00028	39.82559	51.96468	V_G8_(p.u.)	0.979701	0.980518	1.014587	V_G70_(p.u.)	0.997267	0.985014	1.036174	T_51_(37–38)	1.565573	22.05749	17.59917
P_G10_(MW)	331.8266	391.2087	215.3456	P_G72_(MW)	30.34961	51.43912	35.61365	V_G10_(p.u.)	1.024825	0.989576	0.992069	V_G72_(p.u.)	1.001913	0.974392	1.039049	T_93_(59–63)	0.6093	15.92039	8.745117
P_G12_(MW)	65.94025	55.53006	95.55706	P_G73_(MW)	30.00001	41.81151	34.90255	V_G12_(p.u.)	0.994981	0.981466	1.044049	V_G73_(p.u.)	1.004873	0.973341	1.024303	T_95_(61–64)	7.106872	1.155067	5.532411
P_G15_(MW)	30.95613	30.00436	57.16027	P_G74_(MW)	30.00309	30.89281	61.97827	V_G15_(p.u.)	0.989398	0.976457	1.027632	V_G74_(p.u.)	0.978266	0.966296	1.018336	T_102_(65–66)	0.13675	0.61569	8.994424
P_G18_(MW)	30.00017	36.59592	53.09084	P_G76_(MW)	30.54358	36.45365	54.80643	V_G18_(p.u.)	0.996678	0.975548	1.045492	V_G76_(p.u.)	0.960992	0.950097	1.011489	T_107_(68–69)	21.14947	0.933876	9.672707
P_G19_(MW)	30.07104	70.92665	66.03174	P_G77_(MW)	31.94315	71.314	48.35345	V_G19_(p.u.)	0.989301	0.974599	1.029966	V_G77_(p.u.)	0.993578	0.978327	1.01081	T_127_(80–81)	15.46854	0.381274	17.1465
P_G24_(MW)	30.00272	38.9584	38.29442	P_G80_(MW)	347.8249	332.4242	241.9884	V_G24_(p.u.)	1.005204	0.979284	1.079511	V_G80_(p.u.)	1.016176	0.978813	1.008373	Q_C5_(MVAR)	0.929074	3.049234	9.425382
P_G25_(MW)	153.6052	111.0283	225.5061	P_G85_(MW)	31.38669	68.75994	34.69667	V_G25_(p.u.)	1.022995	0.998868	1.003999	V_G85_(p.u.)	0.98149	0.980678	1.026945	Q_C34_(MVAR)	12.793	8.104086	5.562792
P_G26_(MW)	227.2443	149.1819	224.0452	P_G87_(MW)	31.2	31.2	31.21066	V_G26_(p.u.)	0.974393	0.958932	0.97951	V_G87_(p.u.)	1.005933	0.99089	1.078993	Q_C37_(MVAR)	1.979822	6.620445	11.20362
P_G27_(MW)	38.54889	45.67262	47.90186	P_G89_(MW)	345.8961	228.1954	218.2901	V_G27_(p.u.)	0.989727	0.984425	1.026186	V_G89_(p.u.)	0.995013	0.989488	1.052025	Q_C44_(MVAR)	11.19413	14.42373	11.05027
P_G31_(MW)	32.10038	32.10038	32.21202	P_G90_(MW)	30.00018	30.0073	35.60325	V_G31_(p.u.)	1.000257	0.994735	1.007645	V_G90_(p.u.)	0.979986	0.970385	1.001947	Q_C45_(MVAR)	0.790002	1.119776	3.57897
P_G32_(MW)	30.00025	34.57391	51.48428	P_G91_(MW)	30.49064	31.75993	52.57754	V_G32_(p.u.)	0.99855	0.98739	1.022299	V_G91_(p.u.)	0.981199	0.977812	1.053284	Q_C46_(MVAR)	0.938315	0.941287	1.013136
P_G34_(MW)	38.23584	82.76053	61.77416	P_G92_(MW)	70.16424	33.78254	49.49417	V_G34_(p.u.)	0.99022	0.980607	1.046045	V_G92_(p.u.)	0.989471	0.97805	1.035339	Q_C48_(MVAR)	0.936937	0.947746	1.005333
P_G36_(MW)	30.00003	30.0609	37.1261	P_G99_(MW)	31.62446	44.27737	58.81636	V_G36_(p.u.)	0.986109	0.974084	1.046305	V_G99_(p.u.)	0.994232	0.984967	0.989287	Q_C74_(MVAR)	0.954674	0.944022	1.00894
P_G40_(MW)	30.06673	49.02845	62.7549	P_G100_(MW)	172.8631	156.1756	136.4883	V_G40_(p.u.)	0.980844	0.976631	0.975115	V_G100_(p.u.)	1.000062	0.989065	1.015812	Q_C79_(MVAR)	0.949443	0.997569	0.960027
P_G42_(MW)	30.14518	36.45494	58.88617	P_G103_(MW)	42.35663	42	74.34437	V_G42_(p.u.)	0.988476	0.981858	0.981035	V_G103_(p.u.)	0.999814	0.992245	1.010405	Q_C82_(MVAR)	0.937574	0.952798	1.050909
P_G46_(MW)	35.70452	40.14702	38.0663	P_G104_(MW)	30.42764	37.30969	36.40016	V_G46_(p.u.)	1.001047	0.977774	0.99669	V_G104_(p.u.)	0.986449	0.979918	1.005738	Q_C83_(MVAR)	0.964208	0.950125	0.96312
P_G49_(MW)	159.8352	125.718	143.1335	P_G105_(MW)	30.00305	33.35325	39.39104	V_G49_(p.u.)	1.005762	0.989249	1.053175	V_G105_(p.u.)	0.985947	0.980943	1.004445	Q_C105_(MVAR)	0.953302	0.931835	0.996958
P_G54_(MW)	45.41423	53.80407	68.3421	P_G107_(MW)	30	30.00094	65.34601	V_G54_(p.u.)	0.982364	0.981318	1.020838	V_G107_(p.u.)	0.984531	0.99278	1.020693	Q_C107_(MVAR)	0.931107	0.939971	0.931119
P_G55_(MW)	30.00186	32.08728	67.95739	P_G110_(MW)	31.28731	33.62405	66.64474	V_G55_(p.u.)	0.978744	0.977792	1.018953	V_G110_(p.u.)	0.994165	0.977595	1.021614	Q_C110_(MVAR)	0.95847	0.968503	0.993119
P_G56_(MW)	59.87608	34.6835	34.7675	P_G111_(MW)	40.8	47.40834	100.0627	V_G56_(p.u.)	0.980759	0.977739	1.019252	V_G111_(p.u.)	1.013245	0.983035	1.042818	**Fuel cost ($/h)**	**136606.5**	139043.9	148666.5
P_G59_(MW)	85.32329	100.6228	77.91559	P_G112_(MW)	30.0859	33.4166	42.51033	V_G59_(p.u.)	0.99411	0.990376	1.020518	V_G112_(p.u.)	0.987536	0.968335	1.035621	Power loss (MW)	66.04785	60.64917	69.88713
P_G61_(MW)	121.1026	171.3857	190.5341	P_G113_(MW)	30.00057	30.00471	60.46362	V_G61_(p.u.)	0.984512	0.977764	1.049388	V_G113_(p.u.)	1.001262	0.98193	1.027175	VD (p.u.)	1.17271	1.736686	1.341578
P_G62_(MW)	66.35149	32.91431	34.51078	P_G116_(MW)	33.11379	32.93348	54.38225	V_G62_(p.u.)	0.987134	0.974194	1.035496	V_G116_(p.u.)	0.975992	0.973302	1.005957				

In contrast to case 1, where no emissions penalty was applied, this scenario illustrates a more significant integration of RES within the optimal generation schedule. The degree of RES penetration is predominantly driven by the emission levels of thermal units and the magnitude of the carbon tax. This relationship is clearly reflected in the simulation results presented in Table 3.

**Table 3:** Simulation outcomes for Case 4.

Control Variables	Min	Max	ARINFO	INFO	AHA	ARO	PSO	SOA	MFO
PTG1 (MW)	50	140	129.3215	128.0741	129.7244	134.8012	128.0511	128.4417	127.3314
PTG2 (MW)	20	80	34.27885	33.04364	33.4827	33.16524	31.28843	32.9588	33.7575
PTG3 (MW)	10	35	10	29.0728	10.00121	17.2158	10	10	10
PwG1 (MW)	0	75	46.4877	45.8143	46.02623	45.7414	44.9225	45.8068	46.2200
PwG2 (MW)	0	60	39.2088	38.71568	38.7691	38.4580	37.9289	38.58183	38.9909
PsG1 (MW)	0	50	34.4350	37.1363	36.4452	36.058	41.3883	37.68131	35.6206
V1 (p.u)	0.95	1.1	1.1	1.0984	1.0999	1.0885	1.1	1.1	1.1
V2 (p.u)	0.95	1.1	1.0899	1.0883	1.0900	1.0894	1.0898	0.95287	1.0901
V5 (p.u)	0.95	1.1	1.0712	1.0711	1.0712	1.0598	1.0711	1.08675	1.0715
V8 (p.u)	0.95	1.1	1.0999	1.1	1.0939	1.0658	1.0882	1.07966	1.0999
V11 (p.u)	0.95	1.1	1.1	1.09843	1.0999	1.0885	1.1	1.1	1.1
V13 (p.u)	0.95	1.1	1.0938	1.09485	1.0943	1.0833	1.0962	1.1	1.1
**Parameters**	**Min**	**Max**	**ARINFO**	**INFO**	**AHA**	**ARO**	**PSO**	**SOA**	**MFO**
QTG1 (MVAr)	−20	150	−10.393191	−6.58101	−10.7578	−20	−10.5288	12.5016	−11.21645
QTG2 (MVAr)	−20	60	16.8437	10.8847	17.2074	42.14811	16.4321	−20	16.2215
QTG3 (MVAr)	−15	40	40	40	40	33.8598	40	40	40
QwG1 (MVAr)	−30	35	24.57232	24.6556	24.5369	17.13307	24.66129	35	24.4413
QwG2 (MVAr)	−25	30	19.0787	17.8632	18.9585	17.8314	18.78418	19.77631	18.4966
QsG1 (MVAr)	−20	25	21.2196	21.21549	21.31784	20.0737	21.89481	24.79181	23.29316
Total cost ($/h)			**808.7305**	809.73949	810.14767	809.01471	810.6223	810.03495	810.00640
Emissions (t/h)			0.316315	0.916198	0.89973	0.63970	0.859230	0.89009	0.906767
Carbon tax ($/h)			20	20	20	20	20	20	20
P_loss_ (MW)			4.05035	5.01561	5.01317	4.81156	5.00725	5.12865	5.01361
V_d_ (p.u)			1.0604	1.094111	1.06528	0.851438	1.078614	1.03324	1.106344
Computational time (sec)			373.517	773.31903	433.1432	439.31884	883.254	1495.919	379.624

**Fig 14:**
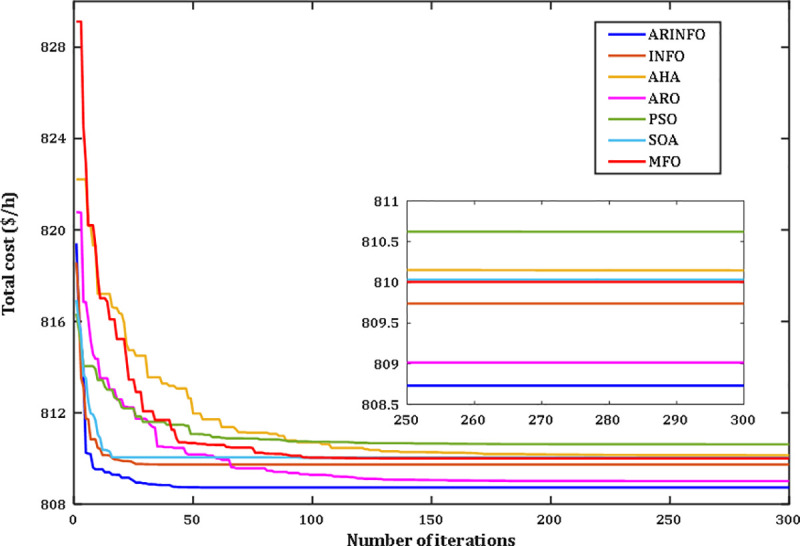
Convergence performance comparison for Case 4.

The convergence behavior of the ARINFO algorithm, alongside competing algorithms, is illustrated in Fig 14, showcasing ARINFO’s superior performance in minimizing total generation costs. The algorithm achieves a minimum cost of 808.7305$/h with a commendably low computational time of 373.517sec.

### 7.5. Case 5: Minimizing Carbon Emissions

This scenario centers exclusively on minimizing greenhouse gas emissions by applying the ARINFO algorithm, as outlined in equation (44). The system configuration comprises three thermal generation units identified as significant contributors to carbon emissions. In contrast to previous cases, the objective in this instance focuses solely on emission reduction, excluding cost considerations.

**Fig 15:**
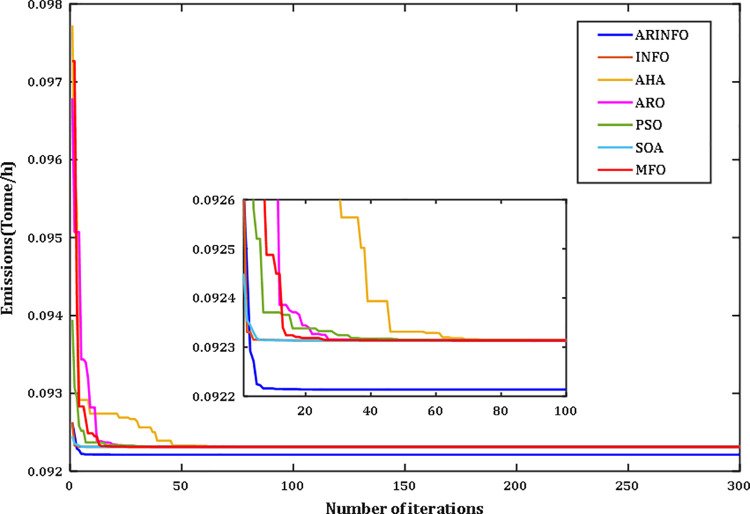
Convergence performance comparison for Case 5.

As shown in Table 4, this environmentally focused strategy substantially decreases total emissions compared to case 1, though it does incur higher generation costs. The ARINFO algorithm is exceptionally effective in this context, surpassing not only its predecessor INFO but also other benchmark optimization methods in terms of emission reduction and convergence efficiency, as illustrated in Fig 15.

**Table 4:** Simulation outcomes for Case 5.

Control variables	Min	Max	ARINFO	INFO	AHA	ARO	PSO	SOA	MFO
PTG1 (MW)	50	140	50	50.0002	50	50.0034	60.001	50.00021	50
PTG2 (MW)	20	80	46.63393	46.55622	40.6008	46.6199	46.63411	46.64063	46.63393
PTG3 (MW)	10	35	35	35	30.47172	35	35	34.9999	35
PwG1 (MW)	0	75	64.7230	74.98588	64.676384	70.61586	71.84848	72.43418	68.8080
PwG2 (MW)	0	60	59.9726	60	44.89753	57.03922	58.53463	48.79917	52.78028
PsG1 (MW)	0	50	48.06443	36.516714	39.19058	43.3449	40.621899	50	50
V1 (p.u)	0.95	1.1	1.0656	0.95	1.03302	1.02224	1.03049	0.96449	1.00734
V2 (p.u)	0.95	1.1	1.0397	1.0981	0.96442	0.99217	1.00971	1.1	1.09806
V5 (p.u)	0.95	1.1	0.95	1.0913	1.09138	1.06057	1.05048	1.05817	0.95279
V8 (p.u)	0.95	1.1	0.9978	0.9500	0.99626	0.98793	1.04573	1.01124	1.09922
V11 (p.u)	0.95	1.1	0.9795	1.0807	1.04623	1.03250	0.97898	0.97894	0.95
V13 (p.u)	0.95	1.1	1.0974	1.03374	1.05404	1.03272	1.03970	0.95238	1.06567
**Parameters**	**Min**	**Max**	**ARINFO**	**INFO**	**AHA**	**ARO**	**PSO**	**SOA**	**MFO**
QTG1 (MVAr)	−20	150	59.59882	−20	25.83583	29.63026	27.79434	−20	−4.269424
QTG2 (MVAr)	−20	60	34.728979	60	−20	−20	−20	60	60
QTG3 (MVAr)	−15	40	24.48832	−15	21.5728	19.48032	40	34.36046	40
QwG1 (MVAr)	−30	35	−30	35	35	35	35	35	−8.06928
QwG2 (MVAr)	−25	30	7.462212	30	25.73186	25.74111	6.5661807	6.55754	5.25706
QsG1 (MVAr)	−20	25	25	25	25	25	25	−1.21864	25
Total cost ($/h)			**831.7751**	832.2834	835.32683	833.6648	838.5869	832.6604	832.984531
Emissions (t/h)			**0.0922140**	0.0923142	0.111367	0.0923140	0.0923145	0.095382	0.093761
Carbon tax ($/h)			0	0	0	0	0	0	0
P_loss_ (MW)			2.312044	2.9966343	3.2673671	2.585178	2.557298	2.981521	3.140319
V_d_ (p.u)			0.9745378	0.7003114	0.702690	0.922331	0.923214	1.23507	1.547840
Computational time (sec)			430.1921	1233.560	500.806	476.423	1061.652	1460.8482	512.225

### 7.6. Case 6: Minimization of Active Power Losses

**Fig 16:**
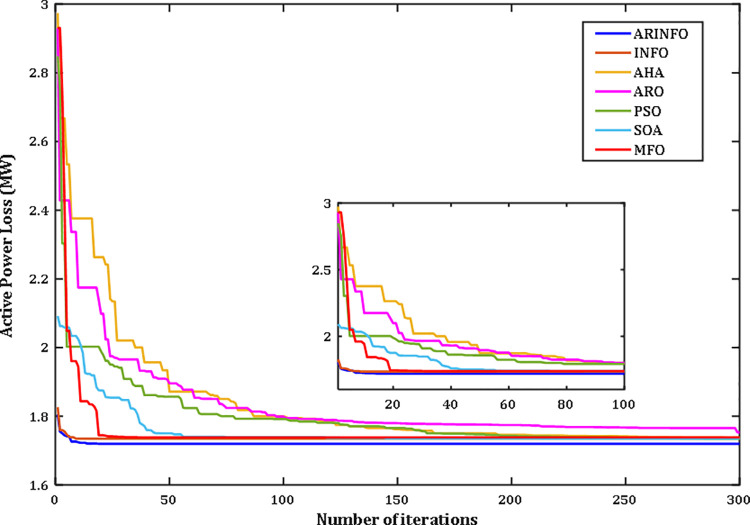
Convergence performance comparison for Case 6.

**Fig 17:**
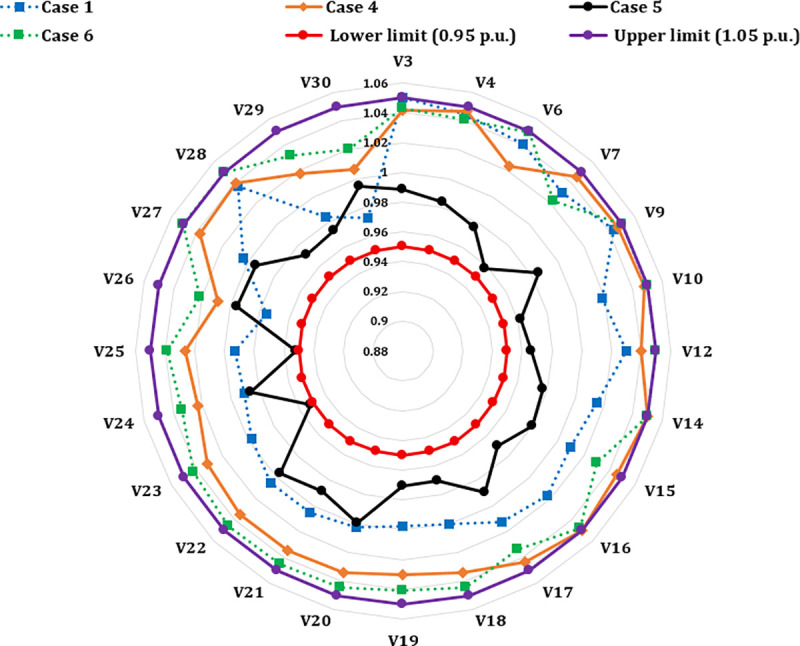
Load bus voltage profiles of the IEEE 30-bus power system in Cases 1, 4, 5, and 6 using the ARINFO optimization algorithm.

A primary objective in OPF analysis is the reduction of active power losses. This case specifically focuses on that goal, adhering to the formulation outlined in equation (46). The findings from this analysis are summarized in Table 5 and illustrated in Fig 16. Among the algorithms assessed, the ARINFO method achieved the lowest power loss and demonstrated the fastest convergence rate, outperforming both the original INFO method and other comparative techniques. Furthermore, Fig 17 presents the voltage profiles of the load buses in the IEEE 30-bus system for cases 1, 4, 5, and 6, employing the ARINFO algorithm. This confirms that all bus voltages remain within the acceptable range of 0.95 to 1.05 p.u., thus fulfilling standard voltage stability criteria.

**Table 5:** Simulation outcomes for Case 6.

Control Variables	Min	Max	ARINFO	INFO	AHA	ARO	PSO	SOA	MFO
PTG1 (MW)	50	140	50.0001	50.0008	50	50.0005	50.001	50.0009	50.0063
PTG2 (MW)	20	80	60.74111	61.74152	61.4002	61.03945	60.6131	59.32846	58.2567
PTG3 (MW)	10	35	35	35	34.9423	20.44241	34.9999	35	35
PwG1 (MW)	0	75	75	75	74.9801	74.99501	74.9998	75	75
PwG2 (MW)	0	60	59.9999	59.9999	59.9371	59.97042	59.9998	60	60
PsG1 (MW)	0	50	46.43904	46.43906	47.0729	46.08405	46.27325	47.53418	50
V1 (p.u)	0.95	1.1	1.1	1.1	1.0987	1.08963	1.09997	1.1	1.1
V2 (p.u)	0.95	1.1	1.1	1.09205	1.0993	1.08956	1.09999	1.1	1.1
V5 (p.u)	0.95	1.1	1.090774	1.09077	1.0890	1.07934	1.09105	1.0908	1.0909
V8 (p.u)	0.95	1.1	1.1	1.09999	0.9795	1.09654	1.09664	1.0972	1.0999
V11 (p.u)	0.95	1.1	1.1	1.07647	1.0999	1.09926	1.1	1.1	1.1
V13 (p.u)	0.95	1.1	1.1	1.00939	1.0987	1.09140	1.09994	1.0999	1.1
**Parameters**	**Min**	**Max**	**ARINFO**	**INFO**	**AHA**	**ARO**	**PSO**	**SOA**	**MFO**
QTG1 (MVAr)	−20	150	−3.27552	21.26649	4.211894	−7.20065	−3.43236	−3.45724	−3.18189
QTG2 (MVAr)	−20	60	9.0063	0.435392	35.47508	11.07981	8.90445	9.27507	9.231124
QTG3 (MVAr)	−15	40	40	40	−15	40	40	40	40
QwG1 (MVAr)	−30	35	22.27052	35	34.45738	21.79731	22.5561	22.29690	22.2908
QwG2 (MVAr)	−25	30	16.5823	18.115849	23.8451	20.20770	16.56960	16.58324	16.58263
QsG1 (MVAr)	−20	25	19.61015	−6.26284	25	20.11411	19.57760	19.59317	19.61437
Total cost ($/h)			**857.0996**	859.48714	860.81997	859.81449	859.31451	858.81630	859.55018
Emissions (t/h)			0.0945328	0.100033	0.099851	0.0963021	0.099380	0.0991207	0.099536
P_loss_ (MW)			1.734974	2.075970	2.47744	1.972884	1.735032	1.735386	1.73864
V_d_ (p.u)			1.341872	0.59158	0.943104	1.09740	1.342475	1.341982	1.342579
Computational time (sec)			323.8169	774.447	420.6641	550.5078	881.326	1095.657	385.967

### 7.7. Case 7: Incorporating Thermal Generator Ramp-Rate Constraints

**Fig 18:**
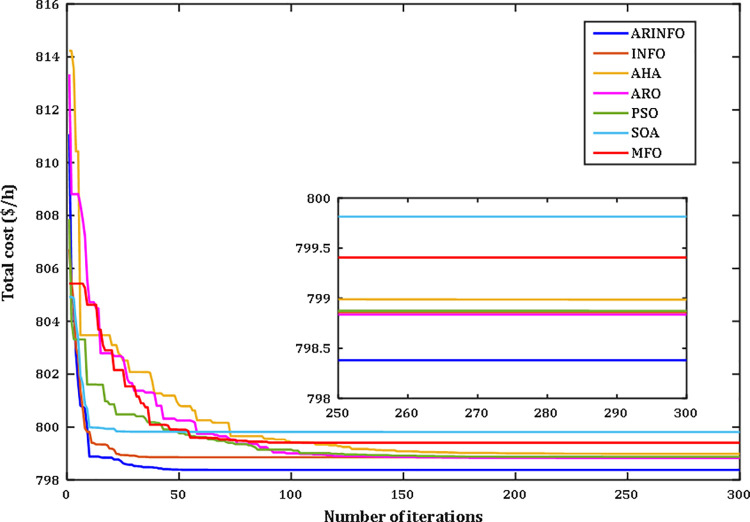
Comparative convergence performance for Case 7.

In this scenario, the objective remains the minimization of total generation costs, as in case 1. However, unlike case 1, this setup introduces ramp-rate limitations for TPGs. The previous hour’s output of each TPG, along with their respective ramp-rate limits, is detailed in Table A1 in Appendix A. Table 6 summarizes the optimal results under these constraints, while Fig 18 illustrates the convergence behaviors of the various algorithms tested.

**Table 6:** Simulation outcomes for Case 7.

Control Variables	Min	Max	ARINFO	INFO	AHA	ARO	PSO	SOA	MFO
PTG1 (MW)	79.211	114.211	114.211	114.211	95.709	104.101	94.911	98.981	113.871
PTG2 (MW)	65	80	65	65.83815	65.2695	65.00968	65. 33157	65. 5801	65
PTG3 (MW)	12	24	12	18.09695	12.00	19.0010	12	12	18.7927
PwG1 (MW)	0	75	46.45379	45.2250	44.9433	45.7723	46.00275	46.79038	45.3382
PwG2 (MW)	0	60	39.1814	40.0490	41.1466	38.6316	38.8179	39.35074	39.54246
PsG1 (MW)	0	50	34.58564	38.1835	38.1637	37.45214	36.5651	33.6605	38.27502
V1 (p.u)	0.95	1.1	1.1	1.1	1.0999	1.0999	1.1	1.1	1.1
V2 (p.u)	0.95	1.1	1.0899	1.0899	1.0893	1.0894	1.0898	0.9526	1.09046
V5 (p.u)	0.95	1.1	1.0712	1.0815	1.0706	1.0700	1.0712	1.0825	1.0798
V8 (p.u)	0.95	1.1	1.1	1.0999	1.07461	1.0963	1.0804	1.0977	1.1
V11 (p.u)	0.95	1.1	1.1	0.9997	1.0999	1.0998	1.1	1.1	1.1
V13 (p.u)	0.95	1.1	1.0938	0.9701	1.0955	1.0958	1.0943	1.0999	1.1
**Parameters**	**Min**	**Max**	**ARINFO**	**INFO**	**AHA**	**ARO**	**PSO**	**SOA**	**MFO**
QTG1 (MVAr)	−20	150	−10.3981	2.3519	−9.4085	−9.5357	−10.3698	12.4199	−11.141
QTG2 (MVAr)	−20	60	16.8364	50.345	15.7429	16.0419	16.6348	−20	16.1939
QTG3 (MVAr)	−15	40	40	40	39.2620	40	40	40	40
QwG1 (MVAr)	−30	35	24.5727	35	24.8562	23.8571	24.6480	35	24.4945
QwG2 (MVAr)	−25	30	19.0717	0.22913	19.0027	18.9381	19.0020	19.8203	18.4565
QsG1 (MVAr)	−20	25	21.2338	−9.49004	21.8476	21.9944	21.3586	24.9116	23.1916
Total cost ($/h)			**798.3816**	798.8598	798.9862	798.8371	798.8748	799.8150	799.4069
Emissions (t/h)			0.212004	0.30536	0.85928	0.892427	0.89999	0.92404	0.874843
P_loss_ (MW)			3.01519	3.78380	5.00421	5.01227	5.01134	5.13522	5.00774
V_d_ (p.u)			0.502091	0.79902	1.0638	1.067585	1.06492	1.03314	1.10642
Computational time (sec)			385.846	397.125	429.883	390.096	467.561	440.278	396.2877

The simulation results clearly indicate that applying ramp-rate limits on TPGs leads to an increase in total generation costs. This increment occurs because the operating points of TPGs are adjusted away from their economically optimal positions to meet the ramping constraints. Nevertheless, the ARINFO algorithm demonstrated its superiority, achieving the lowest generation cost of 798.3816 $/h, with rapid convergence and a modest computational time of 385.846 sec, surpassing the performance of the other evaluated techniques.

**Fig 19:**
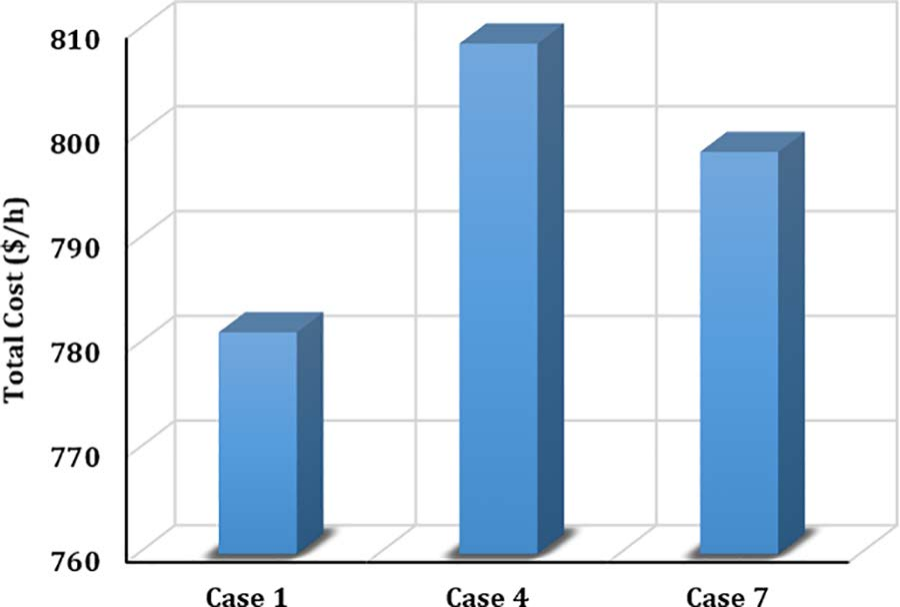
Comparative analysis of total generation costs in Cases 1, 4, and 7.

Fig 19 presents a comparative analysis of total generation costs for cases 1, 4, and 7 utilizing the ARINFO approach. The findings highlight that the implementation of ramp-rate constraints, along with the introduction of a carbon tax on thermal generators, leads to an increase in overall generation costs.

### 7.8. Case 8: Load Demand Uncertainty Analysis

This scenario assesses the performance of the ARINFO algorithm in the context of load demand uncertainty. A load-based scenario analysis is conducted to investigate the OPF under various system loading conditions. The uncertainty in demand is modeled using a normal PDF, characterized by a mean load (*l*_*d*_) of 70 and a standard deviation (*r*_*d*_) of 10, as referenced in [47]. Four distinct loading scenarios are defined, each with specific probabilities and mean values, as outlined in Table 7.

**Table 7:** Mean values and probabilities of different loading scenarios [47].

Loading scenario (*i*)	%Loading, *P*^*-*^_*ld,i*_ (Mean)	Probability, Δ_*ld,i*_
**1**	54.749	0.15866
**2**	65.401	0.34134
**3**	74.599	0.34134
**4**	85.251	0.15866

**Fig 20:**
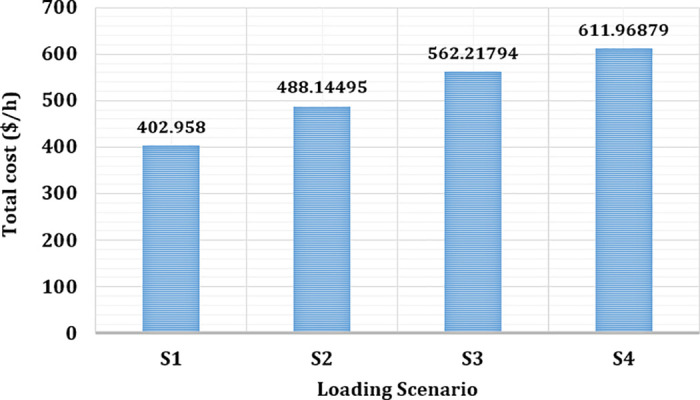
Total cost under versus loading scenarios.

**Fig 21:**
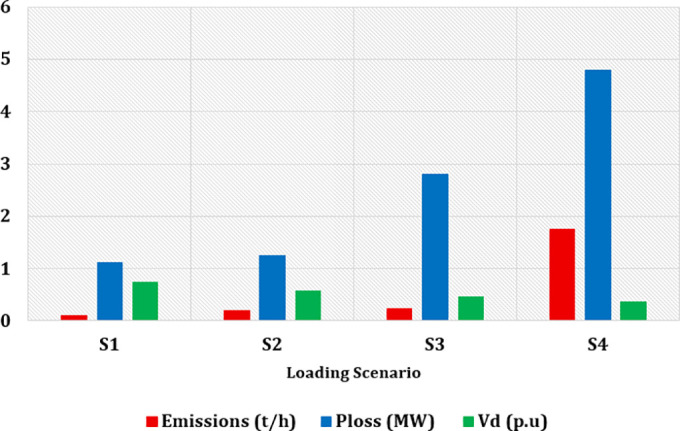
Impact of loading level on voltage deviation, power losses, and carbon emissions.

**Fig 22:**
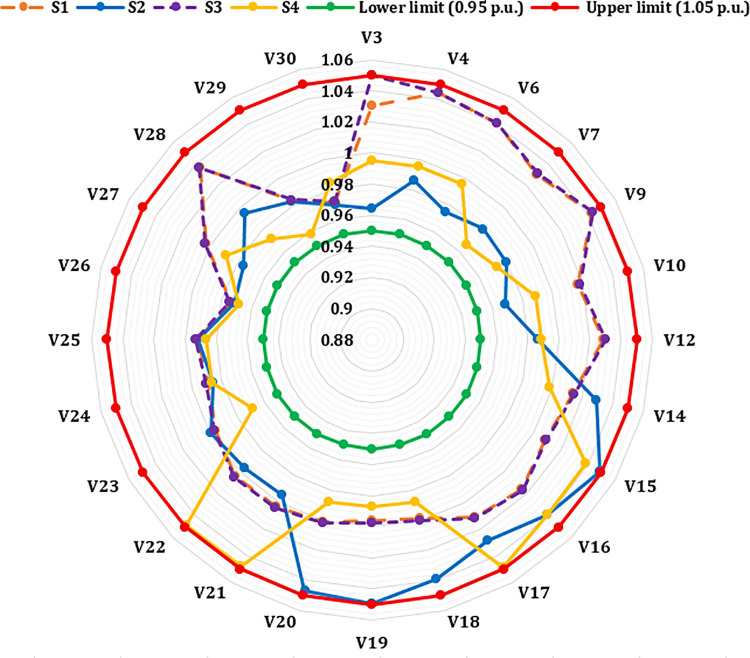
Voltages of the load buses during the 4 loading scenarios.

The simulation results for these scenarios, presented in Table 8, reveal a clear trend: as system loading increases, so does the total generation cost, as depicted in Fig 20. Moreover, both total power losses (*P*_*loss*_) and carbon emissions exhibit an upward trend with rising demand while voltage deviation (*V*_*d*_) decreases, as illustrated in Fig 21. Importantly, all system operational constraints remain satisfied across all scenarios, with load bus voltages consistently maintained within the acceptable range of 0.95 to 1.05 p.u., as shown in Fig 22. These findings confirm the robustness of the ARINFO algorithm in managing load uncertainty while ensuring system reliability and performance.

**Table 8:** Simulation outcomes for Case 8.

Control Variables	Min	Max	Loading scenario 1 (S1)		Loading scenario 2 (S2)		Loading scenario 3 (S3)		Loading scenario 4 (S4)	
			ARINFO	INFO	ARINFO	INFO	ARINFO	INFO	ARINFO	INFO
PTG1 (MW)	50	140	50	50. 01644	54. 07132	52. 09312	114. 07693	114. 31097	134.8755	134.8755
PTG2 (MW)	20	80	26.59845	27.08013	36.57801	29.021798	27.60720	27.988114	22.92747	28.9173
PTG3 (MW)	10	35	10	10.08655	10.33377	10	10	10.018987	10	10.0122
PwG1 (MW)	0	75	42.66540	43.631231	62.56868	43.8423	42.92752	43.43655	34.85643	44.2117
PwG2 (MW)	0	60	36.10553	36.34195	8.264984	37.05852	36.250527	35.968192	29.510008	37.42562
PsG1 (MW)	0	50	38.92988	37.59437	50	34.58280	37.511804	36.892465	29.60954	33.41877
V1 (p.u)	0.95	1.1	1.07201	1.08132	0.95	1.07174	1.07115	1.07299	0.95	1.07464
V2 (p.u)	0.95	1.1	1.05658	1.05700	1.1	1.06552	1.05668	1.05794	0.95	1.05589
V5 (p.u)	0.95	1.1	1.03345	1.04389	0.95	1.03480	1.03477	1.03559	0.95	1.03261
V8 (p.u)	0.95	1.1	1.09999	1.03968	0.95	1.05138	1.05662	1.04015	0.95	1.03849
V11 (p.u)	0.95	1.1	1.1	0.98843	0.95	1.09938	1.09826	1.09999	0.95	1.0955
V13 (p.u)	0.95	1.1	1.046745	1.05532	1.1	1.04892	1.04889	1.04003	0.95	1.0441
**Parameters**	**Min**	**Max**	**ARINFO**	**INFO**	**ARINFO**	**INFO**	**ARINFO**	**INFO**	**ARINFO**	**INFO**
QTG1 (MVAr)	−20	150	−20	−20	−20	−0.99562	−3.58327	7.1979562	−1.282155	22.63425
QTG2 (MVAr)	−20	60	12.24476	−1.078993	12.847543	7.12554	41.79016	14.82992	60	37.988970
QTG3 (MVAr)	−15	40	38.73260	36.705469	40	37.43439	40	40	40	40
QwG1 (MVAr)	−30	35	14.052226	15.65615	21.75189	21.969673	22.744514	23.49468	35	35
QwG2 (MVAr)	−25	30	9.3989261	−3.359116	17.92888	29.303528	30	30	30	30
QsG1 (MVAr)	−20	25	14.42186	12.3302423	15.06701	13.748370	23.230151	14.241802	25	24.732114
Total cost ($/h)			402.9580	403.50550	488.14495	488.4511296	562.21794	562.668648	611.96879	612.487847
Emissions (t/h)			0.1026255	0.10481597	0.2027504	0.2073877	0.2364721	0.27633169	1.7623012	1.79577241
P_loss_ (MW)			1.108071	1.1103438	1.23952	1.7960756	2.804966	3.8034771	4.7916379	5.789299
V_d_ (p.u)			0.7490751	0.5521683	0.56414786	0.4801199	0.4545655	0.43831137	0.36915441	0.4354799

### 7.9. Case 9: Minimizing Total Generation Cost in the IEEE-57 Bus System

This scenario examines the effectiveness of the ARINFO algorithm in tackling the OPF challenge within the intricate IEEE 57-bus network, with a primary focus on minimizing total generation costs. The performance of ARINFO is compared against several other well-known metaheuristic methods, including ARO, AHA, PSO, SOA, MFO, and the original INFO, all under uniform simulation conditions. The objective function and constraints are consistent with those utilized in the IEEE 30-bus system, as outlined in equation (42).

The enhanced configuration of the IEEE 57-bus network features four thermal generation units at buses 1 (swing), 3, 8, and 12, two wind power plants at buses 2 and 6, and a solar PV unit at bus 9. The cost and emission coefficients for the thermal units are detailed in Table 11, while the parameters for the Weibull and lognormal PDFs are listed in Table A2 in Appendix A in S1 File. The network’s total active and reactive load demands are 1250.8 MW and 336.4 MVAR, respectively.

**Fig 23:**
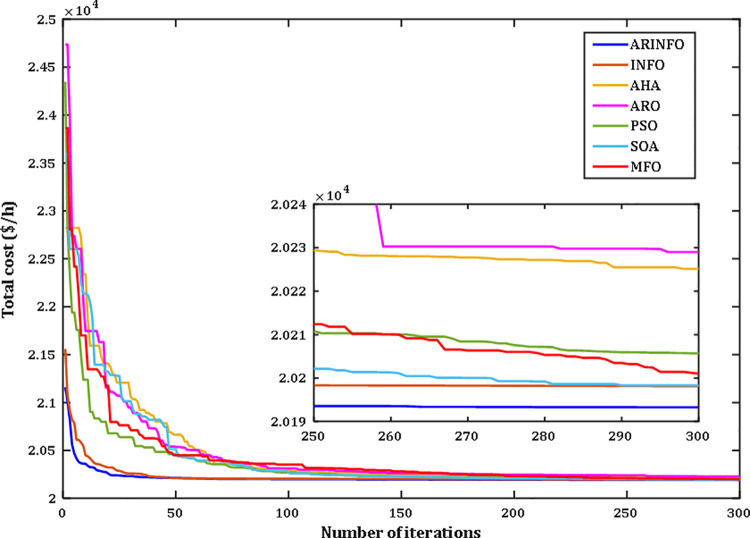
Comparative convergence performance for Case 9.

**Fig 24:**
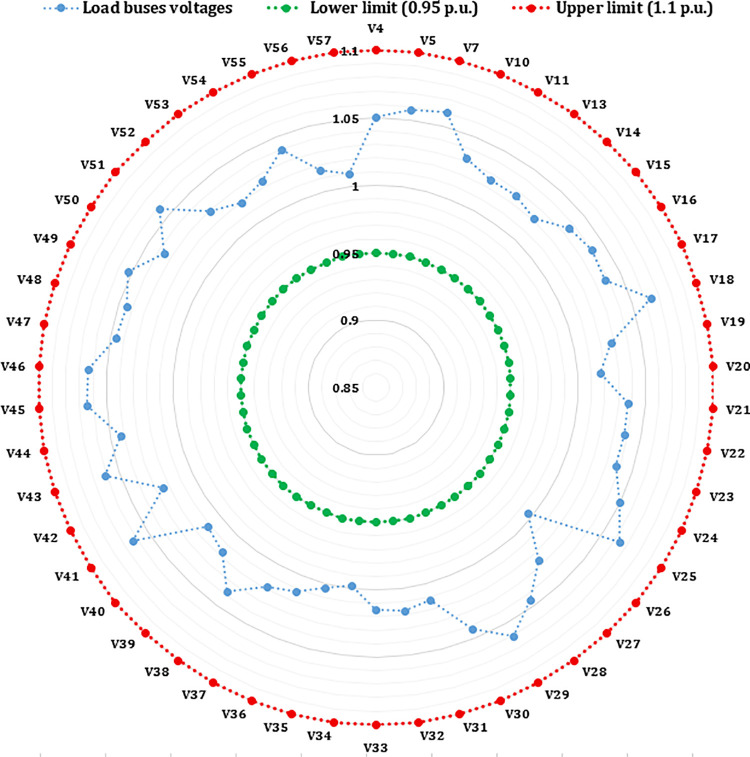
Voltages of the load buses of the modified IEEE-57 bus power system.

The simulation results, summarized in Table 9, illustrate that ARINFO excels in both cost minimization and convergence performance, as shown in Fig 23. Additionally, the voltage levels at all load buses remain within acceptable limits, as displayed in Fig 24, further confirming the operational viability and robustness of the proposed solution.

**Table 9:** Simulation outcomes for Case 9.

Control Variables	Min	Max	ARINFO	INFO	AHA	ARO	PSO	SOA	MFO
PTG1 (MW)	0	576	99.84139	100	100.45399	169.41877	99.88215	97.51941	84.35518
PTG2 (MW)	40	140	120.66190	119.99979	119.9906	116.434162	118.99980	115.2232	120
PTG3 (MW)	100	550	427.32548	424.4918	417.74347	425.58402	427.2456	408.28019	427.556669
PTG4 (MW)	100	410	342.58367	343.0642	349.6759	338.0172	339.83076	324.86735	344.76831
PwG1 (MW)	30	100	100	100	99.9998	100	99.9998	100	99.99995
PwG2 (MW)	30	100	100	99.9997	100	100	100	99.99994	100
PsG1 (MW)	30	100	100	99.99994	100	100	99.9995	100	99.9998
V1 (p.u)	0.95	1.1	1.04878	1.05225	1.03352	1.03315	1.05383	1.00552	1.023617
V2 (p.u)	0.95	1.1	1.04545	1.04874	1.0299	1.03538	1.05057	1.00290	1.02195
V3 (p.u)	0.95	1.1	1.05211	1.04722	1.02977	1.03126	1.05007	1.00250	1.0305
V6 (p.u)	0.95	1.1	1.06654	1.06467	1.05132	1.05077	1.06528	1.01970	1.069948
V8 (p.u)	0.95	1.1	1.07192	1.07153	1.05277	1.05710	1.07132	1.01975	1.1
V9 (p.u)	0.95	1.1	1.04262	1.04466	1.02795	1.03514	1.04603	0.99342	1.052445
V12 (p.u)	0.95	1.1	1.04323	1.04205	1.02974	1.04376	1.04445	0.98941	1.029517
Qc18 (MVAr)	0	20	4.52334	1.90769	12.7068	7.58812	14.09991	12.3149	20
Qc25 (MVAr)	0	20	11.3914	14.3830	12.4136	15.4815	14.0122	15.2111	14.19711
Qc53 (MVAr)	0	20	12.6317	11.9049	15.2929	13.1429	12.7522	13.1511	7.909254
T19 (p.u)	0.9	1.1	1.018411	1.02175	1.05115	0.96647	1.01401	0.95371	1.1
T20 (p.u)	0.9	1.1	0.937126	0.92676	0.93896	1.00653	1.00219	0.96506	0.988504
T31 (p.u)	0.9	1.1	1.021335	1.00388	1.01408	1.047471	1.02218	0.98939	1.085734
T35 (p.u)	0.9	1.1	0.973325	0.99296	0.98694	1.03436	1.02828	0.98167	0.972952
T36 (p.u)	0.9	1.1	1.044652	1.04685	1.00602	0.98943	0.98585	1.00280	1.1
T37 (p.u)	0.9	1.1	1.05274	1.03353	1.02791	1.01743	1.02286	0.98687	1.056728
T41 (p.u)	0.9	1.1	0.99437	0.99573	0.98075	0.99705	0.99564	0.95265	1.005613
T46 (p.u)	0.9	1.1	0.95843	0.96555	0.96533	0.97087	0.967964	0.93149	1.003055
T54 (p.u)	0.9	1.1	0.90002	0.95919	0.91535	0.99533	0.92255	0.94829	0.901677
T58 (p.u)	0.9	1.1	0.96834	0.96949	0.96230	0.96149	0.96956	0.929651	0.961953
T59 (p.u)	0.9	1.1	0.95070	0.96145	0.95141	0.96427	0.97644	0.92054	0.945587
T65 (p.u)	0.9	1.1	0.97140	0.98203	0.95876	0.99028	0.97982	0.92362	0.96673
T66(p.u)	0.9	1.1	0.92722	0.93374	0.9257	0.93993	0.94719	1.02683	0.900012
T71(p.u)	0.9	1.1	0.96545	0.96282	0.95635	0.94444	0.972705	0.91711	1.099944
T73 (p.u)	0.9	1.1	0.99356	0.99819	1.02432	0.99805	0.99855	0.97227	1.063125
T76 (p.u)	0.9	1.1	0.97302	1.06797	0.96815	1.00984	0.96171	0.94868	0.99679
T80 (p.u)	0.9	1.1	0.99891	1.0003	0.99221	1.00670	1.01094	0.946291	1.013499
**Parameters**	**Min**	**Max**	**ARINFO**	**INFO**	**AHA**	**ARO**	**PSO**	**SOA**	**MFO**
QTG1 (MVAr)	−140	200	32.53525	41.46695	35.86920	−37.846577	39.604760	38.54213	7.42336
QTG2 (MVAr)	−10	60	59.03211	41.04512	30.101735	28.734914	35.93422	46.10501	20.26703
QTG3 (MVAr)	−140	200	53.127958	48.9855	38.225529	36.392731	44.52768	39.17961	127.36774
QTG4 (MVAr)	−150	155	69.95093	62.11624	75.472966	104.1811	64.81952	59.84613	55.36481
QwG1 (MVAr)	−17	50	41.32622	50	48.490561	42.038049	49.827720	48.2952070	48.307470
QwG2 (MVAr)	−8	25	−3.61988	−2.88434	3.995952	20.527230	−7.809265	3.244405	−5.31051
QsG1 (MVAr)	−3	9	−1.759277	9	8.368638	8.1574664	8.914996	4.051122	3.0475960
Total cost ($/h)			**20193.270**	20198.120	20225.145	20229.015	20205.694	20198.344	20201.0280
Emissions (t/h)			0.8832847	0.976589	1.1606777	1.742620	1.179650	1.148918	1.1970562
P_loss_ (MW)			9.539564	10.33119	12.200703	10.258589	12.523003	10.161872	10.0307151
V_d_ (p.u)			1.2034715	1.176589	1.2087988	1.161767	1.356530	1.232914	1.614655
Computational time			**424.527**	468.967	523	439.097	567.612	500	462.336

### 7.10. Case 10: Minimization of Total Generation Cost with Carbon Tax in the IEEE 57-Bus System

This scenario introduces a more complex objective function aimed at minimizing the overall generation cost while incorporating a carbon tax on emissions from thermal generators in the IEEE 57-bus power system. The objective function follows the formulation described in equation (43), consistent with prior scenarios. All system parameters remain identical to those in case 9, with the carbon tax rate set at 20$ per tonne of emissions.

**Fig 25:**
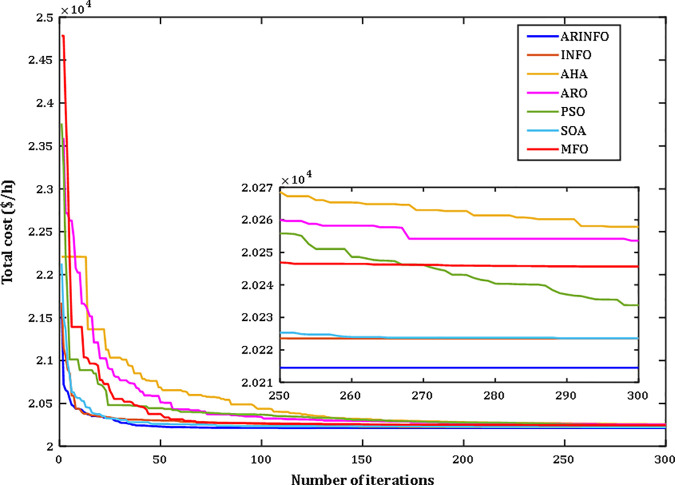
Comparative convergence performance for Case 10.

**Fig 26:**
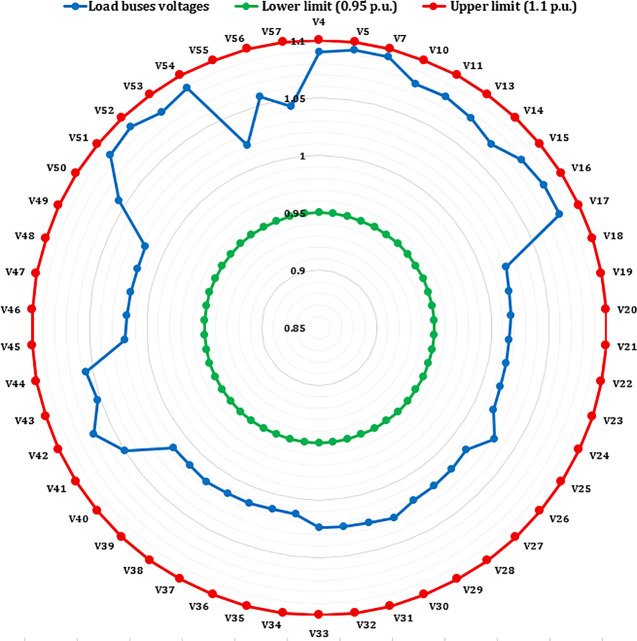
Voltages of the load buses of the modified IEEE-57 bus power system.

The simulation is executed over 30 independent runs, each comprising 300 iterations. The results, detailed in Table 10, indicate that the ARINFO algorithm effectively achieves the lowest total generation cost while maintaining efficient convergence behavior. Comparative convergence trajectories between ARINFO and competing optimization methods, including the original INFO algorithm, are presented in Fig 25, highlighting ARINFO’s superior performance in both cost reduction and convergence speed. Additionally, the voltage magnitudes of all load buses remain within the prescribed operational limits, as confirmed by the profiles in Fig 26.

**Table 10:** Simulation outcomes for Case 10.

Control Variables	Min	Max	ARINFO	INFO	AHA	ARO	PSO	SOA	MFO
PTG1 (MW)	0	576	100.00	100.88421	98.9996	100.54922	100.00	100.0004	100.02112
PTG2 (MW)	40	140	120.61887	119.9999	104.2257	119.9014	119.9999	116.205593	119.9998
PTG3 (MW)	100	550	425.8684	425.79383	383.2065	430.0432	429.76865	424.41764	425.23754
PTG4 (MW)	100	410	340.6682	340.5808	276.2600	335.1918	338.0560	338.70105	340.6382
PwG1 (MW)	30	100	100.00	100.00	100.00	100.00	99.9995	100.00	100.00
PwG2 (MW)	30	100	100.00	99.95232	99.9954	99.99715	100.00	100.00	99.99998
PsG1 (MW)	30	100	100.00	99.99414	99.9998	100.00	100.00	100.00	100.00
V1 (p.u)	0.95	1.1	1.093651	1.09185	1.06758	1.07559	1.05373	1.09283	1.1
V2 (p.u)	0.95	1.1	1.095195	1.09351	1.08915	1.08639	1.05303	1.09433	1.1
V3 (p.u)	0.95	1.1	1.088875	1.08745	1.03984	1.08320	1.05203	1.08855	1.1
V6 (p.u)	0.95	1.1	1.1	1.1	0.95895	1.09929	1.08722	1.1	1.099996
V8 (p.u)	0.95	1.1	1.1	1.1	1.01498	1.05896	1.09678	1.1	1.1
V9 (p.u)	0.95	1.1	1.09995	1.09755	1.02485	1.09083	1.07683	1.09999	1.1
V12 (p.u)	0.95	1.1	1.08383	1.08163	1.06077	1.06945	1.04917	1.08514	1.08748
Qc18 (MVAr)	0	20	10.0671	10.16493	0.04096	12.0111	11.5712	2.15505	16.83649
Qc25 (MVAr)	0	20	10.7774	10.8373	0.07848	11.8151	14.5015	0.734006	10.12604
Qc53 (MVAr)	0	20	9.96215	9.96582	0.06547	11.2877	11.9318	11.36237	14.10792
T19 (p.u)	0.9	1.1	0.9	0.9	0.99010	0.97473	0.92701	0.919907	1.1
T20 (p.u)	0.9	1.1	0.90097	0.9	0.95071	0.96948	0.95525	1.085961	0.9
T31 (p.u)	0.9	1.1	0.98638	0.981941	1.06120	0.98636	0.98849	1.025169	1.029143
T35 (p.u)	0.9	1.1	0.95650	0.95635	0.98268	1.03342	0.94978	0.956566	0.9
T36 (p.u)	0.9	1.1	0.90002	0.90267	0.90440	1.01913	0.97086	1.030335	0.941259
T37 (p.u)	0.9	1.1	1.003926	0.99927	0.93319	0.96598	1.00379	0.990535	1.1
T41 (p.u)	0.9	1.1	0.9	0.97671	0.98514	0.95554	0.958511	0.9716	1.018641
T46 (p.u)	0.9	1.1	0.900001	0.9	1.02861	1.00263	0.92628	1.001221	0.9
T54 (p.u)	0.9	1.1	0.94738	0.9	1.01701	0.98420	0.91568	0.950922	0.931222
T58 (p.u)	0.9	1.1	0.900464	0.90307	0.90733	1.00319	0.94538	1.00278	0.919418
T59 (p.u)	0.9	1.1	0.900013	0.900602	1.02982	0.98142	0.94481	1.00389	0.918402
T65 (p.u)	0.9	1.1	0.9039	0.90583	0.92753	1.00450	0.95649	1.014408	0.919064
T66(p.u)	0.9	1.1	0.9	1.09844	0.99864	0.96495	0.92462	0.97790	0.9
T71(p.u)	0.9	1.1	0.94809	0.9	1.08990	1.01705	0.91053	0.94014	0.948405
T73 (p.u)	0.9	1.1	1.05282	1.00479	1.06451	1.03383	0.96965	0.94453	1.1
T76 (p.u)	0.9	1.1	1.09999	0.98412	0.98077	1.02125	0.97661	0.91384	0.995182
T80 (p.u)	0.9	1.1	0.91140	0.90929	1.00029	0.94372	0.96591	1.00950	1.1
**Parameters**	**Min**	**Max**	**ARINFO**	**INFO**	**AHA**	**ARO**	**PSO**	**SOA**	**MFO**
QTG1 (MVAr)	−140	200	16.703263	20.83689	−72.43408	−30.67917	18.71041	17.39485	24.01416
QTG2 (MVAr)	−10	60	22.88410	33.91447	50.81814	34.50777	59.794971	38.618189	48.8713816
QTG3 (MVAr)	−140	200	−19.42346	−20.84396	35.15070	−135.710	35.94790	−12.290422	−17.085054
QTG4 (MVAr)	−150	155	39.66083	40.37606	149.67478	39.317195	35.20456	49.55836	41.4271867
QwG1 (MVAr)	−17	50	48.085683	49.145168	46.24979	44.28786	38.310043	42.554871	49.7987029
QwG2 (MVAr)	−8	25	−6.448709	−7.279470	−7.17203	23.876759	23.60302	23.8674037	−5.39722
QsG1 (MVAr)	−3	9	3.610565	8.167194	7.6474376	8.96581	6.875416	8.4357013	7.478934
Total cost ($/h)			**20214.5210**	20223.5038	20257.9083	20253.613	20233.7582	20223.605	20245.638
Emissions (t/h)			0.8917688	0.981606	1.879361	1.200461	1.1843277	1.165988	1.175927
P_loss_ (MW)			10.393129	11.489911	12.916379	11.60813	12.599245	10.560719	11.141430
V_d_ (p.u)			1.649701	1.783431	1.874262	1.664985	1.710492	1.66634	1.773407
Computational time			**439.759**	476.981	539.1041	439.592	568.627	481.4857	537.7588

### 7.11. Case 11: Optimization of Fuel Cost for the Large-Scale Test System

To show the effectiveness of the proposed ARINFO for the large-scale test system, it is tested using the IEEE 118-bus system. The line and bus data of the IEEE 118-bus system can be found in [48]. The IEEE 118-bus system has 54 generating units. Bus 69 is chosen as the slack bus. The voltage magnitude limits of all buses are taken between 0.95 p.u. and 1.06 p.u. Also, the tap setting for tap changing transformers is varying between 0.9 p.u. To 1.1 p.u. Moreover, the limit of VAR compensators is assumed to vary between 0 and 0.3 p.u.

**Fig 27:**
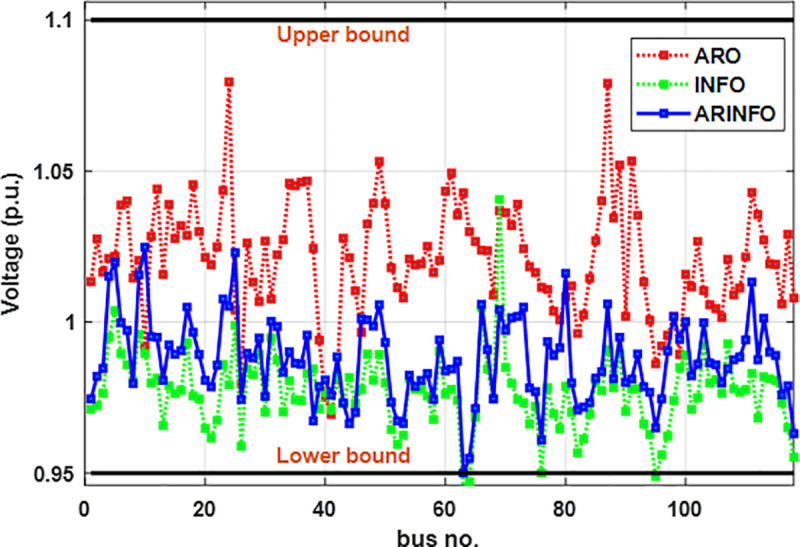
Voltages of the load buses of the IEEE-118 bus power system.

**Fig 28:**
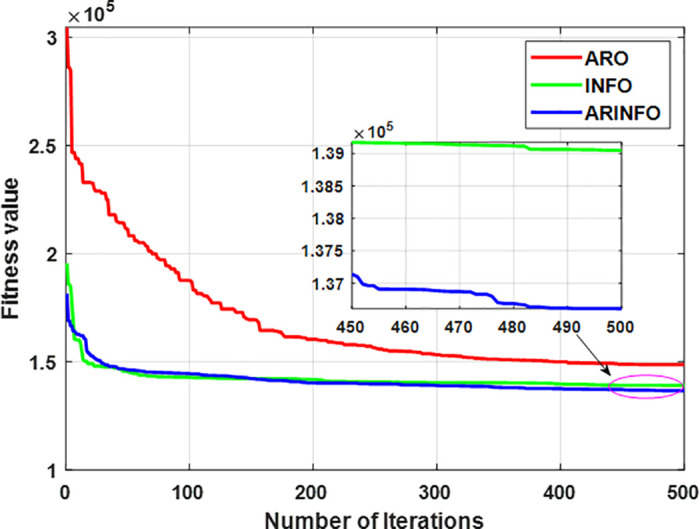
Comparative convergence performance for Case 11.

In this case, the ARINFO is applied to solve the OPF problem considering the fuel cost for the IEEE 118-bus system. This system is employed in this paper to test the scalability of the proposed ARINFO and prove its ability to solve large-scale systems. The obtained results of ARINFO are compared with INFO and ARO in Table 11. The results in Table 11 proves the superiority of the proposed ARINFO over other methods in solving the OPF problem for the large-scale system. The ARINFO’s objective function (136606.5 $/h) is better than the objective function of other methods without any violation of the constraints. The voltage magnitudes of all buses of the ARINFO are within the minimum and maximum limits as shown in Fig 27. In addition, the ARINFO has smooth and fast convergence characteristics in comparison with other methods, as is clear in Fig 28.

## 8. Conclusion

This study presents ARINFO, an enhanced variant of the INFO optimization algorithm, specifically designed to tackle the complexities of the OPF problem amidst the uncertainties that arise from integrating renewable energy sources. ARINFO employs stochastic models to capture the variability of renewable energy outputs, utilizing Weibull and lognormal distributions for wind and solar generation, respectively. Through thorough validation against benchmark functions from the CEC-2017 and CEC-2022 suites, along with a comparative analysis of nine established metaheuristic algorithms, ARINFO showcases superior solution quality, accelerated convergence, and increased robustness across various OPF objectives, including minimizing generation costs (with and without carbon taxation), reducing emissions, and minimizing active power losses.

Furthermore, the study considers the effects of varying reserve and penalty costs, different load demand scenarios, and ramp rate limits for thermal generators, thereby providing a comprehensive framework for managing the inherent uncertainties present in modern power systems.

### 8.1. Broader implications and global context

The findings of this research hold significant implications for the global energy sector’s transition towards sustainability and resilience. The ARINFO algorithm’s capacity to manage renewable energy uncertainty directly addresses the challenges of integrating higher proportions of renewable energy into existing grids. By modeling the costs of overestimation and underestimation, ARINFO enables grid operators to more effectively accommodate intermittent renewable resources, such as wind and solar, without compromising system stability. This capability is crucial for nations aiming to achieve global carbon neutrality goals.

Moreover, ARINFO facilitates the development of resilient and adaptive power systems. Its ability to optimize power flow under varying conditions, whether driven by fluctuating demand or renewable generation, enhances the economic efficiency of power structures. This optimization can lead to considerable savings for utility companies, ultimately lowering electricity costs for consumers while improving the competitiveness of industries on a global scale. Such advancements are particularly advantageous for developing nations rapidly expanding their power infrastructure and seeking to incorporate renewable energy sources into their grids.

The framework’s multi-objective capabilities also contribute directly to climate change mitigation efforts. By balancing cost reduction with emission minimization, ARINFO equips policymakers and system planners with a valuable tool to aid the transition towards cleaner, more sustainable energy solutions. Notably, ARINFO’s effectiveness in optimizing scenarios involving carbon taxation underscores its potential to steer operational strategies that penalize carbon emissions while promoting cleaner energy sources.

Finally, the scalability of ARINFO, as demonstrated through its application to both medium-sized and larger IEEE test systems, indicates its suitability for real-world, large-scale applications in national and regional grids. This scalability is essential for modern power systems, especially in regions rapidly transitioning to renewable energy sources.

### 8.2. Future work

Future research could build upon this study by incorporating energy storage systems to manage the variability of renewables better, investigating real-time OPF scenarios, and applying ARINFO to larger hybrid grid models. Furthermore, the integration of adaptive control mechanisms or the coupling of ARINFO with deep learning models has the potential to enhance predictive capabilities and elevate the performance of next-generation power systems. Also, it is important to consider more detailed emission formulations incorporating startup/shutdown processes, ramping limits, and additional environmental constraints to further enhance the practical realism of the proposed approach in future works.

## Supporting information

S1 File Supplementary Material. Appendix A.Table A1 Cost coefficients of TPGs. Table A2 PDF parameters for wind and solar PV power stations. Fig A1 Weibull PDF distribution of wind speed for WPGs. Fig A2 Lognormal PDF of solar irradiance distribution for solar PV. Fig A3. Actual power distribution of the solar photovoltaic generator Appendix B. Fig B1 Qualitative results (3D-view, search history, average objective function, convergence curve). Table B1 Statistical results of competitive techniques for the CEC-2017 test suite. Table B2 Average ranks and overall rankings on CEC-2017. Table B3 Statistical results of competitive techniques for the CEC-2022 test suite. Table B4 Average ranks and overall rankings on CEC-2022. Fig B2 Convergence curves for CEC-2017. Fig B3 Convergence curves for CEC-2022. Fig B4 Box plots for CEC-2017. Fig B5 Box plots for CEC-2022. Table B5 Wilcoxon rank-sum test results for CEC-2017. Table B6 Wilcoxon rank-sum test results for CEC-2022. Table B7 P-value-based statistical metrics for CEC-2017. Table B8 P-value-based statistical metrics for CEC-2022.
(DOCX)
