# Supplementary material for: Optimal power flow of hybrid wind/solar/thermal energy integrated power systems considering renewable energy uncertainty via an enhanced weighted mean of vectors algorithm
Source: PLoS One. 2026 Feb 10;21(2):e0336157. doi: 10.1371/journal.pone.0336157 (PMC12890154; doi:10.1371/journal.pone.0336157)
Supplement: S1 File — Appendix A.Table A1 Cost coefficients of TPGs. Table A2 PDF parameters for wind and solar PV power stations. Fig A1 Weibull PDF distribution of wind speed for WPGs. Fig A2 Lognormal PDF of solar irradiance distribution for solar PV. Fig A3. Actual power distribution of the solar photovoltaic generator Appendix B. Fig B1 Qualitative results (3D-view, search history, average objective function, convergence curve). Table B1 Statistical results of competitive techniques for the CEC-2017 test suite. Table B2 Average ranks and overall rankings on CEC-2017. Table B3 Statistical results of competitive techniques for the CEC-2022 test suite. Table B4 Average ranks and overall rankings on CEC-2022. Fig B2 Convergence curves for CEC-2017. Fig B3 Convergence curves for CEC-2022. Fig B4 Box plots for CEC-2017. Fig B5 Box plots for CEC-2022. Table B5 Wilcoxon rank-sum test results for CEC-2017. Table B6 Wilcoxon rank-sum test results for CEC-2022. Table B7 P-value-based statistical metrics for CEC-2017. Table B8 P-value-based statistical metrics for CEC-2022. (DOCX) [file pone.0336157.s001.docx]

Appendices

Appendix A

##### **Table A1.** Cost coefficients of TPGs [14].

| TPG | Bus | *a* | *b* | *c* | *d* | *e* | *α* | *β* | *γ* | *ω* | *μ* | *P*^0^*_TGi_*  (MW) | *DR_i_*  (MW) | *UR_i_*  (MW) |
| --- | --- | --- | --- | --- | --- | --- | --- | --- | --- | --- | --- | --- | --- | --- |
| 1 | 1 | 0 | 2 | 0.00375 | 18 | 0.037 | 4.091 | −5.554 | 6.49 | 0.0002 | 6.667 | 99.211 | 20 | 15 |
| 2 | 2 | 0 | 1.75 | 0.0175 | 16 | 0.038 | 2.543 | −6.047 | 5.638 | 0.0005 | 3.333 | 80 | 15 | 10 |
| 3 | 8 | 0 | 3.25 | 0.00834 | 12 | 0.045 | 5.326 | −3.55 | 3.38 | 0.002 | 2 | 20 | 8 | 4 |

##### **Table A2.** The PDF parameters for wind and solar PV power stations.

| **Wind Power Farm** | | | | | **Solar Power Plant** | | |
| --- | --- | --- | --- | --- | --- | --- | --- |
| Wind Power Generator | No. of Turbines | Rated Power (MW) | Weibull PDF Parameters | Weibull Mean, *M_weibull_* | Rated Power (MW) | Lognormal PDF Parameters | Lognormal |
| 1 (at bus 5) | 25 | 75 | *K* = 2, *c* = 9 | *Wd_v_* = 7.976 (m/s) | 50(at bus 13) | *μ* = 6, *δ* = 0.6 | *R*= 483W/m^2^ |
| 2 (at bus11) | 20 | 60 | *K* = 2, *c* = 10 | *Wd_v_* = 8.862 (m/s) |  |  |  |

(a)

(b)

**Fig A1.** The Weibull PDF Distribution of wind speed for WPG: (a) WPG1 located at bus 5, (b) WPG2 located at bus 11.

**Fig A2.** The lognormal PDF of solar irradiance distribution for the solar PV generator at bus 13.

**Fig A3.** Actual power distribution of the solar photovoltaic generator

Appendix B

| 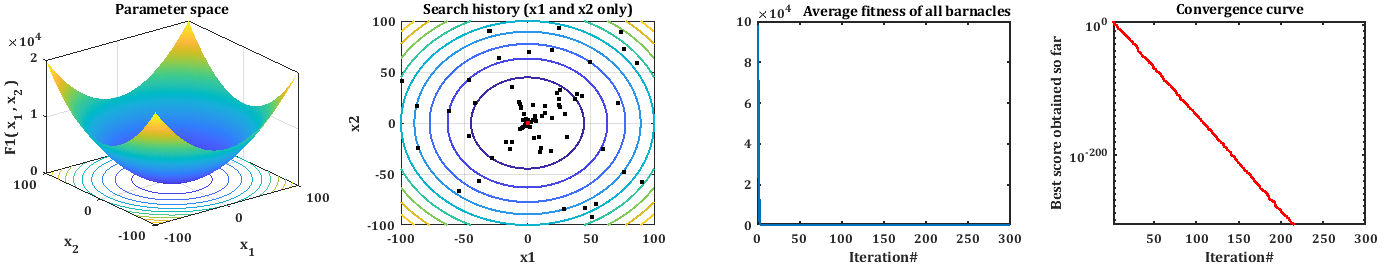 |
| --- |
| 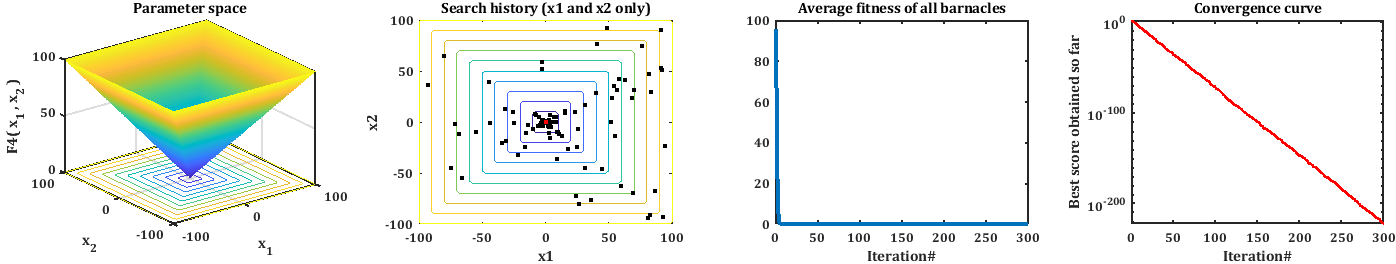 |
| 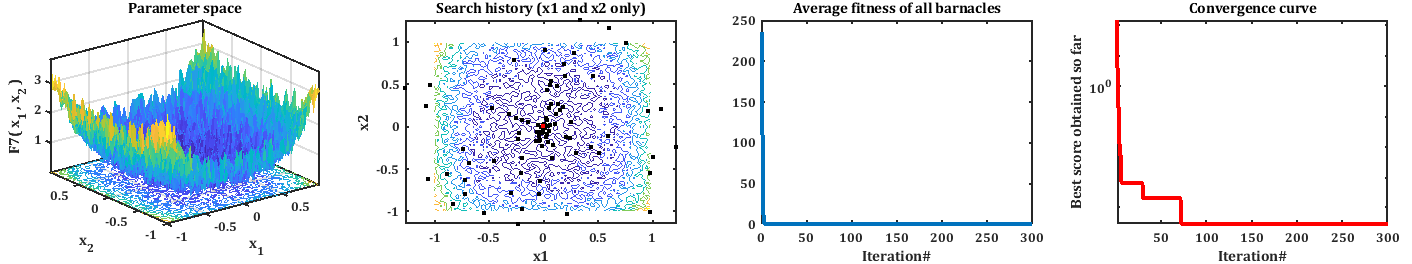 |
| 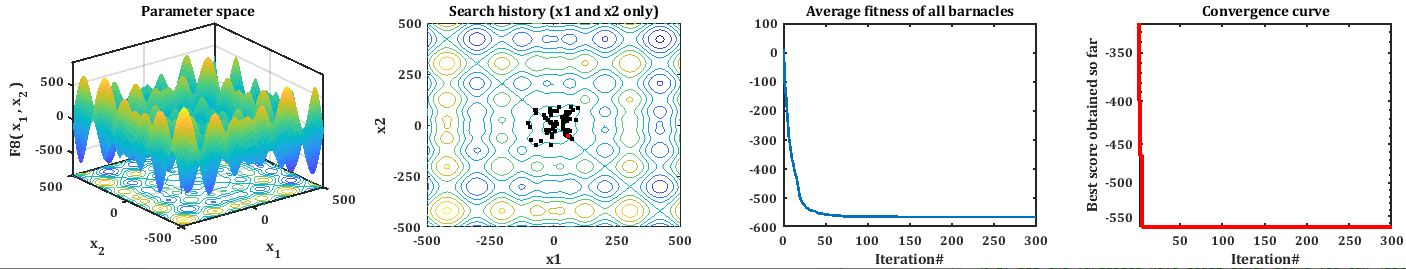 |
| 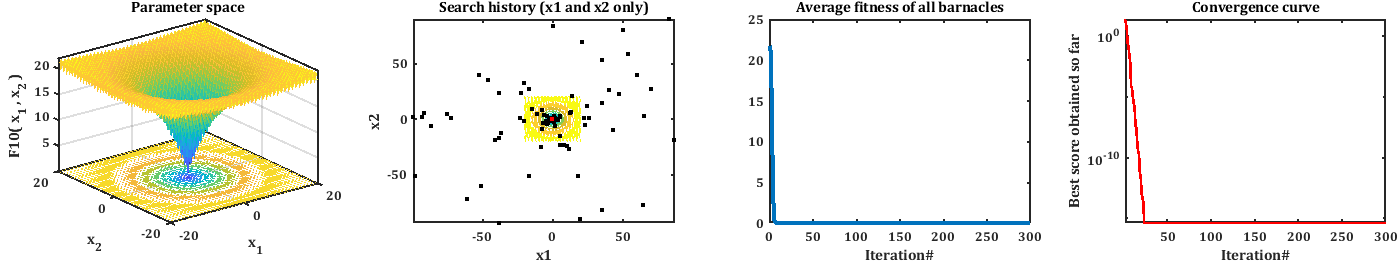 |
| 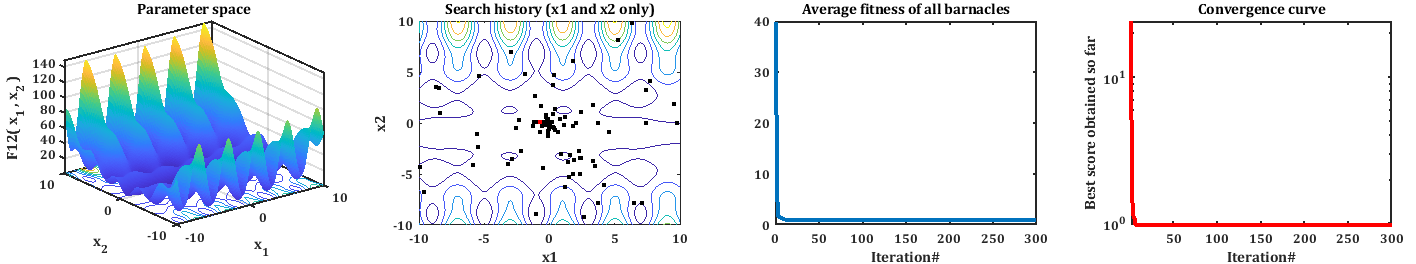 |
| 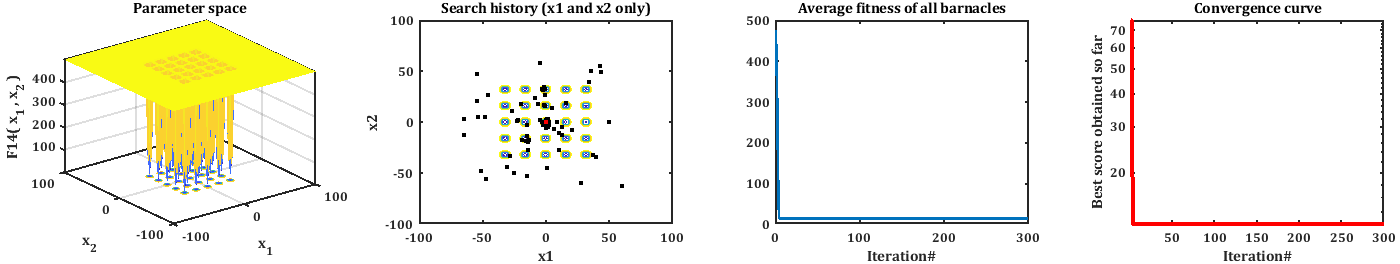 |
| 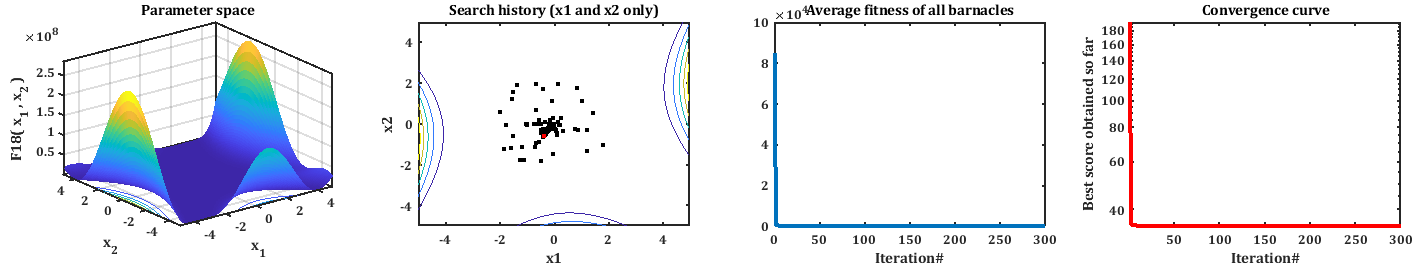 |
| 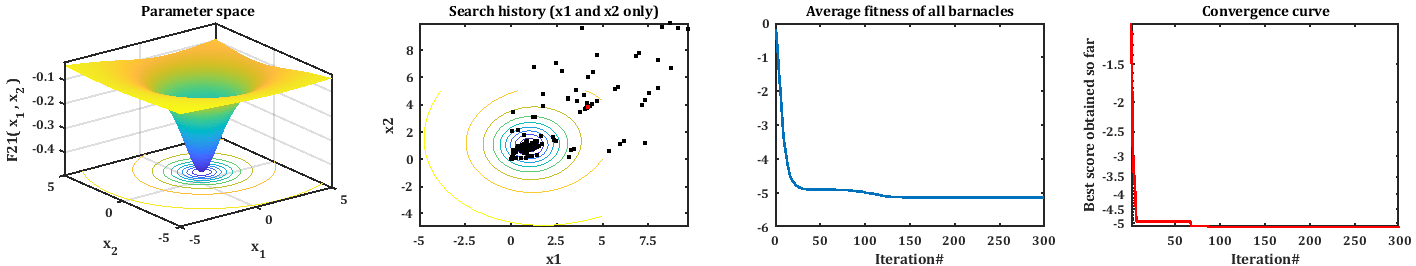 |

**Fig B1.** Qualitative results, including 3D-view, search history, average objective function, and convergence curve.

##### **Table B1.** The statistical results of the competitive techniques for the CEC-2017 test suite

| **Function** | **Tech.** | **Best** | **Worst** | **Mean** | **SD** | **SE** | **Median** | **Rank** |
| --- | --- | --- | --- | --- | --- | --- | --- | --- |
| **F1** | **ARINFO** | **0.000e+00** | **0.000e+00** | **0.000e+00** | **0.000e+00** | **0.000e+00** | **0.000e+00** | **1** |
|  | **INFO** | 7.7314e-56 | 1.3778e-54 | 6.2839e-55 | 3.6622e-55 | 6.6861e-56 | 6.3154e-55 | 6 |
|  | **AHA** | 9.1006e-171 | 2.4541e-151 | 1.4853e-152 | 4.8332e-152 | 8.8241e-153 | 1.9535e-160 | 3 |
|  | **ARO** | 3.3714e-98 | 2.8499e-93 | 2.9396e-94 | 6.853e-94 | 1.2512e-94 | 1.582e-95 | 5 |
|  | **WOA** | 6.4253e-112 | 6.18e-104 | 3.7236e-105 | 1.241e-104 | 2.2657e-105 | 8.4781e-108 | 4 |
|  | **SWO** | 0.0011809 | 43042 | 2932.8 | 9018.6 | 1646.6 | 142.76 | 10 |
|  | **PSO** | 0.00014817 | 0.003525 | 0.00083982 | 0.00070711 | 0.0001291 | 0.00059135 | 7 |
|  | **MFO** | 0.19395 | 10000 | 334.39 | 1825.6 | 333.31 | 0.84774 | 9 |
|  | **SOA** | 7.4275e-247 | 5.3454e-228 | 1.8668e-229 | **0.000e+00** | **0.000e+00** | 8.7835e-236 | 2 |
|  | **SCA** | 0.00021467 | 6.2884 | 0.26407 | 1.1398 | 0.20809 | 0.036054 | 8 |
| **F2** | **ARINFO** | **0.000e+00** | **0.000e+00** | **0.000e+00** | **0.000e+00** | **0.000e+00** | **0.000e+00** | 1 |
|  | **INFO** | 1.4536e-27 | 4.7629e-27 | 3.1701e-27 | 1.0371e-27 | 1.8934e-28 | 3.3506e-27 | 6 |
|  | **AHA** | 2.5079e-85 | 1.2669e-78 | 1.2376e-79 | 3.2754e-79 | 5.9801e-80 | 9.6426e-82 | 3 |
|  | **ARO** | 1.702e-51 | 1.963e-48 | 3.5011e-49 | 4.9285e-49 | 8.9981e-50 | 1.5514e-49 | 5 |
|  | **WOA** | 2.6654e-66 | 8.5234e-59 | 6.4723e-60 | 1.9596e-59 | 3.5777e-60 | 6.6575e-62 | 4 |
|  | **SWO** | 0.058869 | 7.6104e+06 | 2.7609e+05 | 1.3906e+06 | 2.5389e+05 | 13.056 | 10 |
|  | **PSO** | 0.011522 | 0.070619 | 0.037765 | 0.01313 | 0.0023971 | 0.034911 | 8 |
|  | **MFO** | 0.074525 | 70.003 | 29.757 | 18.424 | 3.3638 | 30.058 | 9 |
|  | **SOA** | 4.5056e-127 | 8.0062e-115 | 3.061e-116 | 1.4583e-115 | 2.6625e-116 | 1.0683e-119 | 2 |
|  | **SCA** | 2.0454e-05 | 0.0028273 | 0.00036053 | 0.00050694 | 9.2553e-05 | 0.00026908 | 7 |
| **F3** | **ARINFO** | **0.000e+00** | **0.000e+00** | **0.000e+00** | **0.000e+00** | **0.000e+00** | **0.000e+00** | 1 |
|  | **INFO** | 5.6358e-53 | 1.9807e-51 | 7.6397e-52 | 5.4933e-52 | 1.0029e-52 | 6.1518e-52 | 5 |
|  | **AHA** | 3.5425e-163 | 5.002e-139 | 1.6678e-140 | 9.1322e-140 | 1.6673e-140 | 5.2138e-147 | 3 |
|  | **ARO** | 1.3084e-81 | 8.3091e-77 | 4.8347e-78 | 1.613e-77 | 2.9449e-78 | 1.8479e-79 | 4 |
|  | **WOA** | 1385.1 | 17500 | 8530.4 | 4093.9 | 747.44 | 7905.6 | 9 |
|  | **SWO** | 5.3549 | 53203 | 6295.7 | 10530 | 1922.5 | 2860.5 | 8 |
|  | **PSO** | 6.6613 | 25.873 | 14.657 | 5.3883 | 0.98377 | 14.233 | 6 |
|  | **MFO** | 1149.5 | 30363 | 12232 | 9234.8 | 1686 | 11144 | 10 |
|  | **SOA** | 9.5405e-220 | 1.0099e-192 | 3.3666e-194 | **0.000e+00** | **0.000e+00** | 5.9333e-205 | 2 |
|  | **SCA** | 192.62 | 5500.7 | 1665.3 | 1412.1 | 257.82 | 1226.9 | 7 |
| **F4** | **ARINFO** | **0.000e+00** | **0.000e+00** | **0.000e+00** | **0.000e+00** | **0.000e+00** | **0.000e+00** | 1 |
|  | **INFO** | 6.1712e-29 | 1.1054e-27 | 4.5847e-28 | 2.3659e-28 | 4.3194e-29 | 4.3328e-28 | 5 |
|  | **AHA** | 3.5791e-76 | 1.1133e-70 | 8.3957e-72 | 2.3181e-71 | 4.2323e-72 | 1.1653e-73 | 3 |
|  | **ARO** | 5.9034e-44 | 4.5142e-42 | 9.3857e-43 | 1.246e-42 | 2.2748e-43 | 4.0102e-43 | 4 |
|  | **WOA** | 7.4651e-08 | 77.83 | 12.449 | 18.398 | 3.359 | 2.1935 | 7 |
|  | **SWO** | 0.21176 | 80.636 | 21.969 | 25.669 | 4.6865 | 11.602 | 9 |
|  | **PSO** | 0.27216 | 0.70153 | 0.46718 | 0.10444 | 0.019069 | 0.4627 | 6 |
|  | **MFO** | 15.982 | 44.28 | 28.951 | 6.9939 | 1.2769 | 28.426 | 10 |
|  | **SOA** | 1.0217e-120 | 2.0836e-111 | 1.2594e-112 | 4.2028e-112 | 7.6733e-113 | 2.0469e-114 | 2 |
|  | **SCA** | 2.8899 | 35.921 | 13.34 | 7.4265 | 1.3559 | 12.25 | 8 |
| **F5** | **ARINFO** | 1.7477e-14 | 8.1692 | 0.27231 | 1.4915 | 0.27231 | 3.1046e-10 | 2 |
|  | **INFO** | 12.095 | 17.072 | 14.984 | 1.2676 | 0.23144 | 15.125 | 3 |
|  | **AHA** | 24.144 | 25.818 | 25.169 | 0.42295 | 0.077221 | 25.278 | 5 |
|  | **ARO** | 20.218 | 21.656 | 20.781 | 0.34402 | 0.062809 | 20.756 | 4 |
|  | **WOA** | 0.057068 | 26.998 | 25.244 | 4.765 | 0.86996 | 26.004 | 6 |
|  | **SWO** | 29.104 | 1.1439e+08 | 7.7853e+06 | 2.2016e+07 | 4.0195e+06 | 1135.1 | 10 |
|  | **PSO** | 23.122 | 221.72 | 60.141 | 53.231 | 9.7186 | 30.726 | 7 |
|  | **MFO** | 119.73 | 90245 | 10024 | 27207 | 4967.3 | 532.95 | 9 |
|  | **SOA** | **0.000e+00** | **0.000e+00** | **0.000e+00** | **0.000e+00** | **0.000e+00** | **0.000e+00** | 1 |
|  | **SCA** | 30.325 | 2407.3 | 457.02 | 694.93 | 126.88 | 104.84 | 8 |
| **F6** | **ARINFO** | 2.7733e-32 | 6.0539e-29 | 2.8265e-30 | 1.098e-29 | 2.0046e-30 | 3.3126e-31 | 2 |
|  | **INFO** | 8.0119e-32 | 3.6893e-28 | 2.2568e-29 | 6.805e-29 | 1.2424e-29 | 2.1401e-30 | 3 |
|  | **AHA** | 7.1472e-07 | 6.4282e-06 | 2.8768e-06 | 1.7331e-06 | 3.1642e-07 | 2.2248e-06 | 5 |
|  | **ARO** | 3.9111e-10 | 1.6783e-09 | 9.5187e-10 | 3.3687e-10 | 6.1504e-11 | 9.4974e-10 | 4 |
|  | **WOA** | 0.0001957 | 0.0012975 | 0.00058705 | 0.00027539 | 5.0279e-05 | 0.00053272 | 7 |
|  | **SWO** | 6.7633 | 54994 | 10240 | 17082 | 3118.8 | 1025.5 | 10 |
|  | **PSO** | 9.2466e-05 | 0.0030311 | 0.00052575 | 0.00056703 | 0.00010352 | 0.00041867 | 6 |
|  | **MFO** | 0.25132 | 1.723 | 0.96126 | 0.34773 | 0.063486 | 0.92604 | 8 |
|  | **SOA** | **0.000e+00** | **0.000e+00** | **0.000e+00** | **0.000e+00** | **0.000e+00** | **0.000e+00** | 1 |
|  | **SCA** | 3.0154 | 6.0917 | 4.2143 | 0.73398 | 0.13401 | 4.0673 | 9 |
| **F7** | **ARINFO** | **3.0341e-07** | **4.7534e-05** | **1.4044e-05** | **1.1092e-05** | **2.0251e-06** | **1.0626e-05** | 1 |
|  | **INFO** | 2.052e-05 | 0.0011665 | 0.00031734 | 0.00025717 | 4.6952e-05 | 0.00025042 | 5 |
|  | **AHA** | 4.1716e-06 | 0.00010385 | 5.0838e-05 | 3.0041e-05 | 5.4847e-06 | 4.9839e-05 | 6 |
|  | **ARO** | 3.8073e-07 | 6.7314e-05 | 2.0635e-05 | 1.5906e-05 | 2.904e-06 | 1.7331e-05 | 2 |
|  | **WOA** | 6.2401e-06 | 0.0024751 | 0.00062946 | 0.00071683 | 0.00013088 | 0.0002784 | 6 |
|  | **SWO** | 0.011191 | 73.595 | 7.6706 | 18.811 | 3.4344 | 0.28107 | 10 |
|  | **PSO** | 0.01812 | 0.12529 | 0.056491 | 0.021114 | 0.0038548 | 0.053097 | 8 |
|  | **MFO** | 0.038584 | 10.785 | 1.4921 | 3.0414 | 0.55527 | 0.076481 | 9 |
|  | **SOA** | 1.2335e-06 | 0.00023578 | 8.4335e-05 | 6.2679e-05 | 1.1443e-05 | 7.431e-05 | 4 |
|  | **SCA** | 0.0039487 | 0.053881 | 0.021023 | 0.013707 | 0.0025025 | 0.018129 | 7 |
| **F8** | **ARINFO** | -12451 | -10753 | -11702 | 378.71 | 69.143 | -11740 | 4 |
|  | **INFO** | -10931 | -8714.1 | -9683.7 | 686.45 | 125.33 | -9652.5 | 5 |
|  | **AHA** | -12568 | **-12323** | **-12535** | **59.053** | **10.782** | -12562 | 1 |
|  | **ARO** | **-12569** | -12154 | -12528 | 126.64 | 23.121 | **-12569** | 2 |
|  | **WOA** | **-12569** | -7383 | -11980 | 1151.9 | 210.32 | -12352 | 3 |
|  | **SWO** | -4698.9 | -2929.5 | -3468.1 | 471.15 | 86.019 | -3360.8 | 10 |
|  | **PSO** | -8285.6 | -5738.5 | -6887.7 | 650.1 | 118.69 | -6913.7 | 8 |
|  | **MFO** | -11285 | -7725.9 | -9389.5 | 940.37 | 171.69 | -9410.6 | 6 |
|  | **SOA** | -12346 | -6036.8 | -7822.5 | 1477.8 | 269.8 | -7343 | 7 |
|  | **SCA** | -4756.5 | -3723.1 | -4166.8 | 219.72 | 40.116 | -4169.1 | 9 |
| **F9** | **ARINFO** | **0.000e+00** | **0.000e+00** | **0.000e+00** | **0.000e+00** | **0.000e+00** | **0.000e+00** | 1 |
|  | **INFO** | **0.000e+00** | **0.000e+00** | **0.000e+00** | **0.000e+00** | **0.000e+00** | **0.000e+00** | 1 |
|  | **AHA** | **0.000e+00** | **0.000e+00** | **0.000e+00** | **0.000e+00** | **0.000e+00** | **0.000e+00** | 1 |
|  | **ARO** | **0.000e+00** | **0.000e+00** | **0.000e+00** | **0.000e+00** | **0.000e+00** | **0.000e+00** | 1 |
|  | **WOA** | **0.000e+00** | **0.000e+00** | **0.000e+00** | **0.000e+00** | **0.000e+00** | **0.000e+00** | 1 |
|  | **SWO** | 0.0074304 | 365.57 | 87.581 | 109.03 | 19.905 | 29.111 | 9 |
|  | **PSO** | 20.541 | 72.512 | 47.802 | 13.412 | 2.4488 | 48.299 | 8 |
|  | **MFO** | 56.786 | 161.48 | 102.71 | 30.967 | 5.6537 | 102.53 | 10 |
|  | **SOA** | **0.000e+00** | **0.000e+00** | **0.000e+00** | **0.000e+00** | **0.000e+00** | **0.000e+00** | 1 |
|  | **SCA** | 0.0015626 | 104.56 | 22.981 | 29.119 | 5.3165 | 11.41 | 7 |
| **F10** | **ARINFO** | **4.4409e-16** | **4.4409e-16** | **4.4409e-16** | **0.000e+00** | **0.000e+00** | **4.4409e-16** | 1 |
|  | **INFO** | **4.4409e-16** | **4.4409e-16** | **4.4409e-16** | **0.000e+00** | **0.000e+00** | **4.4409e-16** | 1 |
|  | **AHA** | **4.4409e-16** | **4.4409e-16** | **4.4409e-16** | **0.000e+00** | **0.000e+00** | **4.4409e-16** | 1 |
|  | **ARO** | **4.4409e-16** | **4.4409e-16** | **4.4409e-16** | **0.000e+00** | **0.000e+00** | **4.4409e-16** | 1 |
|  | **WOA** | **4.4409e-16** | 7.5495e-15 | 3.2863e-15 | 2.1681e-15 | 3.9583e-16 | 3.9968e-15 | 6 |
|  | **SWO** | 0.50367 | 20.264 | 10.291 | 6.3946 | 1.1675 | 9.6122 | 10 |
|  | **PSO** | 0.0083741 | 0.046876 | 0.018138 | 0.0084627 | 0.0015451 | 0.016228 | 7 |
|  | **MFO** | 0.15337 | 19.959 | 6.2227 | 8.7894 | 1.6047 | 0.7816 | 8 |
|  | **SOA** | **4.4409e-16** | **4.4409e-16** | **4.4409e-16** | **0.000e+00** | **0.000e+00** | 4.4409e-16 | 1 |
|  | **SCA** | 0.00066834 | 20.238 | 9.4501 | 9.2887 | 1.6959 | 8.4423 | 9 |
| **F11** | **ARINFO** | **0.000e+00** | **0.000e+00** | **0.000e+00** | **0.000e+00** | **0.000e+00** | **0.000e+00** | 1 |
|  | **INFO** | **0.000e+00** | **0.000e+00** | **0.000e+00** | **0.000e+00** | **0.000e+00** | **0.000e+00** | 1 |
|  | **AHA** | **0.000e+00** | **0.000e+00** | **0.000e+00** | **0.000e+00** | **0.000e+00** | **0.000e+00** | 1 |
|  | **ARO** | **0.000e+00** | **0.000e+00** | **0.000e+00** | **0.000e+00** | **0.000e+00** | **0.000e+00** | 1 |
|  | **WOA** | **0.000e+00** | 0.039241 | 0.0029446 | 0.009258 | 0.0016903 | **0.000e+00** | 1 |
|  | **SWO** | 0.0041487 | 481.08 | 94.39 | 156.75 | 28.618 | 10.05 | 10 |
|  | **PSO** | 9.5064e-06 | 0.04675 | 0.0099742 | 0.0091009 | 0.0016616 | 0.0098761 | 7 |
|  | **MFO** | 0.27375 | 180.16 | 6.654 | 32.771 | 5.9831 | 0.66062 | 9 |
|  | **SOA** | **0.000e+00** | **0.000e+00** | **0.000e+00** | **0.000e+00** | **0.000e+00** | **0.000e+00** | 1 |
|  | **SCA** | 0.012086 | 0.93805 | 0.37431 | 0.29614 | 0.054068 | 0.2748 | 8 |
| **F12** | **ARINFO** | **1.5705e-32** | **1.5705e-32** | **1.5705e-32** | **5.5674e-48** | **1.0165e-48** | **1.5705e-32** | 1 |
|  | **INFO** | 1.8529e-32 | 6.8761e-30 | 8.1547e-31 | 1.5311e-30 | 2.7955e-31 | 1.5575e-31 | 3 |
|  | **AHA** | 2.0214e-08 | 3.7029e-07 | 8.4794e-08 | 7.137e-08 | 1.303e-08 | 6.7268e-08 | 5 |
|  | **ARO** | 4.028e-11 | 1.7442e-10 | 9.1555e-11 | 3.1689e-11 | 5.7856e-12 | 8.4346e-11 | 4 |
|  | **WOA** | 2.5926e-05 | 0.00014559 | 6.3884e-05 | 2.8044e-05 | 5.12e-06 | 5.659e-05 | 7 |
|  | **SWO** | 1.4837 | 4.6395e+08 | 5.0874e+07 | 1.279e+08 | 2.3352e+07 | 6.0664 | 10 |
|  | **PSO** | 8.8043e-07 | 5.916e-05 | 7.324e-06 | 1.1038e-05 | 2.0152e-06 | 3.7656e-06 | 6 |
|  | **MFO** | 0.18957 | 6.4119 | 1.6132 | 1.4178 | 0.25885 | 1.2276 | 8 |
|  | **SOA** | 1.748e-32 | 1.5794e-30 | 1.0781e-31 | 3e-31 | 5.4772e-32 | 2.4055e-32 | 2 |
|  | **SCA** | 0.2985 | 15.496 | 2.6205 | 3.549 | 0.64796 | 1.2382 | 9 |
| **F13** | **ARINFO** | 2.8289e-32 | 2.1659e-14 | 7.2232e-16 | 3.9543e-15 | 7.2195e-16 | 4.1286e-31 | 2 |
|  | **INFO** | 2.2797e-31 | 0.30758 | 0.019601 | 0.055861 | 0.010199 | 0.010987 | 6 |
|  | **AHA** | **1.3498e-32** | **1.3498e-32** | **1.3498e-32** | **5.5674e-48** | **1.0165e-48** | **1.3498e-32** | 1 |
|  | **ARO** | 3.2792e-10 | 0.19774 | 0.016389 | 0.052207 | 0.0095316 | 1.0912e-09 | 5 |
|  | **WOA** | 0.00034023 | 0.012594 | 0.0028093 | 0.0041504 | 0.00075776 | 0.001092 | 4 |
|  | **SWO** | 2.0958 | 2.575e+08 | 9.2724e+06 | 4.6983e+07 | 8.5779e+06 | 18.923 | 10 |
|  | **PSO** | 1.9123e-05 | 0.0015483 | 0.00017314 | 0.00028222 | 5.1526e-05 | 8.9924e-05 | 3 |
|  | **MFO** | 0.2116 | 5.3643 | 1.7207 | 1.5087 | 0.27545 | 1.3791 | 8 |
|  | **SOA** | 6.824e-06 | 1.2847 | 0.36284 | 0.32592 | 0.059504 | 0.20765 | 7 |
|  | **SCA** | 2.1224 | 51.84 | 7.2547 | 10.855 | 1.9818 | 2.9258 | 9 |
| **F14** | **ARINFO** | **0.998** | **0.998** | **0.998** | **0.000e+00** | **0.000e+00** | **0.998** | 1 |
|  | **INFO** | **0.998** | **0.998** | **0.998** | **0.000e+00** | **0.000e+00** | **0.998** | 1 |
|  | **AHA** | **0.998** | **0.998** | **0.998** | **0.000e+00** | **0.000e+00** | **0.998** | 1 |
|  | **ARO** | **0.998** | **0.998** | **0.998** | **0.000e+00** | **0.000e+00** | **0.998** | 1 |
|  | **WOA** | **0.998** | **0.998** | **0.998** | 4.9928e-13 | 9.1156e-14 | **0.998** | 8 |
|  | **SWO** | 1.0029 | 17.754 | 4.9753 | 3.852 | 0.70327 | 3.7079 | 10 |
|  | **PSO** | **0.998** | **0.998** | **0.998** | 1.3039e-16 | 2.3806e-17 | 0.998 | 6 |
|  | **MFO** | **0.998** | **0.998** | **0.998** | **0.000e+00** | **0.000e+00** | **0.998** | 1 |
|  | **SOA** | **0.998** | **0.998** | **0.998** | 1.7494e-16 | 3.1939e-17 | 0.998 | 7 |
|  | **SCA** | **0.998** | 0.99803 | 0.99801 | 4.792e-06 | 8.7489e-07 | **0.998** | 9 |
| **F15** | **ARINFO** | **0.00028518** | **0.00028518** | **0.00028518** | **1.2852e-19** | **2.3465e-20** | **0.00028518** | 1 |
|  | **INFO** | 0.00030749 | 0.0012232 | 0.00058219 | 0.00042679 | 7.7922e-05 | 0.00030749 | 5 |
|  | **AHA** | 0.00030749 | 0.00030749 | 0.00030749 | 3.2759e-19 | 5.9809e-20 | 0.00030749 | 2 |
|  | **ARO** | 0.00030749 | 0.0012232 | 0.0004601 | 0.00034709 | 6.337e-05 | 0.00030749 | 4 |
|  | **WOA** | 0.0003118 | 0.001263 | 0.00063765 | 0.00035504 | 6.4821e-05 | 0.00047681 | 7 |
|  | **SWO** | 0.00076914 | 0.024157 | 0.0095653 | 0.0063372 | 0.001157 | 0.0083879 | 10 |
|  | **PSO** | 0.00030749 | 0.0010715 | 0.00058853 | 0.00028718 | 5.2432e-05 | 0.0004829 | 6 |
|  | **MFO** | 0.00052443 | 0.0012232 | 0.00080477 | 0.00019984 | 3.6485e-05 | 0.00074601 | 8 |
|  | **SOA** | 0.00030749 | 0.00031293 | 0.00030886 | 1.3896e-06 | 2.537e-07 | 0.00030856 | 3 |
|  | **SCA** | 0.00031672 | 0.0013993 | 0.00087974 | 0.00042118 | 7.6896e-05 | 0.00080747 | 9 |
| **F16** | **ARINFO** | **-1.0316** | **-1.0316** | **-1.0316** | **6.5195e-16** | **1.1903e-16** | **-1.0316** | 1 |
|  | **INFO** | **-1.0316** | **-1.0316** | **-1.0316** | 6.7752e-16 | 1.237e-16 | **-1.0316** | 3 |
|  | **AHA** | **-1.0316** | **-1.0316** | **-1.0316** | 6.7122e-16 | 1.2255e-16 | **-1.0316** | 4 |
|  | **ARO** | **-1.0316** | **-1.0316** | **-1.0316** | 6.7752e-16 | 1.237e-16 | **-1.0316** | 5 |
|  | **WOA** | **-1.0316** | **-1.0316** | **-1.0316** | 7.8462e-13 | 1.4325e-13 | **-1.0316** | 8 |
|  | **SWO** | -1.0314 | -0.67231 | -0.94459 | 0.10115 | 0.018467 | -0.99356 | 10 |
|  | **PSO** | **-1.0316** | **-1.0316** | **-1.0316** | **6.5195e-16** | **1.1903e-16** | **-1.0316** | 1 |
|  | **MFO** | **-1.0316** | **-1.0316** | **-1.0316** | 6.7752e-16 | 1.237e-16 | **-1.0316** | 6 |
|  | **SOA** | **-1.0316** | **-1.0316** | **-1.0316** | 3.2215e-13 | 5.8816e-14 | **-1.0316** | 7 |
|  | **SCA** | **-1.0316** | **-1.0316** | **-1.0316** | 7.9787e-06 | 1.4567e-06 | **-1.0316** | 9 |
| **F17** | **ARINFO** | **0.000e+00** | **0.000e+00** | **0.000e+00** | **0.000e+00** | **0.000e+00** | **0.000e+00** | 1 |
|  | **INFO** | 1.1286e-28 | 1.0301e-27 | 4.3369e-28 | 2.5593e-28 | 4.6727e-29 | 4.2333e-28 | 5 |
|  | **AHA** | 3.1893e-80 | 2.9057e-71 | 2.7372e-72 | 6.6956e-72 | 1.2224e-72 | 9.9561e-74 | 3 |
|  | **ARO** | 2.1113e-44 | 1.1831e-41 | 1.4984e-42 | 2.2164e-42 | 4.0466e-43 | 9.9049e-43 | 4 |
|  | **WOA** | 2.2359e-13 | 82.885 | 17.439 | 25.157 | 4.5931 | 2.2624 | 9 |
|  | **SWO** | 0.033563 | 70.581 | 13.034 | 16.228 | 2.9628 | 7.8914 | 8 |
|  | **PSO** | 0.27446 | 0.81329 | 0.45369 | 0.13808 | 0.02521 | 0.40186 | 6 |
|  | **MFO** | 18.815 | 49.27 | 31.812 | 8.366 | 1.5274 | 30.715 | 10 |
|  | **SOA** | 1.641e-118 | 5.511e-112 | 3.693e-113 | 1.0525e-112 | 1.9215e-113 | 1.8039e-114 | 2 |
|  | **SCA** | 3.2475 | 27.348 | 12.078 | 6.9391 | 1.2669 | 9.4223 | 7 |
| **F18** | **ARINFO** | **3.000e+00** | **3.000e+00** | **3.000e+00** | **1.195e-15** | **2.1818e-16** | **3.000e+00** | 1 |
|  | **INFO** | **3.000e+00** | **3.000e+00** | **3.000e+00** | 1.9515e-15 | 3.5629e-16 | **3.000e+00** | 2 |
|  | **AHA** | **3.000e+00** | **3.000e+00** | **3.000e+00** | 1.8199e-15 | 3.3226e-16 | **3.000e+00** | 3 |
|  | **ARO** | **3.000e+00** | **3.000e+00** | **3.000e+00** | 1.855e-15 | 3.3868e-16 | **3.000e+00** | 4 |
|  | **WOA** | **3.000e+00** | **3.000e+00** | **3.000e+00** | 6.0824e-08 | 1.1105e-08 | **3.000e+00** | 8 |
|  | **SWO** | 3.0139 | 13.656 | 4.8143 | 2.7029 | 0.49348 | 3.673 | 10 |
|  | **PSO** | **3.000e+00** | **3.000e+00** | **3.000e+00** | 1.5755e-15 | 2.8765e-16 | **3.000e+00** | 5 |
|  | **MFO** | **3.000e+00** | **3.000e+00** | **3.000e+00** | 1.5406e-15 | 2.8127e-16 | **3.000e+00** | 6 |
|  | **SOA** | **3.000e+00** | **3.000e+00** | **3.000e+00** | 7.9484e-15 | 1.4512e-15 | **3.000e+00** | 7 |
|  | **SCA** | **3.000e+00** | **3.000e+00** | **3.000e+00** | 3.3313e-06 | 6.0821e-07 | **3.000e+00** | 9 |
| **F19** | **ARINFO** | **-12569** | -12154 | **-12556** | 75.775 | 13.835 | **-12569** | 1 |
|  | **INFO** | -10773 | -7968.3 | -9454.7 | 679.74 | 124.1 | -9603.1 | 5 |
|  | **AHA** | -12568 | **-12326** | -12546 | **50.623** | **9.2425** | -12562 | 2 |
|  | **ARO** | -12333 | -9904.3 | -11709 | 497.41 | 90.814 | -11839 | 4 |
|  | **WOA** | **-12569** | -9016.1 | -11855 | 1142.3 | 208.56 | -12461 | 3 |
|  | **SWO** | -4128.1 | -3012.8 | -3570.6 | 325.92 | 59.504 | -3671.8 | 10 |
|  | **PSO** | -7713.2 | -5087 | -6794.3 | 663.34 | 121.11 | -6953 | 8 |
|  | **MFO** | -10784 | -7824.6 | -9100.3 | 751.39 | 137.18 | -9050.5 | 6 |
|  | **SOA** | -10995 | -6321.4 | -7738.1 | 1187.7 | 216.85 | -7390.8 | 7 |
|  | **SCA** | -5003 | -3830.8 | -4277.1 | 319.17 | 58.273 | -4213.9 | 9 |
| **F20** | **ARINFO** | **-3.322** | **-3.322** | **-3.322** | **1.2506e-15** | **2.2834e-16** | **-3.322** | 1 |
|  | **INFO** | **-3.322** | -3.2031 | -3.2467 | 0.058273 | 0.010639 | -3.2031 | 7 |
|  | **AHA** | **-3.322** | -3.2031 | -3.2784 | 0.058273 | 0.010639 | **-3.322** | 3 |
|  | **ARO** | **-3.322** | -3.2031 | -3.2586 | 0.060328 | 0.011014 | -3.2031 | 4 |
|  | **WOA** | **-3.322** | -3.1867 | -3.2524 | 0.061981 | 0.011316 | -3.2022 | 6 |
|  | **SWO** | -3.1979 | -2.302 | -2.7987 | 0.21049 | 0.03843 | -2.812 | 10 |
|  | **PSO** | **-3.322** | -3.2031 | -3.2546 | 0.059923 | 0.01094 | -3.2031 | 5 |
|  | **MFO** | **-3.322** | -3.2031 | -3.215 | 0.036278 | 0.0066234 | -3.2031 | 8 |
|  | **SOA** | **-3.322** | **-3.322** | **-3.322** | 3.1684e-07 | 5.7846e-08 | **-3.322** | 2 |
|  | **SCA** | -3.2014 | -2.6211 | -3.063 | 0.10157 | 0.018545 | -3.1179 | 9 |
| **F21** | **ARINFO** | **-10.153** | **-10.153** | **-10.153** | **6.9035e-15** | **1.2604e-15** | **-10.153** | 1 |
|  | **INFO** | **-10.153** | -2.6305 | -9.6517 | 1.9086 | 0.34846 | **-10.153** | 6 |
|  | **AHA** | **-10.153** | **-10.153** | **-10.153** | 7.174e-15 | 1.3098e-15 | **-10.153** | 2 |
|  | **ARO** | **-10.153** | **-10.153** | **-10.153** | 7.2269e-15 | 1.3194e-15 | **-10.153** | 3 |
|  | **WOA** | **-10.153** | -10.143 | **-10.153** | 0.0019506 | 0.00035612 | **-10.153** | 5 |
|  | **SWO** | -5.0569 | -0.66926 | -1.8882 | 0.96127 | 0.1755 | -1.7049 | 10 |
|  | **PSO** | **-10.153** | -5.0552 | -7.7863 | 2.5736 | 0.46988 | **-10.153** | 8 |
|  | **MFO** | **-10.153** | -5.1008 | -9.648 | 1.5416 | 0.28146 | **-10.153** | 7 |
|  | **SOA** | **-10.153** | **-10.153** | **-10.153** | 3.6353e-05 | 6.6371e-06 | **-10.153** | 4 |
|  | **SCA** | -9.5025 | -0.88148 | -4.8403 | 1.5024 | 0.2743 | -4.9232 | 9 |
| **F22** | **ARINFO** | **-10.403** | **-10.403** | **-10.403** | **1.0175e-15** | **1.6933e-16** | **-10.403** | 1 |
|  | **INFO** | **-10.403** | **-10.403** | **-10.403** | 1.0395e-15 | 2.5551e-16 | **-10.403** | 2 |
|  | **AHA** | **-10.403** | **-10.403** | **-10.403** | 1.0431e-15 | 2.9045e-16 | **-10.403** | 3 |
|  | **ARO** | **-10.403** | **-10.403** | **-10.403** | 1.714e-15 | 3.1293e-16 | **-10.403** | 4 |
|  | **WOA** | **-10.403** | -2.7659 | -9.9711 | 1.6711 | 0.3051 | **-10.403** | 7 |
|  | **SWO** | -4.5538 | -1.0764 | -2.2713 | 0.9338 | 0.17049 | -1.9791 | 10 |
|  | **PSO** | **-10.403** | -5.0877 | -9.3454 | 2.1513 | 0.39277 | **-10.403** | 8 |
|  | **MFO** | **-10.403** | -5.0877 | -10.226 | 0.97043 | 0.17718 | **-10.403** | 6 |
|  | **SOA** | **-10.403** | **-10.403** | **-10.403** | 3.3636e-05 | 6.1411e-06 | **-10.403** | 5 |
|  | **SCA** | -8.2099 | -0.90896 | -5.1494 | 1.2821 | 0.23408 | -4.9737 | 9 |
| **F23** | **ARINFO** | **-10.536** | **-10.536** | **-10.536** | **1.8067e-16** | **3.2986e-16** | **-10.536** | 1 |
|  | **INFO** | **-10.536** | -2.4217 | -10.266 | 1.4815 | 0.27049 | **-10.536** | 5 |
|  | **AHA** | **-10.536** | -10.536 | **-10.536** | 1.7749e-15 | 3.4596e-16 | **-10.536** | 2 |
|  | **ARO** | **-10.536** | **-10.536** | **-10.536** | 1.8964e-15 | 3.5432e-16 | **-10.536** | 3 |
|  | **WOA** | **-10.536** | -2.8066 | -9.8673 | 2.0385 | 0.37218 | **-10.536** | 7 |
|  | **SWO** | -4.9337 | -1.3093 | -2.322 | 0.94136 | 0.17187 | -2.0303 | 10 |
|  | **PSO** | **-10.536** | -5.1285 | -9.9988 | 1.6405 | 0.29952 | **-10.536** | 6 |
|  | **MFO** | **-10.536** | -5.1285 | -9.8185 | 1.8616 | 0.33989 | **-10.536** | 8 |
|  | **SOA** | **-10.536** | **-10.536** | **-10.536** | 2.8714e-05 | 5.2425e-06 | **-10.536** | 4 |
|  | **SCA** | -8.3638 | -4.1721 | -5.5781 | 1.1307 | 0.20644 | -5.0307 | 9 |

##### **Table B2.** Average ranks and overall rankings of competing algorithms on the CEC 2017 benchmark suite

| **Optimizer** | **ARINFO** | **INFO** | **AHA** | **ARO** | **WOA** | **SWO** | **PSO** | **MFO** | **SOA** | **SCA** |
| --- | --- | --- | --- | --- | --- | --- | --- | --- | --- | --- |
| **Average Rank** | 1.3 | 4.13 | 2.65 | 3.61 | 6.17 | 9.78 | 6.04 | 7.43 | 3.48 | 8.30 |
| **Final Ranking** | **1** | 5 | 2 | 4 | 7 | 10 | 6 | 8 | 3 | 9 |

##### **Table B3.** The statistical results of the competitive techniques for the CEC-2022 test suite

| **Function** | **Technique** | **Best** | **Worst** | **Mean** | **SD** | **SE** | **Median** | **Rank** |
| --- | --- | --- | --- | --- | --- | --- | --- | --- |
| **CEC01** | **ARINFO** | **300e+00** | **300e+00** | **300e+00** | **0.000e+00** | **0.000e+00** | **300e+00** | 1 |
|  | **INFO** | **300e+00** | **300e+00** | **300e+00** | 3.2819e-14 | 1.0378e-14 | **300e+00** | 2 |
|  | **AHA** | **300e+00** | **300e+00** | **300e+00** | 1.5356e-05 | 4.8561e-06 | **300e+00** | 5 |
|  | **ARO** | **300e+00** | **300e+00** | **300e+00** | 3.8837e-09 | 1.2281e-09 | **300e+00** | 3 |
|  | **WOA** | 2105.4 | 1.491e+04 | 8.878e+03 | 4.7005e+03 | 1.4864e+03 | 9.4253e+03 | 9 |
|  | **SWO** | 1.2751e+04 | 2.6909 e+04 | 1.9104e+04 | 4.363e+03 | 1379.7 | 1.9854e+04 | 10 |
|  | **PSO** | **300e+00** | **300e+00** | **300e+00** | 1.7348e-08 | 5.4859e-09 | **300e+00** | 4 |
|  | **MFO** | **300e+00** | 7.5121e+03 | 1.5999e+03 | 2.4805e+03 | 784.428963 | **300e+00** | 7 |
|  | **SOA** | 578.80126 | 4.7797e+03 | 2.0129e+03 | 1.1947e+03 | 377.8236 | 1.8066e+03 | 8 |
|  | **SCA** | 529.69329 | 3.0071e+03 | 1.019e+03 | 734.4479 | 232.25281 | 788.17564 | 6 |
| **CEC02** | **ARINFO** | **400e+00** | 408.92 | 404.18 | 2.982 | 0.94299 | 403.99 | 3 |
|  | **INFO** | **400e+00** | 408.92 | 405.26 | 4.1242 | 1.3042 | 406.45 | 4 |
|  | **AHA** | **400e+00** | **400.1 e+00** | **400.03e+00** | **0.031272** | **0.009889** | **400.01e+00** | 1 |
|  | **ARO** | **400e+00** | 470.78 | 407.88 | 22.162 | 7.0083 | 400.01 | 5 |
|  | **WOA** | 400.5 | 489.3 | 435.1 | 38.25 | 12.096 | 408.26 | 7 |
|  | **SWO** | 616.52 | 1908.4 | 1207.2 | 401.38 | 126.93 | 1260.2 | 10 |
|  | **PSO** | 400 | 401.53 | 400.34 | 0.50421 | 0.15944 | 400.11 | 2 |
|  | **MFO** | 406.43 | 408.92 | 408 | 0.92604 | 0.29284 | 408 | 6 |
|  | **SOA** | 404.55 | 510.46 | 452.16 | 33.493 | 10.591 | 452.62 | 9 |
|  | **SCA** | 433.64 | 470.97 | 451.76 | 11.621 | 3.6749 | 452.68 | 8 |
| **CEC03** | **ARINFO** | **600e+00** | **600e+00** | **600e+00** | **8.9615e-08** | **2.8339e-08** | **600e+00** | 1 |
|  | **INFO** | **600e+00** | **600e+00** | **600e+00** | 0.00089128 | 0.00028185 | **600e+00** | 5 |
|  | **AHA** | **600e+00** | **600e+00** | **600e+00** | 1.5239e-05 | 4.819e-06 | **600e+00** | 4 |
|  | **ARO** | **600e+00** | **600e+00** | **600e+00** | 3.6429e-06 | 1.152e-06 | **600e+00** | 3 |
|  | **WOA** | 613.95 | 642.25 | 624.42 | 10.319 | 3.2632 | 619.99 | 9 |
|  | **SWO** | 641.38 | 682.19 | 653.76 | 12.641 | 3.9976 | 648.08 | 10 |
|  | **PSO** | 600 | 616.43 | 604.78 | 5.1846 | 1.6395 | 603.07 | 6 |
|  | **MFO** | **600e+00** | **600e+00** | **600e+00** | 5.9729e-07 | 1.8888e-07 | **600e+00** | 2 |
|  | **SOA** | 610.47 | 624.45 | 617.3 | 4.34 | 1.3724 | 617.4 | 8 |
|  | **SCA** | 613.63 | 618.13 | 615.75 | 1.7937 | 0.56723 | 615.07 | 7 |
| **CEC04** | **ARINFO** | **804.97** | **821.41** | **811.93** | **3.5428** | **1.0436** | **811.92** | 1 |
|  | **INFO** | 807.96 | 824.87 | 816.62 | 5.8778 | 1.8587 | 814.43 | 5 |
|  | **AHA** | 809.95 | 832.83 | 818.9 | 6.5328 | 2.0658 | 817.91 | 6 |
|  | **ARO** | 804.97 | 830.84 | 812.14 | 7.4721 | 2.3629 | 811.94 | 2 |
|  | **WOA** | 817.99 | 853.74 | 831.38 | 10.774 | 3.4069 | 829.46 | 8 |
|  | **SWO** | 851.78 | 888.64 | 873.38 | 12.688 | 4.0124 | 874.9 | 10 |
|  | **PSO** | 806.96 | 819.9 | 813.23 | 3.7243 | 1.1777 | 813.43 | 3 |
|  | **MFO** | 806.96 | 848.75 | 820.88 | 14.179 | 4.4836 | 814.59 | 7 |
|  | **SOA** | 806.97 | 821.89 | 814.03 | 6.4727 | 2.0469 | 814.43 | 4 |
|  | **SCA** | 824.82 | 840.09 | 834.2 | 4.8202 | 1.5243 | 835.55 | 9 |
| **CEC05** | **ARINFO** | **900e+00** | **900e+00** | **900e+00** | **1.3684e-12** | **4.3274e-13** | **900e+00** | 1 |
|  | **INFO** | **900e+00** | 904.62 | 901.12 | 1.6747 | 0.52959 | 900.13 | 5 |
|  | **AHA** | **900e+00** | **900e+00** | **900e+00** | 4.5128e-08 | 1.4271e-08 | **900e+00** | 3 |
|  | **ARO** | **900e+00** | **900e+00** | **900e+00** | 4.6943e-09 | 1.4845e-09 | **900e+00** | 2 |
|  | **WOA** | 981.69 | 1855.1 | 1247.7 | 274.8 | 86.898 | 1193.5 | 9 |
|  | **SWO** | 1233.7 | 2823.1 | 2047 | 436.47 | 138.02 | 2044.4 | 10 |
|  | **PSO** | **900e+00** | 900.63 | 900.15 | 0.25303 | 0.080014 | **900e+00** | 4 |
|  | **MFO** | **900e+00** | 908.13 | 901.15 | 2.5862 | 0.81783 | **900e+00** | 6 |
|  | **SOA** | 928.1 | 1016.7 | 959.66 | 24.899 | 7.8737 | 956.74 | 7 |
|  | **SCA** | 933.01 | 1088.7 | 974.42 | 44.288 | 14.005 | 967.77 | 8 |
| **CEC06** | **ARINFO** | **1800.1** | **1801.3** | **1802.7** | **2.1592** | **0.42898** | **1801.2** | 1 |
|  | **INFO** | 1802.2 | 1907.8 | 1824 | 23.626 | 4.3135 | 1818 | 4 |
|  | **AHA** | **1800.1** | 1808.5 | 1802.4 | 2.3719 | 0.43305 | 1801.4 | 2 |
|  | **ARO** | 1800.2 | 1813.6 | 1803.7 | 3.7144 | 0.67815 | 1802.1 | 3 |
|  | **WOA** | 1868 | 8118.4 | 3445.5 | 1813 | 331.01 | 2872.8 | 7 |
|  | **SWO** | 3.1838e+06 | 4.6907e+08 | 1.1714e+08 | 1.1412e+08 | 2.0835e+07 | 8.7981e+07 | 10 |
|  | **PSO** | 1801.6 | 7895.7 | 2949.1 | 1522.8 | 278.03 | 2198.1 | 6 |
|  | **MFO** | 1883.6 | 8186.3 | 5006.8 | 2412.1 | 440.39 | 4933.3 | 8 |
|  | **SOA** | 1851.9 | 2798.3 | 2101 | 233.66 | 42.66 | 2051.1 | 5 |
|  | **SCA** | 1.2204e+05 | 4.5674e+06 | 1.0623e+06 | 9.4358e+05 | 1.7227e+05 | 9.0507e+05 | 9 |
| **CEC07** | **ARINFO** | **2000** | **2020** | **2002.1** | **4.1233** | **0.90537** | **2000** | 1 |
|  | **INFO** | 2001 | 2024.6 | 2016.8 | 8.2492 | 1.5061 | 2021 | 4 |
|  | **AHA** | **2000** | **2020** | 2005.2 | 8.3589 | 1.5261 | 2001 | 3 |
|  | **ARO** | **2000** | **2020** | 2005.7 | 4.9889 | 0.91084 | **2000** | 2 |
|  | **WOA** | 2021.1 | 2125.5 | 2051.7 | 19.955 | 3.6433 | 2050.3 | 9 |
|  | **SWO** | 2071.6 | 2207.5 | 2135.4 | 31.692 | 5.7862 | 2136.1 | 10 |
|  | **PSO** | 2001 | 2039.9 | 2023 | 10.965 | 2.002 | 2024.2 | 6 |
|  | **MFO** | 2001 | 2024.9 | 2020.8 | 5.299 | 0.96746 | 2021.9 | 5 |
|  | **SOA** | 2015.4 | 2040.7 | 2030.6 | 6.7961 | 1.2408 | 2030.1 | 7 |
|  | **SCA** | 2035.9 | 2061.6 | 2048.9 | 6.9374 | 1.2666 | 2048.2 | 8 |
| **CEC08** | **ARINFO** | **2200** | **2220.7** | **2208.6** | **1.7056** | **0.40272** | **2220** | 1 |
|  | **INFO** | **2200** | 2221.9 | 2217.5 | 7.4061 | 1.3522 | 2220.5 | 3 |
|  | **AHA** | **2200** | **2220.7** | **2208.6** | **1.7056** | **0.40272** | **2200** | 1 |
|  | **ARO** | 2200.1 | **2220.7** | 2219.4 | 3.6594 | 0.66811 | **2220** | 4 |
|  | **WOA** | 2223.5 | 2247.1 | 2231.6 | 5.6938 | 1.0395 | 2230.5 | 9 |
|  | **SWO** | 2242.2 | 2398.4 | 2281.6 | 39.836 | 7.273 | 2266.7 | 10 |
|  | **PSO** | 2200.1 | 2221.7 | 2219.9 | 3.7498 | 0.68462 | 2220.5 | 5 |
|  | **MFO** | 2220.1 | 2227 | 2222.7 | 2.0269 | 0.37006 | 2222.4 | 7 |
|  | **SOA** | 2209.9 | 2224.4 | 2221.8 | 3.0598 | 0.55863 | 2222.6 | 6 |
|  | **SCA** | 2219.2 | 2235.8 | 2230.3 | 3.2788 | 0.59862 | 2230.7 | 8 |
| **CEC09** | **ARINFO** | 2529.3 | 2529.3 | 2529.3 | **0.000e+00** | **0.000e+00** | 2529.3 | 2 |
|  | **INFO** | 2529.3 | 2529.3 | 2529.3 | **0.000e+00** | **0.000e+00** | 2529.3 | 2 |
|  | **AHA** | 2529.3 | 2529.3 | 2529.3 | 6.1352e-06 | 1.1201e-06 | 2529.3 | 5 |
|  | **ARO** | 2529.3 | 2529.3 | 2529.3 | 1.2171e-06 | 2.2222e-07 | 2529.3 | 4 |
|  | **WOA** | 2529.3 | 2621.7 | 2539.6 | 23.05 | 4.2082 | 2529.9 | 7 |
|  | **SWO** | 2582.2 | 2852.2 | 2737 | 62.008 | 11.321 | 2729.5 | 10 |
|  | **PSO** | **2485.5** | **2485.5** | **2485.5** | 3.3291e-08 | 6.078e-09 | **2485.5** | 1 |
|  | **MFO** | 2529.3 | 2529.8 | 2529.3 | 0.10151 | 0.018533 | 2529.3 | 6 |
|  | **SOA** | 2597 | 2674.3 | 2641.1 | 19.777 | 3.6107 | 2645.9 | 9 |
|  | **SCA** | 2538.2 | 2569.3 | 2550.6 | 9.1179 | 1.6647 | 2550.1 | 8 |
| **CEC10** | **ARINFO** | **2500.2** | **2500.4** | **2500.3** | **0.054435** | **0.0099384** | **2500.3** | 1 |
|  | **INFO** | **2500.2** | 2623.2 | 2512 | 35.624 | 6.504 | 2500.4 | 5 |
|  | **AHA** | 2500.5 | 2638 | 2545 | 58.996 | 10.771 | 2501.2 | 9 |
|  | **ARO** | 2500.1 | 2614.4 | 2525.8 | 47.122 | 8.6032 | 2500.3 | 7 |
|  | **WOA** | **2500.2** | 2890.3 | 2535.1 | 82.803 | 15.118 | 2500.8 | 8 |
|  | **SWO** | 2513.6 | 3188 | 2600.4 | 133.2 | 24.319 | 2553.9 | 10 |
|  | **PSO** | 2500.8 | 2628.8 | 2524.8 | 49.988 | 9.1265 | 2500.2 | 6 |
|  | **MFO** | **2500.2** | 2500.9 | 2500.6 | 0.15244 | 0.027832 | 2500.6 | 2 |
|  | **SOA** | 2500.4 | 2512.6 | 2502.6 | 3.078 | 0.56197 | 2501.4 | 4 |
|  | **SCA** | 2500.9 | 2506.8 | 2501.8 | 1.0285 | 0.18778 | 2501.6 | 3 |
| **CEC11** | **ARINFO** | **2600** | **2750.4** | **2605** | **27.464** | **5.014226** | **2600** | 1 |
|  | **INFO** | **2600** | 3183.7 | 2872.8 | 114.92 | 20.982 | 2900 | 7 |
|  | **AHA** | 2602.9 | 2914.3 | 2654.3 | 91.208 | 16.652 | 2605.3 | 3 |
|  | **ARO** | **2600** | 3000 | 2653.3 | 122.43 | 22.352 | **2600** | 2 |
|  | **WOA** | 2601.7 | 2935.4 | 2903.2 | 57.597 | 10.516 | 2914 | 9 |
|  | **SWO** | 3758.5 | 49374 | 8369.3 | 10125 | 1848.5 | 5078.4 | 10 |
|  | **PSO** | **2600** | 4282.7 | 2874.5 | 294.16 | 53.705 | 2900 | 8 |
|  | **MFO** | **2600** | 3465.5 | 2872.2 | 161.52 | 29.49 | 2900 | 6 |
|  | **SOA** | 2604.2 | 3204.3 | 2815.7 | 144.78 | 26.434 | 2767.2 | 4 |
|  | **SCA** | 2760.9 | 3355.4 | 2855.6 | 178.43 | 32.577 | 2784.8 | 5 |
| **CEC12** | **ARINFO** | 2862.6 | 2866.2 | 2864.6 | **0.85973** | **0.15696** | 2864.9 | 3 |
|  | **INFO** | 2858.6 | 2865.8 | 2863.5 | 1.5454 | 0.28215 | 2863.5 | 2 |
|  | **AHA** | 2862.6 | 2875.2 | 2865.5 | 2.4205 | 0.44191 | 2865.2 | 4 |
|  | **ARO** | 2862.7 | 2888.2 | 2866.4 | 4.8114 | 0.87844 | 2865 | 5 |
|  | **WOA** | 2862.1 | 2931.4 | 2877.7 | 18.224 | 3.3272 | 2869.7 | 8 |
|  | **SWO** | 2917.7 | 3089.1 | 2982.6 | 48.164 | 8.7934 | 2970.8 | 10 |
|  | **PSO** | **2849.5** | 2951.1 | 2872.3 | 27.152 | 4.9572 | **2857.5** | 7 |
|  | **MFO** | 2859.4 | **2863.5** | **2862.1** | 0.92906 | 0.16962 | 2862.6 | 1 |
|  | **SOA** | 2877.3 | 2948.5 | 2912.2 | 19.917 | 3.6363 | 2910.4 | 9 |
|  | **SCA** | 2866.5 | 2872.5 | 2869.1 | 1.4329 | 0.26161 | 2869 | 6 |

##### **Table B4.** Average ranks and overall rankings of competing algorithms on the CEC 2022 benchmark suite

| **Optimizer** | **ARINFO** | **INFO** | **AHA** | **ARO** | **WOA** | **SWO** | **PSO** | **MFO** | **SOA** | **SCA** |
| --- | --- | --- | --- | --- | --- | --- | --- | --- | --- | --- |
| **Average Rank** | 1.58 | 4.75 | 3.75 | 3.33 | 8.17 | 10.00 | 4.67 | 5.33 | 7.25 | 7.08 |
| **Final Ranking** | **1** | 5 | 3 | 2 | 9 | 10 | 4 | 6 | 8 | 7 |

|  |  |  |
| --- | --- | --- |
|  |  |  |
|  |  |  |
|  |  |  |
|  |  |  |
|  |  |  |
|  |  |  |
|  |  |  |
|  | | |

**Fig B2.** The convergence curves of the ARINFO and the other optimization methods for the CEC-2017 test.

|  |  |  |
| --- | --- | --- |
|  |  |  |
|  |  |  |
|  |  |  |
|  | | |

**Fig B3.** The convergence curves of the ARINFO and the other optimization methods for the CEC-2022 test.

|  |  |  |
| --- | --- | --- |
|  |  |  |
|  |  |  |
|  |  |  |
|  |  |  |
|  |  |  |
|  |  |  |
|  |  |  |

**Fig B4.** Box plots for the CEC-2017 benchmark functions.

|  |  |  |
| --- | --- | --- |
|  |  |  |
|  |  |  |
|  |  |  |

**Fig B5.** Box plots for the CEC-2022 functions.

##### **Table B5.** Statistical results obtained from the Wilcoxon rank-sum test on 23 CEC2017 benchmark functions.

| **ARINFO vs.** | **INFO** | | **AHA** | | **ARO** | | **WOA** | | **SWO** | | **PSO** | | **MFO** | | **SOA** | | **SCA** | |
| --- | --- | --- | --- | --- | --- | --- | --- | --- | --- | --- | --- | --- | --- | --- | --- | --- | --- | --- |
| F(x) | **P** | **Win** | **P** | **Win** | **P** | **Win** | **P** | **Win** | **P** | **Win** | **P** | **Win** | **P** | **Win** |  | **Win** | **p** | **Win** |
| F1 | 1.2118e-12 | + | 1.2118e-12 | + | 1.2118e-12 | + | 1.2118e-12 | + | 1.2118e-12 | + | 1.2118e-12 | + | 1.2118e-12 | + | 1.2118e-12 | + | 1.2118e-12 | + |
| F2 | 1.2118e-12 | + | 1.2118e-12 | + | 1.2118e-12 | + | 1.2118e-12 | + | 1.2118e-12 | + | 1.2118e-12 | + | 1.2118e-12 | + | 1.2118e-12 | + | 1.2118e-12 | + |
| F3 | 1.2118e-12 | + | 1.2118e-12 | + | 1.2118e-12 | + | 1.2118e-12 | + | 1.2118e-12 | + | 1.2118e-12 | + | 1.2118e-12 | + | 1.2118e-12 | + | 1.2118e-12 | + |
| F4 | 1.2118e-12 | + | 1.2118e-12 | + | 1.2118e-12 | + | 1.2118e-12 | + | 1.2118e-12 | + | 1.2118e-12 | + | 1.2118e-12 | + | 1.2118e-12 | + | 1.2118e-12 | + |
| F5 | 3.0199e-11 | + | 3.0199e-11 | + | 3.0199e-11 | + | 3.3384e-11 | + | 3.0199e-11 | + | 3.0199e-11 | + | 3.0199e-11 | + | 1.2118e-12 | + | 3.0199e-11 | + |
| F6 | 8.7902e-04 | + | 3.0180e-11 | + | 3.0180e-11 | + | 3.0180e-11 | + | 3.0180e-11 | + | 3.0180e-11 | + | 3.0180e-11 | + | 1.2108e-12 | + | 3.0180e-11 | + |
| F7 | 1.3853e-06 | + | 2.81298e-02 | = | 5.462e-06 | + | 2.0058e-04 | + | 3.0199e-11 | + | 3.0199e-11 | + | 3.0199e-11 | + | 2.1959e-07 | + | 3.0199e-11 | + |
| F8 | 4.0746e-11 | + | 6.0621e-11 | + | 5.4907e-11 | + | 4.0829e-05 | + | 3.0180e-11 | + | 3.0180e-11 | + | 6.6915e-11 | + | 5.5696e-10 | + | 3.0180e-11 | + |
| F9 | NaN | **=** | NaN | = | NaN | = | NaN | = | 1.2118e-12 | + | 1.2118e-12 | + | 1.2118e-12 | + | NaN | = | 1.2118e-12 | + |
| F10 | NaN | **=** | NaN | = | NaN | = | 2.6420e-08 | + | 1.2118e-12 | + | 1.2118e-12 | + | 1.2118e-12 | + | NaN | = | 1.2118e-12 | + |
| F11 | NaN | **=** | NaN | = | NaN | = | 8.8152e-12 | + | 1.2118e-12 | + | 1.2118e-12 | + | 1.2118e-12 | + | NaN | = | 1.2118e-12 | + |
| F12 | 6.0654e-06 | + | 6.0654e-06 | + | 6.0654e-06 | + | 6.0654e-06 | + | 6.0654e-06 | + | 6.0654e-06 | + | 6.0654e-06 | + | 1.2118e-12 | + | 3.0199e-11 | + |
| F13 | 1.7233e-06 | + | 3.0161e-11 | + | 3.0161e-11 | + | 3.0161e-11 | + | 3.0161e-11 | + | 3.0161e-11 | + | 3.0161e-11 | + | 1.2098e-12 | + | 3.0161e-11 | + |
| F14 | NaN | **=** | NaN | = | NaN | = | 1.2108e-12 | + | 1.2108e-12 | + | 1.608e-2 | = | NaN | = | 1.1341e-03 | + | 1.2118e-12 | + |
| F15 | 3.3048e-02 | + | 3.6443e-08 | + | 4.5809e-05 | + | 2.8056e-11 | + | 2.8056e-11 | + | 2.8056e-11 | + | 2.7845e-11 | + | 2.8056e-11 | + | 2.8056e-11 | + |
| F16 | 1.04188e-4 | + | 1.16948e-01 | = | 4. 1838e-2 | = | 5.0325e-13 | + | 4.0806e-12 | + | 1 | - | 4.1895e-13 | - | 2.6943e-06 | + | 4.0806e-12 | + |
| F17 | 1.2118e-12 | + | 1.2118e-12 | + | 1.2118e-12 | - | 1.2118e-12 | + | 1.2118e-12 | + | 1.2118e-12 | + | 1.2118e-12 | + | 1.2118e-12 | + | 1.2118e-12 | + |
| F18 | 6.7498e-04 | + | 3.15588e-4 | + | 2.4698e-08 | + | 9.3223e-12 | + | 9.3223e-12 | + | 2.608e-12 | + | 5.164e-14 | - | 1.219e-05 | + | 9.3223e-12 | + |
| F19 | 3.0199e-11 | + | 5.5727e-11 | + | 4.0746e-11 | + | 2.1544e-12 | + | 3.0199e-11 | + | 3.0199e-11 | + | 3.0199e-11 | + | 3.0199e-11 | + | 3.0199e-11 | + |
| F20 | 3.23588e-12 | + | 7.4075e-04 | + | 1.2088e-02 | = | 4.4445e-06 | + | 1.6889e-13 | + | 1.2690e-13 | + | 1.9693e-05 | + | 7.3644e-3 | - | 1.6889e-11 | + |
| F21 | 4.16048e-12 | + | 4.04768e-2 | + | 2.3371e-04 | + | 1.7203e-12 | + | 1.7203e-12 | + | 3.6751e-11 | + | 2.897e-11 | + | 1.7203e-12 | + | 1.7203e-12 | + |
| F22 | 3.60138e-12 | + | 1.01078e-04 | + | 3.3042e-05 | + | 1.1364e-11 | + | 1.1364e-11 | + | 2.2372e-06 | + | 6.4931e-11 | + | 1.1364e-11 | + | 1.1364e-11 | + |
| F23 | 1 | - | 3.33758e-08 | + | 3.33758e-08 | + | 1.2118e-12 | + | 1.2118e-12 | + | 4.6533e-06 | + | 7.2984e-03 | + | 1.2118e-12 | + | 1.2118e-12 | + |
| **WRST (+/=/-)** | **18/4/1** | | **17/6/0** | | **16/6/1** | | **22/1/0** | | **23/0/0** | | **21/1/1** | | **20/1/2** | | **19/3/1** | | **23/0/0** | |

##### **Table B6.** Statistical results obtained from the Wilcoxon rank-sum test on 12 CEC2022 benchmark functions.

| **ARINFO vs.** | **INFO** | | **AHA** | | **ARO** | | **WOA** | | **SWO** | | **PSO** | | **MFO** | | **SOA** | | **SCA** | |
| --- | --- | --- | --- | --- | --- | --- | --- | --- | --- | --- | --- | --- | --- | --- | --- | --- | --- | --- |
| F(x) | **P** | **Win** | **P** | **Win** | **P** | **Win** | **P** | **Win** | **P** | **Win** | **P** | **Win** | **P** | **Win** | **P** | **Win** | **P** | **Win** |
| CEC01 | 7.6716e-03 | + | 6.3864e-05 | + | 6.3864e-05 | + | 6.3864e-05 | + | 6.3864e-05 | + | 6.3864e-05 | + | 6.3864e-05 | + | 6.3864e-05 | + | 6.3864e-05 | + |
| CEC02 | 2.4197e-04 | + | 2.4534e-04 | + | 1.4690e-02 | = | 6.0107e-04 | + | 1.6118e-04 | + | 2.45114e-02 | = | 9.6216e-04 | + | 5.2380e-04 | + | 1.6118e-04 | + |
| CEC03 | 2.3721e-04 | + | 1.1329e-02 | = | 9.6844e-04 | + | 1.3173e-04 | + | 1.3173e-04 | + | 4.4099e-04 | + | 5.8521e-08 | + | 1.3173e-04 | + | 1.3173e-04 | + |
| CEC04 | 3.836e-05 | + | 1.8594e-08 | + | 1.4710e-03 | + | 1.1371e-04 | + | 1.8267e-14 | + | 9.0973e-08 | + | 4.0551e-04 | + | 8.708e-14 | - | 1.8267e-04 | + |
| CEC05 | 1.1966e-04 | + | 1.3732e-02 | = | 1.1373e-02 | = | 1.6211e-04 | + | 1.6211e-04 | + | 1.5784e-02 | = | 1 | - | 1.6211e-04 | + | 1.6211e-04 | + |
| CEC06 | 1.3111e-08 | + | 5.3951e-04 | + | 4.5537e-04 | + | 3.0199e-11 | + | 3.0199e-11 | + | 1.4643e-10 | + | 3.0199e-11 | + | 3.0199e-11 | + | 3.0199e-11 | + |
| CEC07 | 2.1518e-08 | + | 6.4122e-08 | + | 6.3071e-06 | + | 2.9376e-11 | + | 2.9376e-11 | + | 4.4748e-09 | + | 1.7312e-10 | + | 1.1748e-10 | + | 2.9376e-11 | + |
| CEC08 | 8.5641e-04 | + | 1.4941e-04 | + | 4.7394e-11 | + | 3.0199e-11 | + | 3.0199e-11 | + | 2.6806e-04 | + | 7.3803e-10 | + | 3.8249e-09 | + | 1.6132e-10 | + |
| CEC09 | NaN | = | 1.2118e-12 | + | 1.2118e-12 | + | 1.2118e-12 | + | 1.2118e-12 | + | 1.2118e-12 | + | 1.3337e-04 | + | 1.2118e-12 | + | 1.2118e-12 | + |
| CEC10 | 6.2828e-06 | + | 3.0199e-11 | + | 8.30261e-04 | + | 3.8202e-10 | + | 3.0199e-11 | + | 4.46756e-11 | + | 5.5727e-10 | + | 3.0199e-11 | + | 3.0199e-11 | + |
| CEC11 | 8.1622e-08 | + | 2.6099e-10 | + | 9.5139e-06 | + | 3.3384e-11 | + | 3.0199e-11 | + | 4.444e-07 | + | 6.6955e-11 | + | 8.9934e-11 | + | 3.0199e-11 | + |
| CEC12 | 2.325e-04 | - | 2.1519e-04 | + | 2.2263e-08 | + | 4.3106e-08 | + | 3.0199e-11 | + | 4.1023e-04 | - | 1.1574e-10 | + | 3.0199e-11 | + | 3.0199e-11 | + |
| **WRST (+/=/-)** | **10/1/1** | | **10/2/0** | | **10/2/0** | | **12/0/0** | | **12/0/0** | | **9/2/1** | | **11/0/1** | | **11/0/1** | | **12/0/0** | |

##### **Table B7.** P-value-based statistical metrics for the CEC- 2017 benchmark function

|  | **F1** | **F2** | **F3** | **F4** | **F5** | **F6** |
| --- | --- | --- | --- | --- | --- | --- |
| P- value based ANOVA | 4.5985e−182 | 8.47901e−151 | 4.3698e−118 | 2.3394e−102 | 7.5947e−83 | 5.8175e−68 |
| P- value based Friedman | 4.6372e-59 | 6.1388e-68 | 7.8944e-45 | 4.7968e-34 | 9.3991e-40 | 8.6168e-56 |
| P- value based Krukal | 8.3967e-69 | 3.2846e-87 | 5.4298e-65 | 2.4581e-69 | 8.4219e-85 | 5.4322e-60 |
|  | **F7** | **F8** | **F9** | **F10** | **F11** | **F12** |
| P- value based ANOVA | 3.5049e−71 | 6.7023e−62 | 4.9841e−91 | 4.98671e−105 | 4.7570e−38 | 1.6344e−37 |
| P- value based Friedman | 5.9585e−32 | 6.97312e−49 | 2.45801e−68 | 9.5561e−82 | 7.4199e−78 | 2.2914e−81 |
| P- value based Krukal | 5.3696e−72 | 3.42351e-89 | 1.17841e−61 | 5.4211e−96 | 4.7983e−77 | 9.4211e−80 |
|  | **F13** | **F14** | **F15** | **F16** | **F17** | **F18** |
| P- value based ANOVA | 3.4784e−42 | 1.9880e−58 | 8.9995e−77 | 4.5575e−89 | 5.3249e−71 | 2.8701e−31 |
| P- value based Friedman | 1.24976e−50 | 7.0412e−62 | 1.8815e−32 | 8.2576e−52 | 1.7290e−57 | 4.1196e−79 |
| P- value based Krukal | 6.2384e−188 | 4.7921e−73 | 3.5491e−114 | 3.4579e−96 | 3.4579e−125 | 5.9968e−84 |
|  | **F19** | **F20** | **F21** | **F22** | **F23** |  |
| P- value based ANOVA | 9.3640e−37 | 2.1874e−77 | 2.3391e−48 | 6.3675e−85 | 8.7448e−29 |  |
| P- value based Friedman | 8.0222e−53 | 1.3962e−44 | 4.7680e−63 | 5.5231e−71 | 4.4159e−33 |  |
| P- value based Krukal | 6.9208e−41 | 8.1196e−23 | 7.8122e−59 | 4.8812e−48 | 3.6842e−31 |  |

##### **Table B8.** P-value-based statistical metrics for the CEC-2022 benchmark function

|  | **CEC01** | **CEC02** | **CEC03** | **CEC04** | **CEC05** | **CEC06** |
| --- | --- | --- | --- | --- | --- | --- |
| P- value based ANOVA | 2.7417e−127 | 5.6895e−112 | 2.4894e−38 | 3.8133e−69 | 7.9952e−41 | 4.7265e−26 |
| P- value based Friedman | 1.3714e−34 | 9.6728e−45 | 5.8373e−82 | 2.3039e−31 | 3.7881e−107 | 6.8497e−58 |
| P- value based Krukal | 1.1329e−28 | 4.5828e−30 | 9.8147e−18 | 6.8077e−37 | 4.6718e−55 | 5.5277e−20 |
|  | **CEC07** | **CEC08** | **CEC09** | **CEC10** | **CEC11** | **CEC12** |
| P- value based ANOVA | 1.6787e−109 | 8.5244 e−98 | 1.9745e−101 | 7.8300e−71 | 8.7856e−34 | 1.4531e−18 |
| P- value based Friedman | 2.4191e−41 | 8.7501e−46 | 5.6655e−28 | 3.8075e−42 | 2.6177e−151 | 1.8183e−32 |
| P- value based Krukal | 1.4856e−85 | 5.5679e−20 | 1.5473e−33 | 6.8411e−87 | 8.54721e−28 | 1.2183e−27 |
